# The evolution of WRKY transcription factors

**DOI:** 10.1186/s12870-015-0456-y

**Published:** 2015-02-27

**Authors:** Charles I Rinerson, Roel C Rabara, Prateek Tripathi, Qingxi J Shen, Paul J Rushton

**Affiliations:** Texas A&M AgriLife Research and Extension Center, Dallas, Texas 75252 USA; Molecular and Computational Biology Section, Dana & David Dornsife College of Letters, Arts and Sciences, University of Southern California, Los Angeles, CA USA; School of Life Sciences, University of Nevada, Las Vegas, 89154 USA

**Keywords:** WRKY transcription factor, Evolution, Lateral gene transfer, Resistance protein, Charophyte

## Abstract

**Background:**

The availability of increasing numbers of sequenced genomes has necessitated a re-evaluation of the evolution of the WRKY transcription factor family. Modern day plants descended from a charophyte green alga that colonized the land between 430 and 470 million years ago. The first charophyte genome sequence from *Klebsormidium flaccidum* filled a gap in the available genome sequences in the plant kingdom between unicellular green algae that typically have 1-3 WRKY genes and mosses that contain 30-40. WRKY genes have been previously found in non-plant species but their occurrence has been difficult to explain.

**Results:**

Only two WRKY genes are present in the *Klebsormidium flaccidum* genome and the presence of a Group IIb gene was unexpected because it had previously been thought that Group IIb WRKY genes first appeared in mosses. We found WRKY transcription factor genes outside of the plant lineage in some diplomonads, social amoebae, fungi *incertae sedis*, and amoebozoa. This patchy distribution suggests that lateral gene transfer is responsible. These lateral gene transfer events appear to pre-date the formation of the WRKY groups in flowering plants. Flowering plants contain proteins with domains typical for both resistance (R) proteins and WRKY transcription factors. R protein-WRKY genes have evolved numerous times in flowering plants, each type being restricted to specific flowering plant lineages. These chimeric proteins contain not only novel combinations of protein domains but also novel combinations and numbers of WRKY domains. Once formed, R protein WRKY genes may combine different components of signalling pathways that may either create new diversity in signalling or accelerate signalling by short circuiting signalling pathways.

**Conclusions:**

We propose that the evolution of WRKY transcription factors includes early lateral gene transfers to non-plant organisms and the occurrence of algal WRKY genes that have no counterparts in flowering plants. We propose two alternative hypotheses of WRKY gene evolution: The “Group I Hypothesis” sees all WRKY genes evolving from Group I C-terminal WRKY domains. The alternative “IIa + b Separate Hypothesis” sees Groups IIa and IIb evolving directly from a single domain algal gene separate from the Group I-derived lineage.

**Electronic supplementary material:**

The online version of this article (doi:10.1186/s12870-015-0456-y) contains supplementary material, which is available to authorized users.

## Background

Over twenty years ago research began into an unknown group of DNA-binding proteins and during this time we have learned much about WRKY transcription factors. Based on the first amino acid sequences, several suggestions were made that subsequent research has shown to be correct. For example, the conserved cysteines and histidines in the WRKY domain do indeed form a novel zinc finger-like motif and the WRKY amino acid sequence binds directly its cognate *cis*-acting element, the W box (TTGACC/T) DNA binding site. As soon as the WRKY domain was characterized, it was suggested that it contained a novel zinc finger structure, even though the spacing of zinc chelating amino acids was unusual. The first evidence to support a zinc finger structure came from studies with 2-phenanthroline that chelates zinc ions. Addition of 2-phenenthroline to gel retardation assays using WRKY proteins resulted in a loss of binding to the W box target sequence [1]. The other main suggestion was that the WRKY signature amino acid sequence at the N-terminus of the WRKY domain binds directly to the W box sequence in the DNA of target promoters. This was shown to be correct by publication of the solution structure of the C-terminal WRKY domain of the Arabidopsis WRKY4 protein in the absence of binding to a W box. The WRKY domain was found to form a four-stranded β-sheet [2]. Soon afterwards, a crystal structure of the C-terminal WRKY domain of the Arabidopsis WRKY1 protein was also reported. This revealed a similar solution structure except that the WRKY domain may contain an additional β-strand at the N-terminus of the domain [3]. An important breakthrough was recently reported with the first structural determination of the WRKY domain bound to its W Box *cis*-acting element [4]. This revealed that part of a four-stranded β-sheet enters the major groove of DNA in an atypical mode that was called a β-wedge. This sheet is almost perpendicular to the DNA helical axis. As initially predicted, amino acids in the conserved WRKYGQK signature motif contact the W Box DNA bases mainly through extensive apolar contacts with thymine methyl groups [4]. These structural data provide the molecular basis to explain the previously noted conservation of both the WRKY signature sequence at the N-terminus of the WRKY domain and the W Box DNA sequence [5].

There has been interest in the evolution of the WRKY gene family as it promises to yield insights into how biotic and abiotic stress responses and signalling evolved as plants went from single cellular aquatic algae to multicellular flowering plants. The first work defined the seven major groups of WRKY genes found in flowering plants (Groups I, IIa, IIb, IIc, IId, IIe, and III) [5]. This classification was only partly based on phylogenetic analyses but has proven over time to be an accurate representation of the major groups of WRKY genes in flowering plants [5,6]. In 2005, Zhang and Wang used the availability of an increasing number of plant genome sequences to propose a hypothesis of the evolution of WRKY genes in plants [7]. They hypothesized that a proto-WRKY gene with a single WRKY domain underwent domain duplication to produce Group I WRKY genes. Subsequent loss of the N-terminal WRKY domain led to Group IIc genes from which all other WRKY genes evolved, Group III genes being the last [7]. Since this paper, the first genome sequences of a moss [8] and a spike moss [9] have been published and with this extra data it became clear that Group III genes were not the last to evolve but rather Group IIa genes [6]. Babu et al. looked for WRKY-like genes outside of the plant kingdom and showed that WRKY domains share a similar zinc finger domain and four strand fold with GCM1 and FLYWCH domains and suggested that they may be derived from a BED finger and ultimately a C2H2 zinc finger domain [10]. This appears at least partly true. The zinc finger structures of these proteins do appear to have some similarities at the primary amino acid level, suggesting that they are related. However, there appear to be no similarities in the WRKY signature portion of the domains. It is possible that the zinc finger portions of WRKY, GCM1 and FLYWCH proteins do share a common ancestor but any common structural features other than the zinc finger share no similarities at the primary amino acid sequence level. The most recent work on the evolution of the WRKY gene family, again proposed an ancestral Group I WRKY gene, Group IIa evolving from a Group I gene, Group IId evolving from Group IIa, and Group III genes being evolutionarily the youngest [11]. However, several lines of evidence from sequenced genomes show that this cannot be the case. Firstly, Group IIa WRKY genes were the last to evolve as they are the only group absent from the spike moss *Selaginella moellendorffii* [9]. This means that Group IId genes could not have evolved from Group IIa genes because Group IId genes predate Group IIa genes. Similarly, Group III-like genes are present in the moss *Physcomitrella patens* [8] and Group III genes are present in *S. moellendorffi*. Group III genes therefore predate both Group IIa genes and Group IIe genes and cannot therefore be the youngest group.

The many diverse species of modern day plants all descended from a single charophyte green alga that colonized the land between 430 and 470 million years ago [12]. The recent availability of the first genome sequence from a member of the Charophyta (the filamentous terrestrial alga *Klebsormidium flaccidum*) [13] fills in a gap in the evolutionary history of WRKY genes associated with the colonization of land by plants and reveals some unexpected new insights into WRKY evolution.

Our phylogenetic and comparative genomic studies show here that there has indeed been a lineage-specific expansion of WRKY transcription factors in plants but they are not found exclusively in plants. WRKY transcription factors most likely evolved very early in the green lineage and then a number of lateral gene transfer events have occurred in diplomonads, social amoebae, fungi *incertae sedis*, and amoebozoa. These non-plant WRKY genes do not belong to any of the seven groups of WRKY genes found in flowering plants suggesting that these lateral gene transfers are ancient and may provide insights into the early ancestral single domain WRKY genes. Based on our phylogenetic analyses and genomic searches we propose that there are four major WRKY transcription factor lineages in flowering plants, Groups I + IIc, Groups IIa + IIb, Groups IId + IIe, and Group III. Group I WRKY proteins have two WRKY domains, whereas Group II proteins have a single domain, and Group III proteins have a single domain with a C-C-H-C zinc finger structure rather than C-C-H-H. We propose two alternative hypotheses of WRKY gene evolution: The “Group I Hypothesis” sees all WRKY genes evolving from Group I C-terminal domains with IIb genes evolving before the appearance of the conserved PR intron. The alternative “IIa + b separate Hypothesis” sees Groups IIa and IIb with their hallmark VQR intron evolving directly from a single domain ancestral algal WRKY gene separate from the other Group I-derived lineage. We also show that one other type of WRKY gene has evolved in flowering plants and these proteins contain domains typical for both resistance (R) proteins and WRKY transcription factors. We have classified these R protein-WRKY genes into eight groups (RW1-RW8). R protein-WRKY genes are not present in all plant genomes but have evolved numerous times in flowering plants. Each type of R protein-WRKY gene is restricted to specific flowering plant lineages. These chimeric proteins contain not only novel combinations of protein domains but also novel combinations and numbers of WRKY domains.

## Results and discussion

### Distribution of WRKY genes in the tree of life

**Figure 1:**
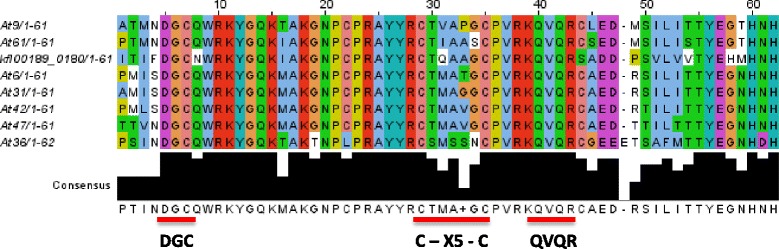
**A group IIb WRKY transcription factor from** ***Klebsormidium flaccidum*** **.** ClustalW2 multiple sequence alignment and consensus sequence of WRKY domains from Arabidopsis Group IIb genes and the IIb gene (*kfl00189*) from *Klebsormidium flaccidum*. The consensus amino acid sequence is shown and also the number of amino acids in the WRKY domains. Amino acid sequence motifs found in Group IIb WRKY transcription factors are shown and underlined in red.

**Figure 2:**
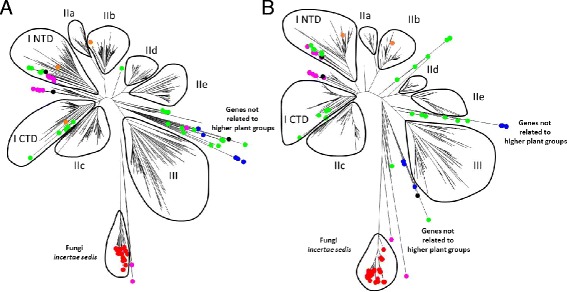
**The WRKY gene family. A**. Neighbor joining phylogenetic tree derived from a MUSCLE alignment of WRKY domains from the complete WRKY gene families from the following species: *Arabidopsis thaliana, Glycine max, Brachypodium distachyon, Selaginella moellendorffii, Physcomitrella patens, Chlamydomonas reinhardtii, Chlorella variabilis, Coccomyxa subellipsoidea, Micromonas pusilla, Ostreococcus lucimarinus, Ostreococcus tauri, Volvox carteri, Klebsormidium flaccidum, Bathycoccus prasinos, Dictyostelium discoideum, Polysphondylium pallidum, Dictyostelium fasciculatum, Fonticula alba, Acanthamoeba castellanii, Giardia lamblia, Giardia intestinalis, Dictyostelium purpureum, Auxenochlorella protothecoides, Spironucleus salmonicida, Mucor circinelloides, Rhizopus delemar, Absidia idahoensis, Lichtheimia corymbifera, Rhizophagus irregularis*, and *Mortierella verticillata*. Fungal genes are marked with a red dot, unicellular green algae green, diplomonads blue, amoebozoa black, social amoebae purple, and *Klebsormidium flaccidum* orange. The higher plant WRKY groups are marked I-III. I NTD and I CTD denote the N-terminal and C-terminal domains from Group I proteins. The tree was produced using MEGA 6. **B**. Maximum likelihood phylogenetic tree using the same MUSCLE alignment.

We searched available genome sequences for the presence of WRKY genes using PSI-BLAST and blastp searches. WRKY domains from all seven flowering plant groups were employed as well as WRKY domains from unicellular green algae. In addition, as WRKY genes were found outside of the plant kingdom, WRKY domains from the encoded proteins were also included in the searches. We produced a data set that contained all recognizable WRKY domains (manually curated with all sequences containing both a WRKY signature amino acid sequence and at least part of a zinc finger) from the following species: *Arabidopsis thaliana*, *Glycine max*, *Brachypodium distachyon*, *S. moellendorffii*, *P. patens, Chlamydomonas reinhardtii, Chlorella variabilis*, *Coccomyxa subellipsoidea*, *Micromonas pusilla*, *Ostreococcus lucimarinus*, *Ostreococcus tauri*, *Volvox carteri*, *K. flaccidum, Bathycoccus prasinos, Dictyostelium discoideum, Polysphondylium pallidum, Dictyostelium fasciculatum, Fonticula alba, Acanthamoeba castellanii, Giardia lamblia, Giardia intestinalis, Dictyostelium purpureum, Auxenochlorella protothecoides, Spironucleus salmonicida, Mucor circinelloides, Rhizopus delemar, Absidia idahoensis, Lichtheimia corymbifera, Rhizophagus irregularis,* and *Mortierella verticillata* (Additional file 1: Table S1). The Expect (E) threshold for searches was set to a high value (typically 10) so that all potential WRKY genes were found. False positives were later discarded. We set a low E-value threshold to make sure that we did not miss any variant WRKY domain sequences. All sequences were then manually curated and those that did not contain a WRKY domain-like sequence (WRKY signature amino acid sequence or the zinc finger) were discarded. Incomplete sequences (WRKY or zinc finger alone) were not discarded as they may be pseudogenes, incomplete assemblies, sequencing errors or mispredictions but these were normally not included in phylogenetic analyses. During the course of this project, the first genome sequence of a charophyte, the terrestrial filamentous green alga *K. flaccidum* was published [13]. Searches of the *K. flaccidum* genome (http://www.plantmorphogenesis.bio.titech.ac.jp/~algae_genome_project/klebsormidium/klebsormidium_blast.html) revealed that it contains just two WRKY genes. The first is a Group I gene (*kfl00096*) that contains two WRKY domains similar to the single *C. reinhardtii* gene. Unexpectedly, the second gene (*kfl00189*) is a Group IIb gene (Figure 1). Phylogenetic analyses also show that *kfl00189* clusters with other Group IIb WRKY genes (Figure 2). The presence of a Group IIb gene early in the evolution of plants, as they first colonized the land, was unexpected and not predicted by previous hypotheses about the evolution of WRKY genes [7,10,11]. This was the first observation that re-writes the accepted evolution of WRKY transcription factors.

To gain further insights into the evolution of WRKY transcription factors, we used our extensive data set of WRKY domains for phylogenetic analyses. A MUSCLE alignment [14] of the WRKY domains was produced in MEGA6 [15] (Additional file 2). Inspection of the alignment and comparisons to similar alignments produced using CLUSTALW [16] showed that the MUSCLE alignment was better at correctly aligning the zinc coordinating amino acids and fewer manual adjustments were required than with CLUSTALW results (data not shown). CLUSTALW yielded the lowest accuracy for full-length sequences in almost all test cases compared to eight other popular multiple sequence alignment programs [17] and the choice of MUSCLE to create sequence alignments instead of CLUSTALW [11] or CLUSTALX [7] is liable to result in more robust phylogenetic analyses.

Figure 2 shows both Neighbor Joining and Maximum Likelihood trees of the data set of WRKY domains. Other phylogenetic trees such as Minimum Evolution and Maximum Parsimony produced similar results. All seven flowering plant WRKY groups are present as separate clades together with several other groups that appear not to be present in flowering plants. These additional groups vary in their positions in the NJ and ML trees, probably because these proteins are not members of any of the higher plant groups. One of these non-flowering plant groups contains all the WRKY genes from fungi *incertae sedis*. These WRKY genes are the most divergent of WRKY transcription factors. The other groups consist of WRKY genes from unicellular green algae, diplomonads, social amoebae, and amoebozoa. These observations establish that some WRKY genes are found outside of the plant kingdom and that apart from some Group I-like genes in social amoebae, and amoebozoa, these non-plant genes are not representatives of any of the seven flowering plant WRKY groups.

**Figure 3:**
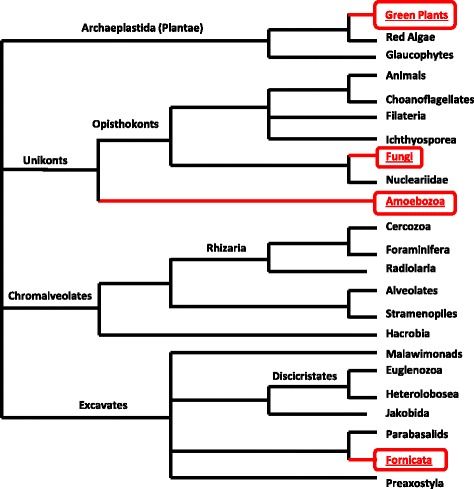
**The distribution of WRKY genes in the tree of life.** Red boxed names indicate the presence of WRKY genes.

Interestingly, although WRKY genes appear present in some diplomonads, social amoebae and other amoebozoa, and fungi *incertae sedis*, they are absent in other non-plant species. This is an unusual distribution (Figure 3). How, for instance, can there be WRKY genes in the distantly related Fornicata but not in red algae? So how can we explain this patchy phyletic distribution of WRKY genes (Figure 3)? Such a patchy distribution is a feature of lateral gene transfer [18]. It cannot easily be explained by multiple losses of WRKY genes in multiple independent lineages. Other features of lateral gene transfer include finding similar genes shared amongst unrelated species that share a specific niche/geographical location [19]. This would appear to indeed be the case with the diplomonads, social amoebae, fungi *incertae sedis*, and amoebozoa that contain WRKY genes. It is striking that almost all of the organisms outside of the plant kingdom that contain WRKY genes can be found in one of two ecological niches. Either they live in the soil in proximity to plant roots and/or they are parasites of humans/animals often in the gut. Both niches would put them close to plant material (alive or rotting in the soil or being digested in the digestive system) and transfer in the gut would suggest a later rather than earlier gene transfer. Further support for this hypothesis comes from studies of the non-plant organisms themselves. Diplomonads are known to have had frequent lateral gene transfer events [20] and lateral gene transfer is a significant evolutionary mechanism among diplomonads in particular and protists in general [20]. Studies of the genome of the amoebozoa *A. castellanii* highlighted extensive lateral gene transfer [21]. Plant-fungi lateral gene transfers appear to be both rare and ancient [22], but at least four examples have been described, suggesting that the ancient transfer of a WRKY gene from a unicellular alga to an early fungus is indeed possible.

### Non-plant WRKY genes

Fungi such as the fungi *incertae sedis* are ancient, probably over 1,000 million years old [23], and have been suggested as playing an important role in the evolution of land plants [24]. *Rhizopus microsporus* is a widely distributed soil fungus that can cause mucormycosis in immunocompromised humans and seedling blight in rice [25]. *Fungi incertae sedis* species such as *R. irregularis* (formerly *Glomus intraradices*) are mycorrhizal fungi and actually penetrate plant cells [26]. It is therefore possible that an ancient WRKY gene was transferred from a plant cell early during the evolution of land plants and that this gene has given rise to the fungal type of WRKY gene. Fossil evidence shows arbuscular mycorrhizal symbiosis to be at least as old as the earliest land plants (470–480 million years ago) and to predate plant roots [27]. It is likely that colonization of the land by plants was therefore dependent on fungal provision of inorganic nutrients and water [27]. It is possible that these first terrestrial symbioses with fungal cells led to an early lateral gene transfer of a WRKY gene to a non-plant host.

**Figure 4:**
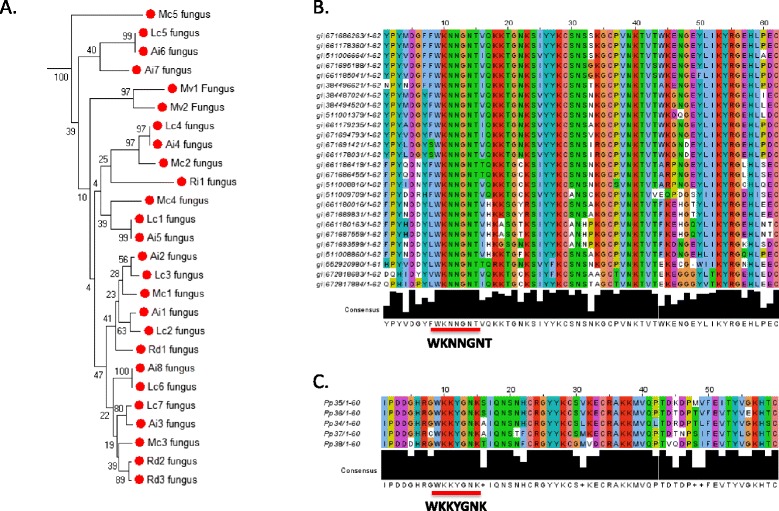
**Fungal and moss Group III WRKY proteins. A**. Part of the neighbor joining phylogenetic tree shown in Figure 2 derived from a MUSCLE alignment of WRKY domains. The WRKY domains from *Mucor circinelloides, Rhizopus delemar, Absidia idahoensis, Lichtheimia corymbifera, Rhizophagus irregularis*, and *Mortierella verticillata* are indicated by red dots and form a distinct clade. Numbers indicate bootstrap values from 1,000 replicates. **B**. ClustalW2 multiple sequence alignment and consensus sequence of WRKY domains from fungi *incertae sedis*. The conserved WKNNGNT amino acid sequence is shown **C**. ClustalW multiple sequence alignment and consensus sequence of WRKY domains from *Physcomitrella patens* Group III proteins (PpWRKY35-38). The conserved WKKYGNK amino acid sequence is shown.

Inspection of the fungal WRKY genes reveals that they are among the most divergent compared to higher plant WRKY genes (Figure 2). The WRKY signature amino acid sequence is present as a conserved WKNNGNT rather than WRKYGQK (Figure 4). In addition, the zinc finger motif is C-X6-C-H-X3-C. This spacing of C- and H-residues is unique among WRKY proteins. Fungal WRKY TFs also contain only one WRKY domain. Until now, the ancestral form of WRKY proteins has been suggested to be similar to the Group I WRKY transcription factors with two domains (N- and C-terminal) but it has been clear that a proto-WRKY with a single domain was likely present before domain duplication occurred [7]. As plant-fungi lateral gene transfers appear to be both rare and ancient [22], this suggests that the fungal-type WRKY genes may have descended from a gene closer to the original ancestral single domain-type WRKY gene than modern day Group I genes.

**Figure 5:**
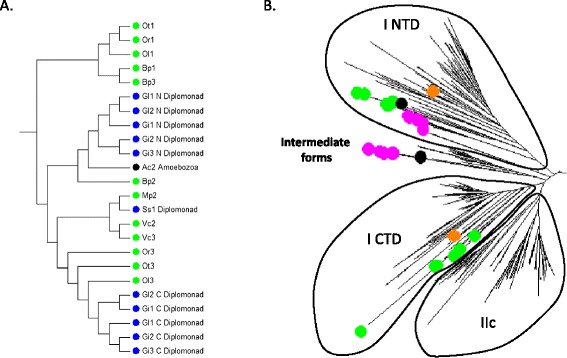
**WRKY transcription factors that are not from higher plants. A**. Bootstrap consensus tree (1,000 replicates) of a Neighbor Joining phylogenetic tree derived from a MUSCLE alignment of WRKY domains from the species described in Figure 2. Shown is a non-higher plant clade that contains algae (green), diplomonad (blue), and amoebozoa (black) WRKY transcription factors. **B**. Phylogenetic tree of Group I-like WRKY proteins from social amoebae and other amoebozoa. Groups I CTD, I NTD, IIc and an intermediate clade are shown. Unicellular green algae WRKY domains are marked with a green dot, amoebozoa black, social amoebae purple and *Klebsormidium flaccidum* in orange.

Other evidence for ancient lateral gene transfer of WRKY genes comes from the genes present in diplomonad species (*G. lamblia*, *G. intestinalis*, and *Spironucleus salmonicida*) (Figures 2 and 3). Diplomonads are free-living flagellates that are common in stagnant fresh water, but most are commensal in the intestines of animals. Some are parasitic and cause disease. They include *G. lamblia*, which causes giardiasis in humans. Diplomonad WRKY genes are not members of any of the seven flowering plant groups and are found in a distinct clade with several unicellular green algae WRKY genes (Figure 5). Even though the proteins contain two WRKY domains, they are not members of Group I. Interestingly, the C-terminal WRKY domain is characterized by the amino acid sequence WKKYGHK rather than the more common WRKYGQK. Taken together, this suggests that the diplomonads obtained a unicellular green alga type WRKY gene and that this group of WRKY genes is not represented in modern day flowering plants.

**Figure 6:**
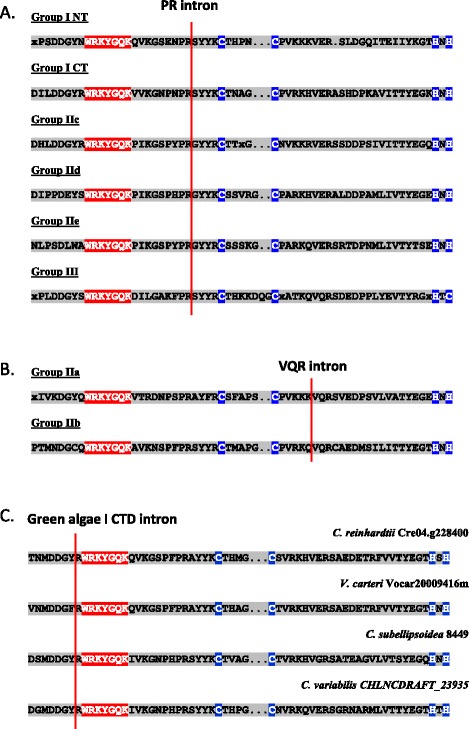
**Consensus positions of the PR, VQR, and algal I CTD introns. A**. The consensus amino acid sequences of WRKY domains from Arabidopsis Groups I, IIc, IId, IIe, and III, derived by ClustalW together with the position of the conserved PR intron. **B**. The consensus amino acid sequences of WRKY domains from Arabidopsis Groups IIa and IIb together with the position of the conserved VQR intron. **C**. The carboxy terminal domains from four algal Group I WRKY proteins together with the position of the conserved intron. The species and name of each gene are shown.

The third group of non-plant organisms that contains WRKY genes in their genomes includes some social amoebae and other amoebozoa (Figure 2). Most of the amoebozoa genes are Group I-like and contain two WRKY domains. Figure 5 shows that the C-terminal domains of these Group I proteins do not fall within the same clade as the C-terminal domains from flowering plant proteins but rather form an early branch from the C-terminal domain clade. These observations lead us to propose that a duplication event of the N-terminal WRKY domain occurred early in WRKY gene evolution and the second N-terminal domain began to evolve to become the C-terminal in unicellular green algae. Before the C-terminal WRKY domain had evolved into the higher plant form, a lateral gene transfer event occurred that resulted in an amoebozoa species containing an intermediate Group I form of WRKY gene with the C-terminal WRKY domain sharing some N-terminal features. We have found one other line of evidence that supports this suggestion. The C-terminal WRKY domain of Group I WRKY genes from multicellular plants contains the conserved PR intron that separates the WRKY part of the WRKY domain from the zinc finger part [5]. Unicellular green algal Group I genes from *C. reinhardtii*, and *V. carteri*, however, lack this conserved intron and the social amoebae Group I WRKY genes also lack this intron suggesting they are green alga-like (Figure 6).

### WRKY genes in unicellular green algae

The WRKY genes in unicellular green algae fall into three groups based on phylogenetic analyses (Figure 2). One group corresponds to the Group I genes found in flowering plants. These genes have been postulated to be ancestral to all higher plant WRKY genes, largely because the only WRKY gene present in the unicellular green alga *C. reinhardtii* is of this type. The situation in *C. reinhardtii* may, however, be a little misleading as other unicellular green algae, such as *M. pusilla*, *O. lucimarinus*, and *O. tauri* have more than a single WRKY gene but these do not include genes that are members of Group I.

We therefore propose that Group I WRKY genes may not be the universal ancestor of WRKY genes in higher plants and that other groups of WRKY genes may have evolved directly from ancient single domain WRKY gene(s). The other groups of WRKY genes in unicellular green algae might possibly have been the direct ancestors of Groups IIa and IIb in flowering plants (see below) and certainly seem to have been ancestral to some WRKY genes found outside of the plant kingdom. Consistent with this suggestion, the diplomonad WRKY genes cluster with a group of unicellular green alga WRKY genes that have a single domain and are not part of the Group I clade (Figure 5), suggesting that it was lateral gene transfer from this class of algal genes that led to diplomonad genes. This single domain type of algal WRKY gene does not appear to be represented in higher plant genomes and this suggests that these WRKY genes have no counterparts in higher plants. This is also consistent with their apparent loss in *C. reinhardtii*. The presence of these single domain WRKY genes in unicellular green algae and similar two domain versions in diplomonads suggests that Group I genes were not the only early WRKY genes that could have given rise to WRKY groups in higher plants or non-plant organisms.

### WRKY genes in multicellular green algae

Until recently, there was a large gap in the available genome sequences in the plant kingdom between unicellular green algae such as *C. reinhardtii* that typically have 1–3 WRKY genes and mosses such as *P. patens* that have 30–40 genes. This situation changed with the publication of the *K. flaccidum* genome sequence [13]. We have searched the available *K. flaccidum* genome sequence and it contains just two WRKY genes (Figures 1 and 2). The first is a Group I gene (kfl00096) that contains two WRKY domains similar to the single *C. reinhardtii* gene. Unexpectedly, the second gene (*kfl00189*) is a Group IIb gene. Phylogenetic analyses show that *kfl00189* clusters with other Group IIb WRKY genes (Figure 2). The amino acid sequence of the *kfl00189* WRKY domain also has hallmarks of Group IIb proteins from flowering plants such as the C-X5-C spacing in the zinc finger motif, the sequence QVQR in the middle of the finger, and the sequence DGCx immediately before the WRKY amino acid signature (Figure 1). All of these primary amino acid sequences are features of Group IIb WRKY proteins [5]. Strikingly, *kfl00189* also contains the conserved QVQR type intron that flowering plant Group IIb and IIa genes possess rather than the PR type intron shared by all other flowering plant WRKY genes [5].

These observations necessitate a re-evaluation of the current view of WRKY gene evolution. Previously, it had been assumed that Group IIb genes evolved from Group IIc-like genes later in the evolution of plants [7]. Now it is clear that only Group I genes predate them. However, the new information that we provide here showing an early evolution of Group IIb genes poses a new question. Did the Group IIb genes evolve from a Group I gene or did they evolve independently from a single WRKY domain-containing unicellular green alga gene? These are the two types of WRKY gene that were present before the colonization of land and multicellularity and so IIb genes must have evolved from one type or the other. We have called these two different possibilities the “IIa + b Separate Hypothesis” and the “Group I Hypothesis”. The “IIa + b Separate Hypothesis” suggests that Group IIa and IIb WRKY genes did not evolve from Group I genes whereas the “Group I Hypothesis” suggests that all WRKY genes in higher plants evolved from Group I genes. Group I WRKY genes from unicellular green algae do not appear to have the conserved PR intron that is a hallmark of most multicellular plant WRKY genes (Figure 6). Both the PR intron and the QVR intron first appear in filamentous green algae at about the time of the colonization of land. Further information is required to determine how Group IIb WRKY genes evolved in the first multicellular green algae.

### WRKY genes in mosses and spike mosses

The further evolution of WRKY genes in multicellular plants is rather clearer. The first available genome sequence of a moss (*P. patens*) showed that it contains Group I, Group IIb, Group IIc, Group IId, and Group III-like genes [8]. The newly evolved Group IIc WRKY genes appear to have evolved from Group I genes by loss of the N-terminal domain. It is likely that both Group IId and Group III genes evolved from Group IIc/Group I C-terminal domain genes based on the presence of the conserved PR intron (Figure 6).

*P. patens* has Group III-like genes that show distinct features that are not present in Group III genes from more advanced plants. For example, the single domain genes from fungi appear to be closer phylogenetically to these *P. patens* Group III genes than other WRKY genes (Figure 2). The consensus amino acid sequence of the WRKY signature from these variant moss Group III proteins is WKNNGNT, compared to WKKYGNK in fungal genes and WRKYGQK in flowering plant Group III genes (Figure 3). In filamentous green algae, there appear (based on the single available genome) to be only Group I and Group IIb genes. It is therefore likely that Group III genes evolved from Group I genes and not IIb genes because Group I and Group III share the PR intron (Figure 6). It is now clear that previous suggestions that Group III genes were the last group to evolve [7,11] are certainly incorrect as Group III genes predate Group IIa and Group IIe genes (Figure 2).

The genome sequence of a spike moss, *S. moellendorffii* [9] provided a view of the WRKY gene family in a primitive vascular plant. The approximately 40 million years of evolution that separates the mosses from *S. moellendorffii*, has seen two major changes in the WRKY gene family. Firstly, the appearance of Group IIe genes and secondly, vascular plants starting with *S. moellendorffii* have Group III WRKY genes that are similar to those in flowering plants with a similar zinc finger structure and WRKY signature amino acid sequence.

### WRKY genes in flowering plants

All of the main groups of WRKY genes that are present in flowering plants are present in *S. moellendorffii* except for Group IIa genes which were therefore the last to evolve and appear to have arisen from Group IIb genes (Figure 2). Group IIa genes are the group with the smallest number of members and appear to play many important roles in regulating stress responses (both biotic and abiotic) [6].

### The relationship with FLYWCH, GCM1, and BED proteins

**Figure 7:**
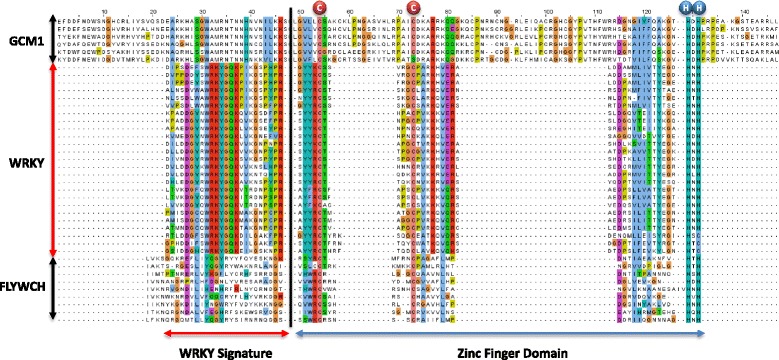
**A comparison of WRKY domains with GCM1 and FLYWCH domains.** ClustalW multiple sequence alignment of all WRKY domains from Arabidopsis, together with representative GCM1 and FLYWCH domains. The WRKY signature sequence is marked, as is the zinc finger domain with the conserved zinc binding residues.

Babu et al., (2006) suggested that WRKY domains share a similar zinc finger domain and four strand fold with GCM1 and FLYWCH domains and that they may be derived from a BED finger and ultimately a C2H2 zinc finger domain [10]. We investigated this possibility but found FLYWCH proteins are “too divergent to be aligned” in MEGA4 using CLUSTALW with our data set of WRKY domains. The same was true with GCM1-like sequences. Inspection of the domains shows that the zinc finger structure shares some amino acid similarities with the WRKY domain, even though the spacing between cysteine and histidine residues varies greatly, but that appears to be the limit of the similarity (Figure 7). The primary amino acid sequences of the N-terminal part of the domains which contain the WRKY amino acid signature sequence show no discernible similarities and could not be aligned against each other. This lack of similarity is not found with the WRKY proteins in the diplomonads, social amoebae, and fungi *incertae sedis* as they share amino acid similarities in the WRKY signature part of the domain that binds directly to DNA (Figure 4). It is possible that the zinc finger portion of the WRKY domain does share a common ancestry with FLYWCH, GCM1, MULE and BED proteins that ultimately derives from an ancestral C2H2 zinc finger motif [10]. However, our data suggest that “classical” WRKY transcription factors are too divergent to be usefully considered part of a larger family with these proteins.

### R protein-WRKYs

One of the most unusual features of the WRKY gene family in flowering plants is the existence of chimeric proteins comprising domains typical for both R proteins and WRKY transcription factors [6]. These R protein domains include toll interleukin 1 receptor (TIR), leucine-rich repeat (LRR), nucleotide-binding site (NBS), and APAF-1, R proteins, and CED-4 domain (ARC). With the sequencing of the *A. thaliana* genome, three such R protein-WRKY genes were found (*AtWRKY16, AtWRKY19,* and *AtWRKY52*) and it seemed likely that R protein-WRKY genes were a feature of most plant genomes. The majority of plant resistance (R) genes encode a class of innate immune receptors (NLRs) with nucleotide binding and leucine-rich repeat domains. R-gene evolution is thought to be facilitated by the formation of R-gene clusters, which permit sequence exchanges via recombinatorial mispairing and generate high haplotypic diversity. This pattern of evolution may also generate diversity at other loci that contribute to the R-complex [28].

**Figure 8:**
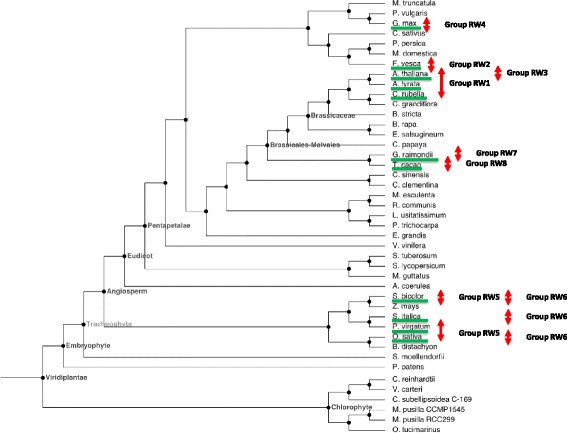
**Distribution of the eight R protein-WRKY families.** A phylogenetic tree of sequenced plant genomes is presented. The distribution of the eight R protein-WRKY families (RW1-RW8) in shown by red arrows. Based partly on phylogenetic analysis at http://phytozome.jgi.doe.gov.

With the completed sequencing of more plant genomes and extensive EST collections, we can now show here that the situation is considerably more complex. We searched available plant genome sequences and EST collections to build an atlas of R protein-WRKY genes (Figure 8 and Table 1). This has been a complicated undertaking because it was necessary to search for the presence of both NBS-LRR-like domains and at least one WRKY domain in a single transcript. Simple Blast searches often yielded results with only NBS-LRR domains or WRKY domains. Nevertheless, it was possible to establish not only that many plant genomes contain no R protein-WRKY genes but also that a considerable number of plant genomes do contain such genes. More interestingly, the combinations of domains and domain architectures found in the R protein-WRKYs are novel.

**Table 1:** **R protein-WRKY genes**

**Name**	**Species**	**Gene model and comments**	**Genomic position**
*AtRWRKY52*	*Arabidopsis thaliana*	AT5G45260, RRS1, ATWRKY52, SLH1	Chr5: 18326203 - 18332609
*AtRWRKY16*	*Arabidopsis thaliana*	AT5G45050, TTR1, ATWRKY16	Chr5: 18176914 - 18181805
*AtRWRKY19*	*Arabidopsis thaliana*	AT4G12020, ATWRKY19, MAPKKK11	Chr4: 7201656 - 7208766
*AlRWRKY1*	*Arabidopsis lyrata*	AL915586	scaffold_8: 2126189 - 2132181
*AlRWRKY2*	*Arabidopsis lyrata*	AL915663	scaffold_8: 2768079 - 2773021
*AlRWRKY3*	*Arabidopsis lyrata*	AL915648	scaffold_8: 2623905 - 2628796
*SbRWRKY1*	*Sorguhum bicolor*	SOBIC.002G104400	Chr02: 12369911 - 12381876
*SbRWRKY2*	*Sorguhum bicolor*	Sobic.008G174100	Chr08: 53517353 - 53522939
*SbRWRKY3*	*Sorguhum bicolor*	Sobic.002G168300	Chr02: 52695615 - 52704484
*CrRWRKY1*	*Capsella rubella*	CARUBV10025744M	scaffold_8:1,365,382..1,370,221
*CrRWRKY2*	*Capsella rubella*	CARUBV10025742M	scaffold_8: 1163667 - 1169265
*OsjRWRKY1*	*Oryza sativa japonica*	LOC_Os07g17230. FgenesH prediction different	Chr7:10,149,830..10,159,829
*OsiRWRKY1*	*Oryza sativa indica*	BGIOSGA035675	Chromosome 11: 21,830,082-21,837,218
*OsjRWRKY2*	*Oryza sativa japonica*	gi|108864659 Retrotransposon at 3 prime end	Chr11:27783900..27793499
*FvRWRKY1*	*Fragaria vesca*	MRNA21370	LG7: 18263740 - 18277966
*FvRWRKY2*	*Fragaria vesca*	MRNA13368	LG7: 22236162 - 22242036
*FvRWRKY3*	*Fragaria vesca*	MRNA03900ALT	LG7: 9804380 - 9813690
*FvRWRKY4*	*Fragaria vesca*	MRNA16678alt	LG6: 802048 - 808355
*AtaRWRKY1*	*Aegilops tauschii*	R7VZB5	Scaffold219315
*GmRWRKY1*	*Glycine Max*	Glyma05g29921.1	Gm05: 35364051 - 35374699
*GrRWRKY1*	*Gossypium raimondii*	Gorai.008G201000	Chr08: 48587929 - 48599304
*GrRWRKY2*	*Gossypium raimondii*	Gorai.008G200800	Chr08: 48660141 - 48668419
*TcRWRKY1*	*Theobroma cacao*	Thecc1EG006109	scaffold_2: 820964 - 828678
*TcRWRKY2*	*Theobroma cacao*	Thecc1EG006103	scaffold_2: 805474 - 814178
*TcRWRKY3*	*Theobroma cacao*	Thecc1EG006116t1	scaffold_2: 845249 - 851848
*HvRWRKY1*	*Hordeum vulgare*	MLOC_74974.5	Chr5: 483,720,745-483,727,238
*SiRWRKY1*	*Setaria italica*	Si028710m.g	scaffold_2: 26415481 - 26421105
*PvRWRKY1*	*Panicum virgatum*	Pavir.J20878	sg0.contig22731/9-CL19939Contig1
*FvRWRKY5*	*Fragaria vesca*	MRNA21370. Tandem repeat with *FvRWRKY1*	LG7: 18263740 - 18277966

Twenty nine R protein-WRKY genes are shown together with the species in which they are found, their gene models or original names, and their genomic coordinates. Their R protein-WRKY names are also shown with each gene being given a name by the insertion of the letter R between the two letter abbreviation for the species and the word WRKY.

**Figure 9:**
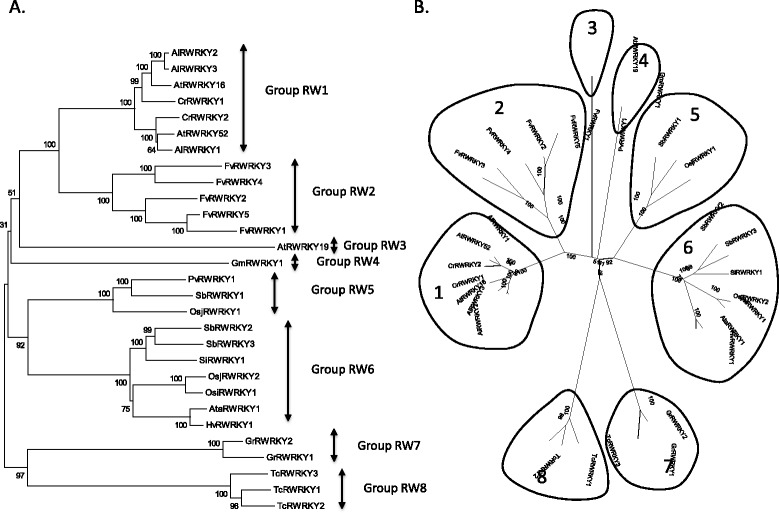
**Phylogenetic analyses of the R protein-WRKY (RW) families. A**. Neighbor Joining phylogenetic tree derived from a MUSCLE alignment of full length R protein-WRKY proteins. Numbers indicate bootstrap values from 1,000 replicates. **B**. Non-rooted version of the same tree as presented in **A**.

**Figure 10:**
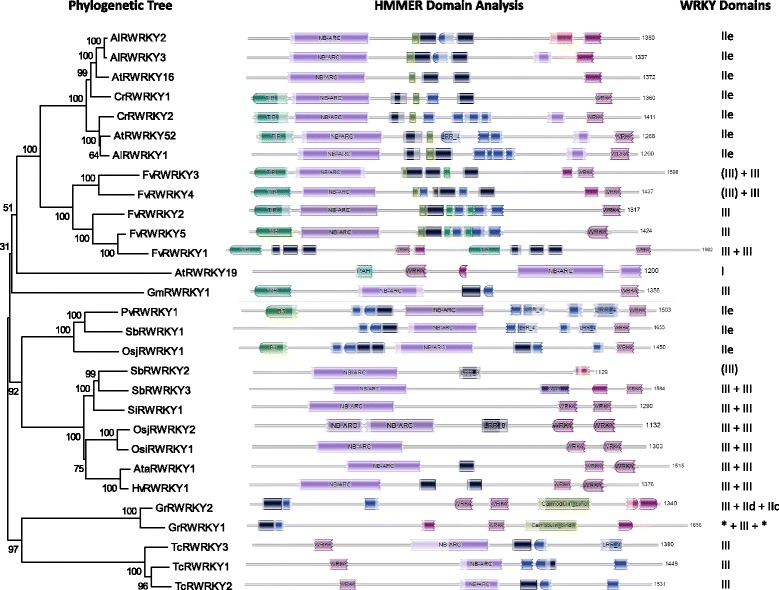
**HMMER analyses of the R protein-WRKY families.** Next to each predicted protein in the phylogenetic tree is the HMMER-derived overview of protein architecture with protein domains shown. WRKY domains are shown in reddish purple, TIR domains in green, leucine rich repeat domains in blue or black, NB-ARC domains in lilac, calmodulin-binding domains in yellowish green, NAC domains in dark purple, and B3 domains in green. The number of WRKY domains and their groups are shown to the right of the proteins.

Table 1 shows a list of R protein-WRKY genes and their amino acid sequences are presented in Additional file 3: Table S2. We have given them arbitrary group number to represent the different groupings. These genes are present in *A. thaliana*, *A. lyrata*, sorghum (*Sorghum bicolor)*, *Capsella rubella*, japonica rice (*Oryza sativa* ssp. *japonica)*, indica rice (*O. sativa* ssp. *indica)*, woodland strawberry (*Fragaria vesca)*, Tausch’s goatgrass *(Aegilops tauschii)*, soybean (*G. max)*, cotton (*Gossypium raimondii)*, cacao (*Theobroma cacao)*, barley (*Hordeum vulgare)*, foxtail millet (*Setaria italica)*, and switchgrass (*Panicum virgatum*). They appear to be lacking in other sequenced plant genomes such as corn (*Zea mays)*, purple false brome (*B. distachyon)* and barrel clover (*Medicago truncatula*). Interestingly, R protein-WRKY genes appear to have evolved on multiple independent occasions in the plant kingdom but they are confined to higher plants (Figure 8). It appears that at least eight independent genomic rearrangements resulting in NBS-LRR-WRKY (or similar) genes have occurred in the genomes of currently sequenced higher plant species (Figure 8). Representatives are present that contain WRKY domains from Group I, Group IIb, Group IId, Group IIe and Group III. Phylogenetic analyses and inspection of the architecture of the R protein-WRKYs reveals that there are at least eight types (Figure 9) and that many of these proteins have not only novel arrangements of WRKY domains, but also contain novel combinations of other protein domains (Figure 10). Here, we classify the R protein-WRKY genes into eight groups and call them R protein-WRKY1-8 (RW1-8). We are confident that more groups will be found as additional plant genomes are sequenced and annotated. The domain structures and species distributions of RW1-8 are as follows:

- Group RW1: TIR-NB ARC-LRR-WRKY (IIe). Found in Capsella and Arabidopsis.
- Group RW2: TIR-NB ARC-LRR-WRKY (III)-[WRKY (III)]. May have one or two Group III WRKY domains and may possibly be two groups. Found in strawberry.
- Group RW3: PAH-WRKY (I NT)-WRKY (I CT)-NB ARC. AtWRKY19 is the only member of this family. It also contains a MAP kinase kinase kinase domain at the C-terminal end of the protein. This region has very high sequence similarity to MAP kinase kinase kinases found in Arabidopsis.
- Group RW4: TIR-NB ARC-LRR-WRKY (III). Has the same domains as Group RW2 but different architecture (different positions of the domains) and does not cluster with the Group RW2 proteins. Found in soybean.
- Group RW5: [B3]-LRR-NB ARC-LRR-WRKY (IIe). Some, but not all, have a B3 DNA-binding domain. Found in the monocots rice, sorghum, and switchgrass.
- Group RW6: NB ARC-LRR-WRKY (III)-WRKY (III). These are found in the monocots sorghum, barley, rice, foxtail millet, and Tausch’s goatgrass. One of the proteins has only one WRKY domain and another has an additional NAC DNA-binding domain.
- Group RW7: LRR-WRKY (III)-WRKY (IId)-Calmodulin binding domain-WRKY (IIc). The two members of this group are found in *G. raimondii* (a possible progenitor species of tetraploid cotton). The WRKY domains from GrRWRKY1 are truncated and difficult to classify.
- Group RW8: WRKY (III)-NB ARC-LRR. Found in cacao.

It is clear that these genomic rearrangements are associated with specific plant lineages and appear therefore to be relatively recent events. For example, Groups RW2 and RW4 are found in the Fabidae, and RW5 and RW6 in the grasses (Figure 8). Other groups such as RW2, RW7, and RW8 have only been found in a single species and even considering the limited availability of plant genome sequences, it is likely that they are present in only a small number of related species. This suggests that the formation of many of these R protein-WRKY genes are recent events and this is consistent with information showing that many R-genes are fast-evolving and characterized by chimeric structures resulting from frequent sequence exchanges among group members [29].

Sequence exchange between R-gene paralogues is considered to be the dominant mechanism for generating variations of type I resistance genes [30]. In addition, it has been known for some time that novel disease resistance specificities result from sequence exchange between tandemly repeated genes [31]. It may be significant that many of the R protein-WRKY genes contain one or more Group III WRKY domains because we have previously shown that tandem repeats of Group III WRKY genes exist in species such as *B distachyon* [32]. It is possible that the existence of R protein-WRKY genes reflects the frequent recombination associated with some R-genes but it may also reflect a high level of recombination at some WRKY gene loci, especially tandem repeats. The strawberry NBS-LRR-WRKY genes FvRWRKY1 and FvRWRKY5 illustrate the relative instability of these genes in the genome (Figures 8, 9 and 10). FvRWRKY1 and FvRWRKY5 are found on linkage group 7 between 18245853 and 18295852. FGENESH predictions predict a single large polypeptide of 2,854 amino acids. However this predicted polypeptide contains what could be two very similar proteins with a TIR-NBS-LRR-WRKY (III) structure. The proteins are similar but not identical with blocks of similarity separated by dissimilar regions. Strikingly, an N-terminal segment of 186 amino acids from the first TIR-NBS-LRR-WRKY protein from amino acid 12 onwards is present as an identical 186 amino acids in the second TIR-NBS-LRR-WRKY protein (data not shown). Clearly, there has been a genomic rearrangement and duplication of some TIR-NBS-LRR-WRKY sequences. This illustrates that novel R protein-WRKY combinations appear to be formed through rearrangements including duplications.

We suggest that, once formed, some R protein-WRKY genes are under positive selection as they combine different components of signaling pathways that may either create new diversity in signaling or accelerate signaling by short circuiting signaling pathways. In favour of this hypothesis are the identities of other domains that have been incorporated in R protein-WRKYs. These domains do not seem to be random segments of protein coding genes but rather other signaling components such as B3 and NAC DNA-binding domains and calmodulin-binding domains (Figure 10).

It has also been observed that many transposable elements are found at R-gene loci, including retrotransposons, transposons, and miniature inverted transposable elements [29]. This may provide one mechanism by which R-gene loci are rearranged. We found transposable elements next to at least one of the 29 R protein-WRKY genes, *OsjRWRKY2* (data not shown), and transposable elements may therefore play a role in the creation of some R protein-WRKY genes.

It is possible that a small number of the predicted R protein-WRKY genes do not actually form chimeric proteins that contain all of the domains predicted by gene prediction programs such as FGENESH [33] and Hidden Markov Models (HMM). Further research will be required to determine the exact protein architecture produced from each individual gene and also whether there may be instances of alternate splicing. However, it is clear from studies of the Arabidopsis NBS-LRR-WRKY genes that these genes do indeed encode chimeric proteins and that the WRKY domain is indeed functional and binds to DNA [34].

### A re-writing of WRKY transcription factor evolution

**Figure 11:**
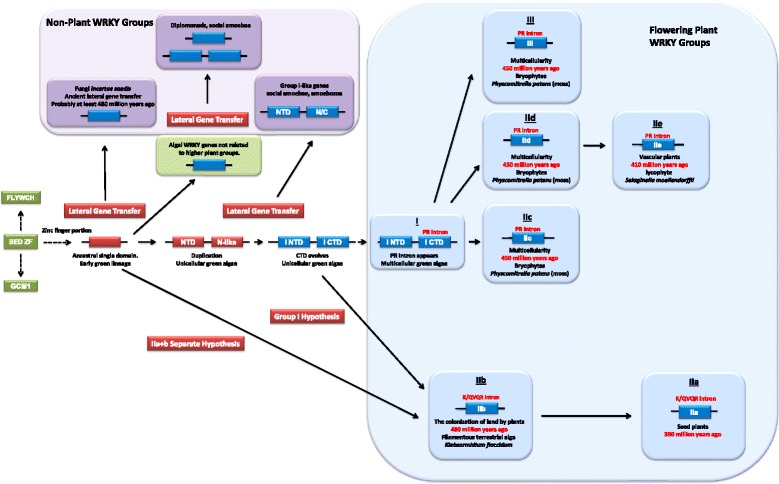
**Overview of the evolution of WRKY transcription factors.** Boxes represent WRKY domains. Red boxes indicate postulated progenitor domains. Blue boxes indicate WRKY domains from present day species. Green boxes indicate FLYWCH, GCM1 and BED zinc finger domains. Conserved introns are shown in red lettering. The four major flowering plant WRKY lineages are shown in large light blue boxes. Currently existing groups of WRKY transcription factors not found in multicellular plants are shown in large light green boxes. The direction of evolution is shown by arrows.

The availability of increasing numbers of sequenced plant genomes has necessitated a re-evaluation of the evolution of the WRKY transcription factor family. In particular, the publication of the first charophyte genome sequence from *K. flaccidum* [13] filled a large gap in the available genome sequences in the plant kingdom between unicellular green algae such as *C. reinhardtii* that typically have 1–3 WRKY genes and mosses such as *P. patens* that have 30–40 genes. We found that the *K. flaccidum* genome contains just two WRKY genes. One of these genes (as expected) was a Group I gene but the other was unexpectedly a Group IIb gene. The presence of WRKY transcription factor genes outside of the plant lineage in some diplomonads, amoebozoa and fungi *incertae sedis* sheds new light on the early evolution of WRKY genes. This leads us to suggest a new version of the evolution of WRKY transcription factors. Although there are still gaps in our knowledge, we propose the following hypothesis for how WRKY genes have evolved that best fits the currently available data (Figure 11):

Early in the green lineage, a BED finger-like C2H2 zinc finger domain evolved into a WRKY domain by the addition of a WRKY-like motif N-terminal to the zinc finger. This single domain WRKY transcription factor served as the progenitor for all other WRKY genes. There appear to have been at least four independent lateral gene transfer events to non-plants during the early evolution of the WRKY gene family. The first may have occurred as long ago as 480 million years. During the colonization of land by plants the first terrestrial symbioses and other interactions with fungal cells led to lateral gene transfer of a WRKY gene to a non-plant host. These single domain fungal type WRKY genes from fungi *incertae sedis* are ancient and reflect the single WRKY domain present in the oldest form of WRKY transcription factors from unicellular organisms. The second lateral gene transfer saw an ancestral WRKY gene with a single WRKY domain transfer to a diplomonad, and the third saw a similar gene transfer to an amoebozoa species (Figure 2). The final lateral gene transfer event was the transfer of an early Group I WRKY gene from an alga to an amoebozoa. All of these events appear to have occurred before or during the conquest of land and there may have been multiple instances of similar transfers. As more genomic sequences become available, it may be possible to identify additional lateral gene transfer events of WRKY genes to non-plant species and to more accurately date these transfer events.

In the early multicellular terrestrial algae, Group IIb genes evolved either from a single WRKY domain-containing ancestor or from a Group I gene. We propose two alternative hypotheses of Group IIb WRKY gene evolution: The “Group I Hypothesis” sees all WRKY genes evolving from Group I C-terminal domains. The alternative “IIa + b Separate Hypothesis” sees Groups IIa and IIb with their hallmark VQR intron evolving directly from a single domain ancestral algal WRKY gene separate from the other Group I-derived lineage. The conserved PR intron, that is a hallmark of most multicellular plant WRKY genes, was not a feature of the C-terminal domains of the first Group I genes from unicellular green algae but evolved in the period that saw multicellular algae colonize the land. The presence of this PR intron in Group IId, IIe and III WRKY genes supports the hypothesis that these groups evolved from the group I C-terminal domain.

We are aware that the phylogenetic trees in Figure 2 are potentially at odds with the other data because it is possible that Group I + IIc, IIa + IIb, IId + IIe and III all evolved independently from ancestral WRKY genes. However, one observation argues strongly against this. If later Groups such as IId, IIe, and III evolved after Group I but independently from other ancestral WRKY genes – then where are these other ancestral genes? They are appear to be absent from all sequenced genomes that contain the earlier Group I/IIc/IId genes. Put simply, we cannot find any ancestral WRKY genes in multicellular green algae or mosses from which later groups could independently evolve other than Group I or Group IIb.

It appears from our phylogenetic analyses and genomic searches that there are four major WRKY transcription factor lineages in flowering plants, Groups I + IIc, Groups IIa + IIb, Groups IId + IIe, and Group III. In addition, there are several other groups of WRKY genes that are found only in unicellular green algae. WRKY genes that are present in non-plant species due to ancient lateral gene transfer are either from the algal types of WRKY genes or early Group I-like genes.

During the evolution of flowering plants, one other type of WRKY genes evolved that contain domains typical for both R proteins and WRKY transcription factors. These R protein-WRKYs are not found in all plant genomes but have evolved many times and with differing domain structures (Figures 8, 9 and 10). The formation of these R protein-WRKY genes is recent with classes being restricted to specific flowering plant lineages. Once formed, R protein-WRKYs may be selected for as they combine different components of signaling pathways that may either create new diversity in signaling or accelerate signaling by short circuiting signaling pathways.

## Conclusions

Based on our phylogenetic analyses and genomic searches we propose a new hypothesis on the evolution of WRKY transcription factors that includes early lateral gene transfers to some non-plant organisms and algal WRKY genes that have no counterparts in flowering plants. There are four major WRKY transcription factor lineages in flowering plants, Groups I + IIc, Groups IIa + IIb, Groups IId + IIe, and Group III. We propose two alternative hypotheses of WRKY gene evolution: The “Group I Hypothesis” sees all WRKY genes evolving from Group I C-terminal domains with IIb genes evolving before the appearance of the conserved PR intron. The alternative “IIa + b separate Hypothesis” sees Groups IIa and IIb with their hallmark QVQR intron evolving directly from a single domain ancestral algal WRKY gene separate from the other Group I-derived lineage. Further genome sequences may help us determine which of these two alternatives is likely to best reflect the evolution of WRKY transcription factors.

### Availability of supporting data

All supporting data are included as additional files.

## Methods

### Data sets

The amino acids sequences of the complete WRKY gene families from the organisms used were taken from phytozome (http://www.phytozome.net/) [35] or NCBI (http://www.ncbi.nlm.nih.gov/). The amino acid sequences of the WRKY domains (Additional file 1: Table S1) or the complete amino acid sequences of the R protein-WRKYs (Additional file 3: Table S2) were used for phylogenetic analyses. The data set of R protein-WRKY genes was obtained using blastp, PSI-BLAST and tblastn searches at NCBI (http://www.ncbi.nlm.nih.gov/) [36,37]. Additionally, Hidden Markov models were developed to each R protein-WRKY. Genomic DNA sequences were analysed by FGENESH (http://www.softberry.com/) [33] to perform ab initio gene prediction in order to find any alternative protein predictions from those in gene models.

### Phylogenetic analyses

Alignments were constructed using MUSCLE [14] and the following parameters; Gap Penalties: Gap open −2.9, Gap Extended 0, Hydrophobicity multiplier 1.2 Memory/Iterations: Max Memory in MB 4095, Max Iterations 8; Clustering Method Iteration 1, 2 (UPGMB), Clustering Method (Other Iterations (UPGMB), Min. Diag. Length (Lambda) 24. The Alignment for Figure 2 is presented as Additional file 2.

For each Neighbor Joining tree [38], the percentage of replicate trees in which the associated taxa clustered together in the bootstrap test (1000 replicates) were determined [39]. The evolutionary distances were computed using the Poisson correction method [40] and are in the units of the number of amino acid substitutions per site. All ambiguous positions were removed for each sequence pair. Evolutionary analyses were conducted in MEGA6 [15]. All positions containing alignment gaps and missing data were eliminated in pairwise sequence. In Figure 2B, the Maximum Likelihood tree with the highest log likelihood (−20809.5522) is shown. Initial tree(s) for the heuristic search were obtained by applying the Neighbor-Joining method to a matrix of pairwise distances estimated using a JTT model. The tree is drawn to scale, with branch lengths measured in the number of substitutions per site. The analysis involved 664 amino acid sequences. All positions with less than 95% site coverage were eliminated. There were a total of 58 positions in the final dataset.

### Multiple sequence alignments and consensus sequences

Multiple sequence alignments and consensus sequences were produced using ClustalW2 (http://hmmer.janelia.org) [41] using the default settings and visualized using Jalview (www.jalview.org) [42]. The multiple sequence alignment for Figure 2 is presented in Additional file 2.

### HMMER analysis

Hidden Markov Model analyses were performed using the complete amino acid sequences on the R protein-WRKYs using the protein sequence vs profile-HMM database tool at Janelia.org (http://hmmer.janelia.org) [41] using the default settings and searching the Pfam, Gene3D, and Superfamily databases.

### Intron/exon boundary analysis

Intron/exon boundaries of individual WRKY genes were obtained from phytozome (http://www.phytozome.net/) [35]. The consensus amino acid sequences of each WRKY group from higher plants was modified from Rushton et al. [6] and obtained using all members from *Arabidopsis thaliana*.

## Additional files

Additional file 1: Table S1. All WRKY domains used for phylogenetic analysis in fasta format. Additional file 2:
**Multiple sequence alignment of all WRKY domains in Figure** 2 **.** The alignment was performed in MEGA6 using MUSCLE. Additional file 3: Table S2. All R protein-WRKYs used for phylogenetic analysis in fasta format.

## Abbreviations

B3: B3 DNA binding domain
E: Expect value
HMM: Hidden Markov Models
LRR: Leucine-rich repeat
NB(S): Nucleotide-binding site
NLR: A class of innate immune receptors with nucleotide binding and leucine-rich repeat domains
PAH: Paired amphipathic helix domain
R proteins: Resistance proteins
TIR: Toll interleukin 1 receptor
ARC: APAF-1, R proteins, and CED-4
