# Supplementary material for: The evolution of WRKY transcription factors
Source: BMC Plant Biol. 2015 Feb 27;15:66. doi: 10.1186/s12870-015-0456-y (PMC4350883; doi:10.1186/s12870-015-0456-y)
Supplement: Additional file 1: Table S1. — All WRKY domains used for phylogenetic analysis in fasta format. [file 12870_2015_456_MOESM1_ESM.doc]

>Bd1

AMISDGCQWRKYGQKMAKGNPCPRAYYRCTMAAGCPVRKQVQRCAEDRTVLITTYEGNHNH

>Bd2

EVMEDGFRWRKYGKKAVKSSPNLRNYYRCSAPGCGVKKRVERDRHDPAYVITTYHGVHNH

>Bd3

GLADDGYKWRKYGQKSIKNSPNPRSYYRCTNPRCNAKKQVERSTEEPDTLLVTYEGLHLH

>Bd4

PMISDGCQWRKYGQKMAKGNPCPRAYYRCTMATGCPVRKQVQRCAEDKTVLITTYEGSHNH

>Bd5

PIIADGCQWRKYGQKMAKGNPCPRAYYRCTMATGCPVRKQVQRCAEDRTILITTYEGTHNH

>Bd6

DIPGDEFSWRKYGQKPIKGSPYPRGYYKCSTVKGCPARKHVERATDDPAMLVVTYEGDHRH

>Bd7 N

QQLEDGYNWRKYGQKQVKGSEDPRSYYKCTHAGCSMKKKVERSLADGRVTQIVYKGAHDH

>Bd7 C

DILDDGFRWRKYGQKVVKGNPNPRSYYKCTTPGCPVRKHVERASHDARAVITTYEGKHNH

>Bd8

SPNSDGYMWRKYGQKRIMKTRFPRCYYRCSHHRERGCPATKQVQEQQHGDGADHPKTYLVIYVHEH

>Bd9

SPYKDGSQWRKYGQKNIRNRIFARYYYKCMYSHERGCRAKKQVQQQDNSSDHRPMFLITYVNEHTC

>Bd10

VPHYDGHQWRKYGQKVINNAKHPRSYYRCTYGQEQGCKATKTVQQKDDNGAGAVYEDDQVMFAVVYYGQHTC

>Bd11

NTLDDGQAWRKYGQKYIHNSKHPRAYFRCTHKYDQQCAAQRQVQRCEDDDDDKDTFRVTYIGVHTC

>Bd12

STMNDGCQWRKYGQKVAKGNPCPRAYYRCTVAPGCPVRKQVQRCQEDMSILITTYEGTHNH

>Bd13

VVPSDLWAWRKYGQKPIKGSPYPRGYYRCSSSKGCPARKQVERSRTDPNMLVITYTSDHNH

>Bd14

LVVKDGYQWRKYGQKVTRDNPSPRAYFRCAFAPSCPIKKKVQRSAENSSVLEATYEGEHNH

>Bd15

VPHYDGHQWRKYGQKNINGMQHSRSYYRCTYKERNCSATKTVQEQDHNRSSFSYGDETVKYTVVYYGHHTC

>Bd16

DHLEDGYRWRKYGQKAVKNSPYPRSYYRCTTQKCVVKKRVERSFQDPAVVITTYEGKHTH

>Bd17

LPPDDGYTWRKYGQKDILGSRYPRSYYRCTHKNYYGCEAKKKVQRLDEDPFTYEVTYCGNHSC

>Bd19

PVGDDGHSWRKYGQKEILGAKHPRGYYRCTHRKTLGCAATKQVQRSDEDPTLFDVIYHGDHTC

>Bd20

DTLVDGHVWRKYGQKEIQNSPHPRSYYRCTHKSDQGCNAKRQVQACEADPSKYAVTYYGEHTC

>Bd21

GVSSDVWAWRKYGQKPIKGSPYPRGYYRCSSSKGCPARKQVERSRADPNTFILTFTGEHNH

>Bd22

YTPDDGFSWRKYGQKDILGAKFPRGYYRCTYRTAQGCPATRQVQRSDADLAVFDVTYQGAHTC

>Bd23

DHLEDGYRWRKYGQKAVKNSPFPRSYYRCTNSKCTVKKRVERSSDDPSVVITTYEGQHCH

>Bd24

DILDDGYRWRKYGQKAVKNSSNPRSYYRCTHPTCNMKKQVQRLAKDTDIVVTTYEGTHNH

>Bd25

GTTEDGFAWRKYGQKDINGCRHPRLYYRCAYRGEGCVATRRVQRSRDEPAAYAVAYYGEHTC

>Bd26

DILDDGYRWRKYGQKAVKNNNFPRSYYRCTHQGCNVKKQVQRLSRDEGVVVTTYEGTHTH

>Bd27 N

QPGKDGYNWRKYGQKQLKDAESPRSYYKCTREACPVKKIVERSFDGCIKEITYKGRHTH

>Bd27 C

DLLDDGYRWRKYGQKVVKGNPRPRSYYKCTAENCNVRKQIERASSNPSCVLTTYTGRHSH

>Bd28

TTPEDGQSWRKYGQKFIHKSTNPRSYYRCTHKHDQGCKATKQVQKSESNPSEFVISYFGEHTC

>Bd29

VPHYDGHQWRKYGQKNINGMQHPRSYYRCTYKERSCSATKTVQKQDHNGSSFSYGDEAVNYTVMYYGNHTC

>Bd30

DHLDDGYRWRKYGQKAVKNSSYPRSYYRCTAARCGVKKQVERSQQDPATVITTYEGQHQH

>Bd31

DIPPDEYSWRKYGQKPIKGSPHPRGYYKCSSVRGCPARKHVERCVDDPSMLIVTYEGEHNH

>Bd32 N

TPAEDGYSWRKYGQKQVKHSEYPRSYYKCTHQSCQVKKKVERSHEGHVTEIIYKGTHNH

>Bd32 C

DILDDGYRWRKYGQKVVKGNPNPRSYYKCTHPGCSVRKHVERASHDLKSVITTYEGKHNH

>Bd33

DVLDDGYKWRKYGQKVVKNSLHPRSYYRCTHSNCRVKKRVERLSEDCRMVITTYEGRHTH

>Bd34

EIPADDFSWRKYGQKPIKGSPYPRGYYKCSTVRGCPARKHVERDPSEPSMLIVTYEGDHRH

>Bd35

DIPADEFSWRKYGQKPIKGSPYPRGYYKCSTVRGCPARKHVERATDDPAMLVVTYEGEHRH

>Bd36

VVPSDLWAWRKYGQKPIKGSPHPRGYYRCSSSKGCSARKQVERSRTDPNMLVITYTSEHNH

>Bd37 N

IAAEDGYNWRKYGQKQVKNSDHPRSYYKCSHPNCPVKKKVERCQDGHITEIVYKGSHNH

>Bd37 C

DILDDGYRWRKYGQKVVKGNPNPRSYYRCTHPGCSVRKHVERASNDPKSVITTYEGKHDH

>Bd38 N

NVVGDGFNWRKYGQKQVKSSDNSRSYYRCTNSSCLAKKKVEHYPDGRVIEIIYRGTHSH

>Bd38 C

GKMSDGYRWRKYGQKIVKGNPNPRSYYRCTHDGCPVRKHVEKAADDINNMVVTYEGKHNH

>Bd39

LVVKDGYQWRKYGQKVTKDNPCPRAYFRCSFAPACPVKKKVQRSAEDRTVLVATYEGEHNH

>Bd40

GPGEDGHSWRKYGQKDILGAKHPRGYYRCTHRNSQGCAATKQVQRADHDPALFDVVYHGEHTC

>Bd41

DVLDDGYKWRKYGQKVVKNSLHPRSYFRCTQSNCRVKKRVERLSTDCRMVITTYEGRHTH

>Bd42

VVPSDLWAWRKYGQKPIKGSPYPRGYYRCSSSKGCPARKQVERSRTDPNTLVITYTSEHNH

>Bd43

APYDDGHQWRKYGEKKLSNSNFPRFYYRCTYKNDMKCPATKQVQQKDTSDPPLFSVTYFNHHTC

>Bd44

TPENDGFHWRKYGEKKILNAAFPRLYYRCGYSDEHKCPAKKYVQQQDNGDPPLFMVTLINDHTC

>Bd45 N

RSSDDGYNWRKYGQKQVKGSENPRSYYKCTFPSCPTKKKVETSLEGQITEIVYKGTHNH

>Bd45 C

DILDDGYRWRKYGQKVVKGNPNPRSYYKCTTVGCPVRKHVERASQDLRAVITTYEGKHNH

>Bd46 N

RSSDDGYNWRKYGQKQMKGSENPRSYYKCSAPGCPTKKKVEQAPDGHVTEIVYKGTHNH

>Bd46 C

DILDDGYRWRKYGQKVVKGNPNPRSYYKCTMAGCPVRKHVERASQDLRAVVTTYEGKHNH

>Bd47 N

KQLEDGYKWRKYGQKQVKGSENPRSYYKCTYSNCSMKKKVERSLADGRITQIVYKGAHHH

>Bd47 C

DILDDGFRWRKYGQKVVKGNPNPRSYYKCTTVACPVRKHVERASHDNRAVITTYEGKHNH

>Bd48

PTMNDGCQWRKYGQKISKGNPCPRAYYRCTVAPSCPVRKQVQRCADDMSILITTYEGAHTH

>Bd49

DVLDDGYKWRKYGQKVVKNTHHPRSYYRCTQDKCRVKKRVERLAEDPRMVITTYEGRHVH

>Bd50

EILEDGFKWRKYGKKAVKNSPNPRNYYRCSAERCGVKKRVERDRDDPRFVVTTYDGVHNH

>Bd51

APLDDGLSWRKYGQKDILGAKYPRAYFRCTHRNTQGCQATKQVQRDAGDPLIFDVVYHGDHTC

>Bd52

EVMDDGYRWRKYGKKMVKNSPNPRNYYRCSSEGCRVKKRVERERDDARFVITTYHGVHDH

>Bd53

DHLEDGYRWRKYGQKAVKNSPYPRSYYRCTTPKCGVKKRVERSYQDPSTVITTYEGQHTH

>BD54

VLDDGYRWRKYGQKAVKNSAFPRSYYRCTHHTCNVKKQVQRLAKDTAVVVTTYEGVHNH

>Bd55

TPLEDRFSWRKYGQKDILGAKYPRAYFRCTHRHTQSCSASKQVQRTDGDPLLFDVVYHGNHTC

>Bd56

EILDDGYKWRKYGKKSVKNSPNPRNYYRCSTEGCSVKKRVERDRDDPSYVVTTYEGTHSH

>Bd57

KMPTDGYKWRKYGQKSIKNNPHPRSYYKCTSSRCSAKKHVEKSTHDPEMFTVTYEGLHLH

>Bd58

EVLDDGYKWRKYGKKSVKNSPNPRNYYRCSTEGCNVKKRVERDRDDADYVLTMYEGIHNH

>Bd59 N

RLSYDGYNWRKYGQKQVKGSEFPRSYYKCTYPTCPVKRKVETTLDGQIAEIVYNGEHNH

>Bd59 C

ESSEDAFRWRKYGQKAVNGNLFPRSYYRCSTARCNARKFVERSSDNSLVTTYEGRHNH

>Bd60

DILDDGYRWRKYGQKVVKGNPNPRSYYKCTHPGCSVRKHVERASHDLKSVITTYEGKHNH

>Bd61 N

KPADDGYNWRKYGQKVVKGSDCPRSYYKCTHPSCPVKKKVEHAEDGQISEIIYKGKHNH

>Bd61 C

DLLDDGYRWRKYGQKVVKGNPHPRSYYKCTFAGCNVRKHIERASSDPKAVITTYEGKHNH

>Bd62 N

RRTDDGYNWRKYGQTQVKGSENPRSYYKCAFPSCPAKKKVERSLDNQIIEILYKGRHNH

>Bd62 C

RSADDGYNWRKYGQTQVKGSENLRSYYKCTFPNCSTKKKVERSLANHIIEILYKGRHNH

>Bd63

DIPPDEYSWRKYGQKPIKGSPHPRGYYKCSSVRGCPARKHVERCVDEPAMLAVTYEGEHNH

>Bd64 N

KSAEDGYNWRKYGQKHVKGSENPRSYYKCTHPNCEVKKLLERAADGQITEVVYKGRHNH

>Bd64 C

DILDDGYRWRKYGQKVVKGNPNPRSYYKCTSTGCPVRKHVERASHDPKSVITTYEGKHNH

>Bd66 N

PADDGYNWRKYGQKAVKGGEYPRSYYKCTQAGCPVKKKVERSACGEITQIIYRGQHNH

>Bd66 C

DLLDDGYRWRKYGQKVVKGNPHPRSYYKCTFQGCDVKKHIERCSQDSTDVITTYEGKHSH

>Bd67 N

RPADDGYNWRKYGQKAVKGGRYPRSYYKCTLNCPVRKNVEHSEDGKIIKIIYRGQHSH

>Bd67 C

DLLDDGYRWRKYGQKVVRGNPHPRSYYKCTYQGCDVKKHIERSSQEPHAVITTYEGKHVH

>Bd68

LVVKDGYQWRKYGQKVTKDNPCPRAYFRCSFAPACPVKKKVQRSADDKALLVATYEGDHNH

>Bd69

ASEADPWAWRKYGQKTVKGSPYTRSYYRCSTAKECGARKIMELCPTDPDTLILTYTGADHNH

>Bd70

PTMNDGCQWRKYGQKISKGNPCPRAYYRCTVAAGCPVRKQVQRCAEDMSILISTYEGRHNH

>Bd71

DHLEDGYRWRKYGQKAVKNSPFPRSYYRCTNSKCTVKKRVERSSNDPSIVITTYEGQHCH

>Bd72

VMPEDGYEWKKYGQKFIKNIQRIRSYFRCRDKRCGAKKKVEWQPGDPSLRVVYEGAHQH

>Bd73

VIPSDLWAWRKYGQKPIKGSPYPRGYYRCSSSKGCMARKQVERSRSDPNMLVITYTAEHNH

>Bd74

DIPGDEFSWRKYGQKPIKGSPHPRGYYKCSSVRGCPARKHVERCVDDPAMLIVTYEGDHNH

>Bd76

DHLEDGYRWRKYGQKAVKNSPFPRSYYRCTAQKCPVKKRVERSFQDAAVVITTYEGKHTH

>Bd77

PPPPDSWAWRKYGQKPIKGSPYPRGYYRCSSSKGCPARKQVERSRTDPTVLLVTYSFDHNH

>Bd78

APHNDGHQWRKYGEKKINNCNFPRYYYRCTYKDNMNCPATKQIQQKDHSDPPLYQVTYYNEHSC

>Bd79

KNVEDGQSWRKYGQKDIQNSEHPKSYFRCTHKYDQKCAALRQVQRCDQDPESFVVTYIGQHTC

>Bd80

NTPTDEWAWRKYGQKPIKGSPFPRAYYRCSSSKGCPARKQVERSQADPAMVLVTYSYEHNH

>Bd81

GDADDLWAWRKYGQKPIKGSPYPRGYYKCSSLKACAARKLVERSPDKPEVLIVTYIADHCH

>Bd82

GVSSDLWAWRKYGQKPIKGSPYPRGYYKCSSMKGCMARKMVERSPAKPGVLVITYMAEHCH

>Bd83

RTVEDGYIWRKYGQKEILNSSHPRLYFRCSYKHDSGCPATRQVQHSDHDPSLYVITYFGHHTC

>Bd84

APYHDGFQWRKYGQKMIRGNIFPRCYYRCTYHQDHGCPASKHVEQSNSEDPPLFRVIYTNDHTC

>Bd85

TPHFDGHLWRKYGQKNIKDSAFPRLYYRCSYREDKRCLASKLVQQENCDDPPLFKVTYTYEHSC

>Bd86

VPDFDGYQWRKYGQKHIEAARYPRSYYRCTNSTDQGCPAKRTVQRNEDGADGSDGGTKAAPKYTVVYISAHTC

>At1N

KVMEDGYNWRKYGQKLVKGNEFVRSYYRCTHPNCKAKKQLERSAGGQVVDTVYFGEHDH

>At1C

DIVNDGYRWRKYGQKSVKGSPYPRSYYRCSSPGCPVKKHVERSSHDTKLLITTYEGKHDH

>At2N

APAEDGYNWRKYGQKLVKGSEYPRSYYKCTNPNCQVKKKVERSREGHITEIIYKGAHNH

>At2C

DILDDGYRWRKYGQKVVKGNPNPRSYYKCTAPGCTVRKHVERASHDLKSVITTYEGKHNH

>At3

KPADDGYNWRKYGQKQVKGSDFPRSYYKCTHPACPVKKKVERSLDGQVTEIIYKGQHNH

>At4N

KPADDGYNWRKYGQKQVKGSEFPRSYYKCTNPGCPVKKKVERSLDGQVTEIIYKGQHNH

>At4C

DLLDDGYRWRKYGQKVVKGNPYPRSYYKCTTPGCGVRKHVERAATDPKAVVTTYEGKHNH

>At6

pmisdgcqwrkygqkmakgnpcprayyrctmatgcpvrkqvqrcaedrsilittyegnhnh

>At7

DIPSDEFSWRKYGQKPIKGSPHPRGYYKCSSVRGCPARKHVERALDDAMMLIVTYEGDHNH

>At8

DHLEDGYRWRKYGQKAVKNSPYPRSYYRCTTQKCNVKKRVERSYQDPTVVITTYESQHNH

>At9

atmndgcqwrkygqktakgnpcprayyrctvapgcpvrkqvqrcledmsilittyegthnh

>At10

dnpndgyrwrkygqkvvkgnpnprsyfkctniecrvkkhvergadniklvvttydgihnh

>At11

dippdeyswrkygqkpikgsphprgyykcstfrgcparkhveralddpamlivtyegehrh

>At12

dvlddgykwrkygqkvvknslhprsyyrcthnncrvkkrverlsedcrmvittyegrhnh

>At13

DVLDDGYRWRKYGQKVVKNTQHPRSYYRCTQDKCRVKKRVERLADDPRMVITTYEGRHLH

>At14

VVPSDLWAWRKYGQKPIKGSPFPRGYYRCSSSKGCSARKQVERSRTDPNMLVITYTSEHNH

>At15

DVPPDDYSWRKYGQKPIKGSPHPRGYYKCSSVRGCPARKHVERAADDSSMLIVTYEGDHNH

>At17

DIPPDEYSWRKYGQKPIKGSPHPRGYYKCSTFRGCPARKHVERALDDSTMLIVTYEGEHRH

>At18

LTVKDGFQWRKYGQKVTRDNPSPRAYFRCSFAPSCPVKKKVQRSAEDPSLLVATYEGTHNH

>At20 N

ILADDGYNWRKYGQKHVKGSEFPRSYYKCTHPNCEVKKLFERSHDGQITDIIYKGTHDH

>At20 C

DILDDGYRWRKYGQKVVRGNPNPRSYYKCTAHGCPVRKHVERASHDPKAVITTYEGKHDH

>At21

DIPPDDYSWRKYGQKPIKGSPYPRGYYKCSSMRGCPARKHVERCLEDPAMLIVTYEAEHNH

>At22

ALNSDVWAWRKYGQKPIKGSPYPRGYYRCSTSKGCLARKQVERNRSDPKMFIVTYTAEHNH

>At23

DHLEDGYRWRKYGQKAVKNSPFPRSYYRCTTASCNVKKRVERSFRDPSTVVTTYEGQHTH

>At24

DVLDDGYRWRKYGQKSVKHNAHPRSYYRCTYHTCNVKKQVQRLAKDPNVVVTTYEGVHNH

>At25 N

RNSNDGYGWRKYGQKQVKKSENPRSYFKCTYPDCVSKKIVETASDGQITEIIYKGGHNH

>At25 C

DVLIDGFRWRKYGQKVVKGNTNPRSYYKCTFQGCGVKKQVERSAADERAVLTTYEGRHNH

>At26 N

KTSDDGYNWRKYGQKQVKGSENPRSYFKCTYPNCLTKKKVETSLVKGQMIEIVYKGSHNH

>At26 C

DILDDGYRWRKYGQKVVKGNPNPRSYYKCTFTGCFVRKHVERAFQDPKSVITTYEGKHKH

>At27

NLSSDLWAWRKYGQKPIKGSPYPRNYYRCSSSKGCLARKQVERSNLDPNFIVTYTGEHTH

>At28

DHLEDGYRWRKYGQKAVKNSPYPRSYYRCTTQKCNVKKRVERSFQDPTVVITTYEGQHNH

>At29

NLLSDAWAWRKYGQKPIKGSPYPRSYYRCSSSKGCLARKQVERNPQNPEKFTITYTNEHNH

>At30

RTLDDGFSWRKYGQKDILGAKFPRGYYRCTYRKSQGCEATKQVQRSDENQMLLEISYRGIHSC

>At31

AMISDGCQWRKYGQKMAKGNPCPRAYYRCTMAGGCPVRKQVQRCAEDRSILITTYEGNHNH

>At32 N

tpardgynwrkygqkqvkspkgsrsyyrctyteccakkiecsndsgnvveivnkglhth

>At32 C

gicgdgyrwrkygqkmvkgnphprnyyrctsagcpvrkhietaventkaviitykgvhnh

>At33 N

RKGEDGYNwrkygqkqvkgsenprsyykctfpncptkkkverslegqiteivykgshnh

>At33 C

DILDDGYRwrkygqkvvkgnpnprsyykcttigcpvrkhverashdmravittyegkhnh

>At34 N

APADDGYNWRKYGQKLVKGSEYPRSYYKCTHPNCEAKKKVERSREGHIIEIIYTGDHIH

>At34 C

DILDDGYRWRKYGQKVVKGNPNPRSYYKCTANGCTVTKHVERASDDFKSVLTTYIGKHTH

>At35

VVPSDLWAWRKYGQKPIKGSPYPRGYYRCSSSKGCSARKQVERSRTDPNMLVITYTSEHNH

>At36

PSINDGCQWRKYGQKTAKTNPLPRAYYRCSMSSNCPVRKQVQRCGEEETSAFMTTYEGNHDH

>At38

PIYYDGYLWRKYGQKSIKKSNHQRSYYRCSYNKDHNCEARKHEQKIKDNPPVYRTTYFGHHTC

>At39

DIPPDEYSWRKYGQKPIKGSPHPRGYYKCSSVRGCPARKHVERCIDETSMLIVTYEGEHNH

>At40

LVVKDGYQWRKYGQKVTRDNPSPRAYFKCACAPSCSVKKKVQRSVEDQSVLVATYEGEHNH

>At41

GPHDDIFSWRKYGQKDILGAKFPRSYYRCTFRNTQYCWATKQVQRSDGDPTIFEVTYRGTHTC

>At42

PMLSDGCQWRKYGQKMAKGNPCPRAYYRCTMAVGCPVRKQVQRCAEDRTILITTYEGNHNH

>At43

DILDDGYRWRKYGQKSVKNSLYPRSYYRCTQHMCNVKKQVQRLSKETSIVETTYEGIHNH

>At44 N

RSSVDGYNWRKYGQKQVKGSECPRSYYKCTHPKCPVKKKVERSVEGQVSEIVYQGEHNH

>At44 C

DSLEDGFRWRKYGQKVVGGNAYPRSYYRCTSANCRARKHVERASDDPRAFITTYEGKHNH

>At45

DILDDGYRWRKYGQKAVKNNPFPRSYYKCTEEGCRVKKQVQRQWGDEGVVVTTYQGVHTH

>At46

GSIDDGHCWRKYGQKEIHGSKNPRAYYRCTHRFTQDCLAVKQVQKSDTDPSLFEVKYLGNHTC

>At47

TTVNDGCQWRKYGQKMAKGNPCPRAYYRCTMAVGCPVRKQVQRCAEDTTILTTTYEGNHNH

>At48

DNLDDGYRWRKYGQKAVKNSPYPRSYYRCTTVGCGVKKRVERSSDDPSIVMTTYEGQHTH

>At49

GMCDDGYKWRKYGQKSIKNSPNPRSYYKCTNPICNAKKQVERSIDESNTYIITYEGFHFH

>At50

EVLDDGFKWRKYGKKMVKNSPHPRNYYKCSVDGCPVKKRVERDRDDPSFVITTYEGSHNH

>At51

dvmddgfkwrkygkksvknninkrnyykcssegcsvkkrverdgddaayvittyegvhnh

>At53

GPQDDVFSWRKYGQKDILGAKFPRSYYRCTHRSTQNCWATKQVQRSDGDATVFEVTYRGTHTC

>At54

KSSEDRYAWRKYGQKEILNTTFPRSYFRCTHKPTQGCKATKQVQKQDQDSEMFQITYIGYHTC

>At55

LPPDDNHTWRKYGQKEILGSRFPRAYYRCTHQKLYNCPAKKQVQRLNDDPFTFRVTYRGSHTC

>At56

DVLDDGYRWRKYGQKSVKNNAHPRSYYRCTYHTCNVKKQVQRLAKDPNVVVTTYEGVHNH

>At57

DNLEDGYRWRKYGQKAVKNSPFPRSYYRCTNSRCTVKKRVERSSDDPSIVITTYEGQHCH

>At58 N

KPADDGYNWRKYGQKPIKGCEYPRSYYKCTHVNCPVKKKVERSSDGQITQIIYKGQHDH

>At58 C

DLLDDGYRWRKYGQKVVKGNPHPRSYYKCTTPNCTVRKHVERASTDAKAVITTYEGKHNH

>At59

VALDDGYKWRKYGKKPITGSPFPRHYHKCSSPDCNVKKKIERDTNNPDYILTTYEGRHNH

>At60

LTVKDGYQWRKYGQKITRDNPSPRAYFRCSFSPSCLVKKKVQRSAEDPSFLVATYEGTHNH

>At61

PTMNDGCQWRKYGQKIAKGNPCPRAYYRCTIAASCPVRKQVQRCSEDMSILISTYEGTHNH

>At62

PIYHDGFLWRKYGQKQIKESEYQRSYYKCAYTKDQNCEAKKQVQKIQHNPPLYSTTYFGQHIC

>At63

PRLDDGFTWRKYGQKTIKTSLYQRCYYRCAYAKDQNCYATKRVQMIQDSPPVYRTTYLGQHTC

>At64

PRPDDGFTWRKYGQKTIKTSPYQRCYYRCTYAKDQNCNARKRVQMIQDNPPVYRTTYLGKHVC

>At65

TPPSDSWAWRKYGQKPIKGSPYPRGYYRCSSTKGCPARKQVERSRDDPTMILITYTSEHNH

>At66

PAHIDGFIWRKYGQKTIKTSPHQRWYYRCAYAKDQNCDATKRVQKIQDNPPVYRNTYVGQHAC

>At67

MCPNDGFTWRKYGQKTIKASAHKRCYYRCTYAKDQNCNATKRVQKIKDNPPVYRTTYLGKHVC

>At68

LHLDDGYKWRKYGQKPVKDSPFPRNYYRCTTTWCDVKKRVERSFSDPSSVITTYEGQHTH

>At69

YPPSDSWAWRKYGQKPIKGSPYPRGYYRCSSSKGCPARKQVERSRVDPSKLMITYACDHNH

>At70

TILEDAFSWRKYGQKEILNAKFPRSYFRCTHKYTQGCKATKQVQKVELEPKMFSITYIGNHTC

>At71

DHLEDGYRWRKYGQKAVKNSPYPRSYYRCTTQKCNVKKRVERSFQDPSIVITTYEGKHNH

>At72

PTMNDGCQWRKYGQKIAKGNPCPRAYYRCTVAPGCPVRKQVQRCADDMSILITTYEGTHSH

>At74

DIPPDEYSWRKYGQKPIKGSPHPRGYYKCSSVRGCPARKHVERCVEETSMLIVTYEGEHNH

>At75

DILDDGYRWRKYGQKAVKNNKFPRSYYRCTYGGCNVKKQVQRLTVDQEVVVTTYEGVHSH

>Sm1

DIMDDGYRWRKYGQKAVKNSPHPRSYYRCTNTKCPVKKRVERSSEDQGLVITTYEGIHNH

>Sm2 N

APEHDGYNWRKYGQKQVKGCDNPRSYYRCTHPDCSAKKLVERSVSGETTQIVYKGDHSH

>Sm2 C

DVLDDGYRWRKYGQKVVKGNPNPRSYYRCTNPGCPVRKHVERAADDPKAVITSYEGKHDH

>Sm3

EIPPDDYSWRKYGQKPIKGSPHPRGYYKCSSMRGCPARKHVERCLEDPSMLIVTYEGEHNH

>Sm4

VLPSDMWAWRKYGQKPIKGSPYPRGYYRCSSSKGCSARKQVERSRNDPTMLIITYTSEHNH

>Sm5

APADDGFTWRKYGQKDILNSKFPRSYYRCTHQKELGCQATKYVQKCEDEPSMYQVTYIGEHSC

>Sm6

VLPSDMWAWRKYGQKPIKGSPYPRGYYRCSSSKGCSARKQVERSRNDPTMLIITYTSEHNH

>Sm7N

RPSEDGFNWRKYGQKQVKGSEFPRSYYKCTSSGCPVKKKVERSQDGQVTEIVYKGEHNH

>Sm7C

DILDDGYRWRKYGQKIVKGNPYPRSYYKCTNVGCPVRKHVERASNDPKSVITTYEGKHNH

>Sm8

DIMDDGFRWRKYGQKAVKNSPHPRSYYRCTNSKCPVKKRVERSCEDPGIVITTYEGTHTH

>Sm9 N

RSSDDGYNWRKYGQKLVKGSENPRSYYKCTYVNCPMKKKVERSPDGQVTEIVYEGEHNH

>Sm9 C

DILDDGYRWRKYGQKVVKGNPHPRSYYKCTNLGCPVRKHVERACDDPRAVITTYEGKHNH

>Sm10

DIPQDEYSWRKYGQKPIKGSPHPRGYYKCSSLRGCPARKHVERCLDDPTMLRVTYEGEHSH

>Sm11 N

RPSEDGFNWRKYGQKQVKGSEFPRSYYKCTHPSCPVKKKVERSYDGQVTEIVYKGEHCH

>Sm11 C

DILDDGYRWRKYGQKVVKGNPHPRSYYKCSSSGCAVRKHVERASNDPKSVITTYEGKHNH

>Sm12

DIPQDEYSWRKYGQKPIKGSPHPRGYYKCSSLRGCPARKHVERCLDDPTMLRVTYEGEHSH

>Sm13 N

RSSDDGYNWRKYGQKLVKGSENPRSYYKCTYVNCPMKKKVERSPDGQVTEIVYEGEHNH

>Sm13 C

DILDDGYRWRKYGQKVVKGNPHPRSYYKCTNLGCPVRKHVERACDDPRAVITTYEGKHNH

>Sm14

DIPPDDYSWRKYGQKPIKGSPHPRGYYKCSSMRGCPARKHVERSLEDPSMLIVTYEGEHNH

>Sm15

DIMDDGFRWRKYGQKAVKNSPHPRSYYRCTNSKCPVKKRVERSCEDPGIVITTYEGTHTH

>Sm16

PTMNDGCQWRKYGQKMAKGNPCPRAYYRCTMASGCPVRKQVQRCADDTSVLVTTYEGSHNH

>Sm17

EIPPDDYSWRKYGQKPIKGSPHPRGYYKCSSMRGCPARKHVERCLEDPSMLIVTYEGEHNH

>Sm18

EIMDDGYRWRKYGQKAVKNSPYPRSYYRCTYTKCHVKKRVERSSKDSSLVITTYEGVHTH

>Sm19

LVPDDGYTWRKYGQKDILGSRHPKSYYRCTHKRESGCQAIKYVQRSDSNPSSFQITYRGEHTC

>Sm20

AQMNDGCQWRKYGQKMAKGNPCPRAYYRCTVAPGCPVRKQVQRCADDMSILITTYEGSHNH

>Sm21

EIMDDGYRWRKYGQKAVKNSPYPRSYYRCTYTKCHVKKRVERSSKDSSLVITTYEGVHTH

>Sm22

DIMDDGFRWRKYGQKAVKNSPHPRSYYRCTNSKCPVKKRVERSCEDPGIVITTYEGTHTH

>Sm23 N

APEHDGYNWRKYGQKQVKGCDNPRSYYRCTHPDCSAKKLVERSVSGETTQIVYKGDHSH

>Sm23 C

DVLDDGYRWRKYGQKVVKGNPNPRSYYRCTNPGCPVRKHVERAADDPKAVITSYEGKHDH

>Sm24

PTMNDGCQWRKYGQKMAKGNPCPRAYYRCTMSPGCPVRKQVQRCAEDTSILVTTYEGTHNH

>Sm25

LVPDDGYTWRKYGQKDILGSRHPKSYYRCTHKRESGCPAIKYVQRSDSNPSSFQITYRGEHTC

>Sm26

PTMNDGCQWRKYGQKMAKGNPCPRAYYRCTMASGCPVRKQVQRCADDTSVLVTTYEGSHNH

>Sm27

DIPPDDYSWRKYGQKPIKGSPHPRGYYKCSSMRGCPARKHVERSLEDPSMLIVTYEGEHNH

>Sm28

APADDGFTWRKYGQKDILNSKFPRSYYRCTHQKELGCQATKYVQKCEDEPSMYQVTYIGEHSC

>Sm29

PTMNDGCQWRKYGQKMAKGNPCPRAYYRCTMSPGCPVRKQVQRCAEDTSILVTTYEGTHNH

>Sm30 N

RPSEDGFNWRKYGQKQVKGSEFPRSYYKCTHPSCPVKKKVERSYDGQVTEIVYKGEHCH

>Sm30 C

DILDDGYRWRKYGQKVVKGNPHPRSYYKCSSSGCAVRKHVERASNDPKSVITTYEGKHNH

>Sm31 N

RPSEDGFNWRKYGQKQVKGSEFPRSYYKCTSSGCPVKKKVERSQDGQVTEIVYKGEHNH

>Sm31 C

DILDDGYRWRKYGQKIVKGNPYPRSYYKCTNVGCPVRKHVERASNDPKSVITTYEGKHNH

>Sm32

DIMDDGYRWRKYGQKAVKNSPHPRSYYRCTNTKCPVKKRVERSSEDQGLVITTYEGIHNH

>Sm33

AQMNDGCQWRKYGQKMAKGNPCPRAYYRCTVAPGCPVRKQVQRCADDMSILITTYEGSHNH

>Sm34

KIADIPQDERKYGQKPIKGSPHPRYYKCSSLRGCPARKHVERCLDDPTTDAGEHSH

>Pp1

DIPSDEYSWRKYGQKPIKGSPHPRGYYKCSSIRGCPARKHVERSMEDSSMLIVTYEGDHNH

>Pp14

EIMEDGYKWRKYGQKAVKNSPHPRSYYRCTNPKCPVRKKVERSADDSESVITTYEGTHTH

>Pp7

DIPPDEYSWRKYGQKPIKGSPHPRGYYKCSSIRGCPARKHVERSMEDSTMLIVTYEGEHNH

>Pp9

DIPPDDYSWRKYGQKPIKGSPHPRGYYKCSSIRGCPARKHVERSMEDPTMLIVTYEGEHNH

>Pp3

DVMEDGYKWRKYGQKAVKDSPFPRSYYRCTNQTCPVRKRVERKAGDAGLVVTTYEGTHSH

>Pp30

DMVEDGYKWRKYGQKTVLSSPYPRSYYKCTTAGCRVRKQVSRCVEDRGLVIASYEGEHHH

>Pp4 N

RPSEDGYNWRKYGQKHVKGSEYPRSYYKCTHINCLMKKKVERSRDGQVTEIIYKGDHNH

>Pp4 C

DILDDGYRWRKYGQKVVKGNPHPRSYYKCTNVGCPVRKHVERASNDPKAVITTYEGKHNH

>Pp33

QTKGDGCHWRKYGQKMAKGNPCPRAYYRCTLLRGCPVRKQVQRCADDLSILITTYEGTHNH

>Pp22

EIMEDGYKWRKYGQKPVKSSPHPRNYYRCTTANCPVRKRVERSIEDPGLIVTSYEGTHTH

>Pp10

STINDGCQWRKYGQKMAKGNPCPRAYYRCTVMSGCPVRKQVQRCAKDTSILVSTYEGTHNH

>Pp11

DVMDDGYKWRKYGQKPVKSSPHPRNYYRCTTPNCPVRKRVERSTEDPDQVITTYEGRHTH

>Pp12

DVMEDGYKWRKYGQKPVKNSPHPRNYYRCTTAHCPVRKRVERSTEDPGLVITSYEGTHSH

>Pp13

PTINDGCQWRKYGQKMAKGNPCPRAYYRCTVAPGCPVRKQVQRCADDISILVTTYEGTHNH

>Pp5

ATMNDGCQWRKYGQKMAKGNPWPRAYFRCTVSPGCPVRKQVQRCEEDTSILVTTYEGTHNH

>Pp15

DVIDDGYKWRKYGQKPVKNSVHPRNYYKCTTANCPVRKRVERCTDDPSHVLTTYDGTHTH

>Pp16 N

RPSEDGYNWRKYGQKQVKGSEYPRSYYKCTQANCPMKKKVERSHDGQVTEIVYKGDHNH

>Pp16 C

DILDDGYRWRKYGQKVVKGNPHPRSYYKCTNVGCPVRKHVERASTDIKAVITTYEGKHNH

>Pp17

DIMEDGYKWRKYGQKAVKNSPYPRSYYRCTNPDCPVRKRVERKADDHGLVVTTYEGTHNH

>Pp18

DIMDDGFKWRKYGQKAVKNSPHPRNYYRCTTPQCPVRKRVERSSEDAGLVITTYEGTHTH

>Pp19

PTMNDGCQWRKYGQKMAKGNPCPRAYYRCTVAPGCPVRKQVQRCADDVSILITTYEGTHNH

>Pp20

DVLEDGYKWRKYGQKAVKNSTHPRSYYRCTSHTCPVRKRIERKADDPGLVITTYEGTHNH

>Pp21

DVLDDGYKWRKYGQKAVKNSPHPRNYYRCATPNCPVRKRVERCIEDPGLVATAYEGTHSH

>Pp6

DVIDDGYKWRKYGQKPVKSSPHPRNYYRCTTANCPVRKRVERSIEDPGLIVTSYEGTHTH

>Pp23 N

RPSEDGYNWRKYGQKQVKGSEYPRSYYKCTQTNCPMKKKVERSHDGQVTEIVYKGDHNH

>Pp23 C

DILDDGYRWRKYGQKVVKGNPHPRSYYKCTNVGCPVRKHVERASTDIKAVITTYEGKHNH

>Pp24

DIMDDGFKWRKYGQKAVKNSPYPRNYYRCTTPQCPVRKRVERSCEDSGLVITTYEGTHTH

>Pp25

DIPPDDYSWRKYGQKPIKGSPHPRYYKCSSMRGCLARKHVERSLEDSSMLIITYEGEHNH

>Pp26

DIIDDGYRWRKYGQKPVKNSHHPRNYYKCTTPNCLVKKQVERCTENPSNVMTTYYGTHNH

>Pp27

DVMDDGYKWRKYGQKAVKKSPHPRNYYRCTTLNCPVRKRVERCFDDPGVMVTTYEGTHTH

>Pp28

DIMEDGYKWRKYGQKAVKNSPHPRSYYRCTNPKCPVRKKVERSADDSELVITSYEGTHTH

>Pp29

DVLDDGFKWRKYGQKAVKNSPHPRNYYRCTTPLCPVRKRVERSNEDAGLVITTYEGTHSH

>Pp32

VQMNDGCQWRKYGQNTAKGNLCPRAYYRCTVVHCCPVRKQVQRCAHDKSVLITTYEGTHNH

>Pp31

DVLDDGFKWRKYGQKAVKNSPHPRNYYRCTTPLCPVRKRVERSKEDAGLVITTYEGTHSH

>Pp2

DIPPDDYSWRKYGQKPIKGSPHPRGYYKCSSIRGCPARKHVERSMEDPTMLIVTYEGEHNH

>Pp34

IPDDGHRGWKKYGNKAIQNSNHCRGYYKCSLKECRAKKMVQLTDRDPTLFEVTYVGKHSC

>Pp35

IPDDGHRGWKKYGNKSIQNSNHCRGYYKCSVKECRAKKMVQPTDKDPMVFEITYVGKHTC

>Pp36

IPDDGHRGWKKYGNKSIQNSNHCRGYYKCSVKECRAKKMVQPTDTDPTVFEVTYVEKHTC

>Pp37

IPDDGHRCWKKYGNKAIQNSTFCRGYYKCSMKECRAKKMVQPTDTNPSIFEVTYLGKHTC

>Pp38

IPDDDHRGWKKYGNKTIQNSNFCRGYYKCGMVDCRAKKMVQPTVQDPSIFEVTYVGKHTC

>Ot1 alga

HAHEDGYRWRKYGQKNIKGSRHPRSYYRCTERGCPARKKTELASDDESDEDEGDRMRVTYEGVHTH

>Ot2 alga

EKLDDGFRWRKYGNKMLSGQPHPRAYYKCTSGCGKGFLQKHVERIASKGREKHLFLVTYYGRSPGC

>Ot3 alga

VDENDGFRWRKYGCKLIAGNMVPHPTERSYYRCKHFGCPARKRVEVEKVTGATRTVYEFEHTC

>Ol1 alga

SAHADGYRWRKYGQKNIKGSSFPRSYYRCTERGCPARKKTELRRASEDGEMETVVCYEGEHTH

>Ol3 alga

RCDDDAHRWRKYGHKKIGKRDKTSNDLSERSYYRCSFEGCPARKRVGWCPETGHTRVWYEFEHTC

>Mp1 alga

PGVDDGYRWRKYGQKIIKGAPFPRSYYRCTSANCPARKHVEGDPSLLSSLTYEGEHNH

>Mp2 alga

PMDADGYNWRKYGCRVNVAFDARTTYFKCATPGCGAKKRMVTSLHTCRTTGAIMDGETVHVH

>Cr1 N alga

VANSDGWQWRKYGEKLVKGSPNPRSYYKCSHPGCLAKKIVERSDSDGTVLSTEYKGDHCH

>Cr1 C alga

TNMDDGYRWRKYGQKQVKGSPFPRAYYKCTHMGCSVRKHVERSAEDETRFVVTYEGTHSH

>Vc1 N alga

VANSDGWQWRKYGEKLVKGSPNPRSYYKCSHPGCLAKKIVERSEADGNVLSTEYKGDHCH

>Vc1 C alga

VNMDDGFRWRKYGQKQVKGSPFPRAYYKCTHAGCTVRKHVERSAEDETRFVVTYEGTHNH

>Vc2 alga

RDLPNEYRWRKYGEKWIDGMCRSYFRCSMLYCPARLRAEWRPSGGSGLGGSGGGGGESGGGDANAGGFILWVSYDAPHNH

>Vc3 alga

RDLPNEYRWRKYGEKWIDGMCRSYFRCSMLYCPARLRAEWRPSGGGGGGLDGSGGGRGRGANASGGGAGESGGGDGNAGGFILWVSYDAPHNH

>Cc1 N alga

NDDGYHWRKYGEKQVKGSPYPRSYYKCSQQNCQVKKIVERNPENGEVSKSASKGVHNH

>Cc1 C alga

DSMDDGYRWRKYGQKIVKGNPHPRSYYKCTVAGCTVRKHVGRSATEAGVLVTSYEGQHNH

>Cn1 N alga

MANDDGYNWRKYGEKQVKGSPFPRSYYKCSHPGCPAKKMIEREPKTGRISQAELKNEHNH

>Cn1 C alga

DGMDDGYRWRKYGQKIVKGNPHPRSYYKCTHPGCNVRKQVERSGRNARMLVTTYEGTHTH

>Or1 alga

VANADGYRWRKYGQKNIKGSRHPRSYYRCTERGCPARKKTELVDGARNETASRGVAEKGDDGTTGPKTPVLRVTYEGEHTH

>Or3 alga

IEGEEDISWRKYGRKYIMKETLTPIERSYYRCSVPGCPARKHVCVRSGSDKLEVSYMHDHTC

>Bp1 alga

HKTLDGFKWRKYGRKNIKLASSQVTRSYYRCTRPNCPARKQVDENGLVKYENAHNH

>Bp2 alga

ESDSDGYSWRKYGEKELKSGSSTKSYYRCARLDCPAKKTIERGMTVYTYAEHTH

>Ap1 C alga

DNLDDGYRWRKYGQKMVKGNPHPRSYYKCTHPGCCVRKQLDCMLG

>Ap1 N alga

TANEDGYNWRKYGEKHVKGSSCPRSYYKCSFPGCQVKKIIERNLKTGFVSVCLSKPPDRHPGRRAVGRGGRGLRRRLVGLPLAPGLHTH

>Bp3 alga

HDIKDGFKWRKYGQKNIKSASFPRSYYRCTKPDCPARKQVENGLAKYENQHNH

>Cs1 C alga

DSMDDGYRWRKYGQKIVKGNPHPRSYYKCTVAGCTVRKHVGRSATEAGVLVTSYEGQHNH

>Cs1 N alga

NDDGYHWRKYGEKQVKGSPYPRSYYKCSQQNCQVKKIVERNPENGEVSKSASKGVHNH

>Ac1 N Amoebozoa

SPESDGYRWRKYGRKTVKGSPYPRSYFKCTFPHCPVKKQVEAVIRDGHIVSTSSIYKAKHNH

>Ac1 C Amoebozoa

DYLDDGYRWRKYGQKYVKGSGYPRSYYKCTDKQCPVKKQVDALLVGLVVTYEGAHTH

>Ac2 Amoebozoa

HLPHDGYKWRSDGRDKKWPQTTKIYYKCISVEGCSAKLHFFPHTKEIQYVGEHNH

>Mv1 Fungus

DQHIDYYVWKNNGNTVQKKTGCKSIYFKCSNSAAGCTVNKTVTEKEGGGYLTKYRGEHLEDC

>Mv2 Fungus

QPHIDPYLWKNNGNTIQKKTGCKSIYYKCSNSTAGCTVNKTVTEKEGGGYVTKYRGEHLEEC

>Mc1 fungus

YPYVDGFFWKNNGNTIQKKTGNKSVYYKCSNSSKGCPVNKTVTWKENGEYLIKYRGEHLAEC

>Ai1 fungus

YPYVDGFFWKNNGNTVQKKTGNKSIYYKCSNSGKGCPVNKTVSWKENGEYLIKYRGEHLPDC

>Mc2 fungus

FPYIDNYFWKNNGNTVQKKTGCKSIYYKCSNSNKGCSVNKTVTARPNGEYLIKYRGCHLQEC

>Lc1 fungus

FPYNDDYLWKNNGNTVHKKSGYRSIYYKCSNSAKGCPVNKTVTFKEHGTYLIKYRGEHLEEC

>Rd1 fungus

NPYNDGFFWKNNGNTIQKKTGNKSIYYKCSNSTKGCPVNKTVTAKENGEYLIKYRGEHLPDC

>Mc3 fungus

YPYVDGYFWKNNGNTVQKKTGNKSVYYKCSNSNKGCPVNKTVTWKDQGEYLIKYRGEHLLDC

>Lc2 fungus

YPYVDGFFWKNNGNTVQKKTGNKSIYYKCSNSGKGCPVNKTVSWKENGEFLIKYRGEHLPDC

>Lc7 fungus

YPYADGYFWKNNGNTIQKKTGNKSVYYKCSNSAKGCPVNKTVTWKGNGEYLIKYRGEHLPDC

>Ai2 fungus

YPYMDGFFWKNNGNTVQKKTGNKSIYYKCSNSSKGCPVNKTVTWKENGEYLIKYRGEHLPEC

>Lc3 fungus

YPYMDGFFWKNNGNTVQKKTGNKSIYYKCSNSSKGCPVNKTVTWKENGEYLIKYRGEHLPEC

>Rd2 fungus

YPYMDGYFWKNNGNTVQKKTGNKSVYYKCSNSNKGCPVNKTVTWKGNGEYLIKYRGEHLIEC

>Ai3 fungus

YPYVDGYFWKNNGNTIQKKTGNKSVYYKCSNSVKGCPVNKTVTWKGNGEYLIKYRGEHLPDC

>Rd3 fungus

YPYMDGYFWKNNGNTVQKKTGNKSVYYKCSNSNKGCPVNKTVTWKGNGEYLIKYRGEHLLEC

>Ai8 fungus

YPYLDGYSWKNNGNTVQKKTGNKSVYYKCSNSIRGCPVNKTVTWKGNDEYLIKYRGEHLPDC

>Ri1 fungus

HPYVDDYLWKNNGNTTQRKTGNKSVYFKCSNSNKGCPVNKTVTEKECGWIIKYRGNHLEEC

>Lc4 fungus

FPYQDNYFWKNNGNTTQKKTGCKSIYYKCSNSNKGCPVNKTVTARPNGEYLIKYRGLHLSEC

>Ai4 fungus

FPYQDNYFWKNNGNTTQKKTGCKSIYYKCSNSNKGCPVNKTVTARPNGEYLIKYRGLHLSEC

>Lc5 fungus

FPYHDDYLWKNNGNTVHKASGTKSIYYKCANHPKGCPVNKTVTFKEHGQYLIKYRGEHLNTC

>Lc6 fungus

YPYLDGYSWKNNGNTVQKKTGNKSVYYKCSNSIRGCPVNKTVTWKGNDEYLIKYRGEHLPDC

>Ai5 fungus

FPYNDDYLWKNNGNTVHKKSGYRSIYYKCSNSAKGCPVNKTVTFKEHGTYLIKYRGEHLEEC

>Ai6 fungus

FPYHDDYLWKNNGNTVHKASGTKSIYYKCANHPKGCPVNKTVTFKEHGQYLIKYRGEHLNTC

>Mc4 fungus

FPYNDDYLWKNNGNTVHKKTGFKSIYYKCSNSAKGCPVNKTVTFKDNGEHLIKYRGQHLPEC

>Mc5 fungus

FPYDDRHFWKNNGNTVQKKTGCKSVYYKCANSCKGCPVNKTVVEQPDGSYIIKYRGDHISEC

>Ai7 fungus

FPYNDDYLWKNNGNTIHKGSGNKSIYYKCANNPKGCPVNKTVTFKDNGEYLIKYRGKHLSDC

>Gl1 N Diplomonad

ELPADGYCWRKYGSKRLPNNSHPKSYFRCSVPGCQAKRYVTETDNRVLKTEYIGEHNH

>Gl1 C Diplomonad

ESSIDFFRWKKYGHKPQTDTRLDSKSYYRCAFFNCPARRTITFFYSLSSDGTETIESMIVQYENQHTH

>Gl2 N Diplomonad

ELPADGYCWRKYGSKRLPNNSHPKSYFRCSVPGCQAKRYVTETDNRVLKTEYIGEHNH

>Gl2 C Diplomonad

ESSIDFFRWKKYGHKPQTDTRLDSKSYYRCAFFNCPARRTITFFYSLSSDGTETVESVIVQYENQHTH

>Gi1 N Diplomonad

ELPADGYCWRKYGSKRLPNNSHPKSYFRCSVPGCQAKRYVTETDNRVLKTEYIGEHNH

>Gi1 C Diplomonad

ESSIDFFRWKKYGHKPQTDTRLDSKSYYRCAFFNCPARRTITFFYSLSSDGTETVESVIVQYENQHTH

>Gi2 N Diplomonad

ELPADGYCWRKYGSKRLPNNAYPKSYFRCSVPGCQAKRYVTETDNRVLKTEYIGEHNH

>Gi2 C Diplomonad

ESCIDFFRWKKYGHKPQTDTRLDSKSYYRCAFFNCPARRTITFFYSINPDGTETIESMIVQYDNQHTH

>Gi3 N Diplomonad

ELPADGYCWRKYGSKRLPNNAYPKSYFRCSVPGCQAKRYVTETDNRVLKTEYIGEHNH

>Gi3 C Diplomonad

ESCIDFFRWKKYGHKPQTDTRLDSKSYYRCAFFNCPARRTITFFYSINPDGTETIESMIVQYDNQHTH

>Ss1 Diplomonad

DLQDDGFRWAKYGVNGPRHYFRCDVPACVAKRIAQRTGGHVLSSYRGVHSH

>Dd1 N social amoebae

IVSDGYQQWRKYGQKNVKGSLHPRHYYKCTFQGCNVRKQVERIGDTNQNSTVYKGEHCH

>Dd1 C social amoebae

DHLDDGFFWRKYGQKSVKGSPFPKSYFKCAELTCPVKKQVIQQDSKYINTYRGKHNH

>Ppa1 N social amoebae

ISSDGYQWRKYGQKNVKGTQFPRSYYKCTYPGCTVKKQMERRSSSDDTLNHVVYKGEHNH

>Ppa1 C social amoebae

DHLDDGFNWRKYGQKAVKGSPYPKSYFKCAEHGCNVKKQVIQQGDKKFVNTYNGRHTH

>Df1 N social amoebae

IIAADGYQWRKYGQKNVKGTQFPRSYYKCTVPGCTVKKQVEKLSETDETKNRVVYKGTHNH

>Df1 C social amoebae

DHLDDGFNWRKYGQKAVKGTHFPKSYFKCAEPGCSVKKQVLQQSENSFLSTYNGNHNH

>Fa1 C social amoebae

DAMDDGYIWRKYGQKAVRGAPFPRAYYRCTTKGCPVKKQIRRGYGQVISSYEGVHNH

>Fa1 N social amoebae

GVHEDGYRWRKYGQKQRRSSPFPKAYFRCTGPGCPAKKQIEVMVLGDQRLTSSVYRGQHNH

>Dp1 social amoebae

DHLDDGFSWRKYGQKAVKGSIFPKSYFKCAEPSCPVKKQVIQQDLKFINTYSGKHNH

>Dp2 social amoebae

NIISDGYQWRKYGQKNVKGSSHPRHYYKCTYPGCNVRKQVERVSNGSNTNNIVYKGEHCH

>Df2 social amoebae

LPTLDEYHWLLNTIKFVNNSKFLCQNYTCFEKECKATKNVQKLSPNNNNFRVTYINTHNH

>Df3 social amoebae

SKQQLPYSWNRMGSTKADGINFTKYQYNCSVTDCECIKLVQHYPINHDNNRYIELIIYQGVHNH

>Gm1

PLISDGCQWRKYGQKIAKGNPCPRAYYRCTMAVGCPVRKQVQRCMDDKTVLITTYEGNHNH

>Gm1/7 N

KTSDDGYNWRKYGQKLVKGSEFPRSYYKCTHPNCEVKKLFERSHDGQITEIVYKGTHDH

>Gm1/7 C

DILDDGYRWRKYGQKVVRGNPNPRSYYKCTNAGCPVRKHVERASHDPKAVITTYEGKHNH

>Gm2

NLSADLWAWRKYGQKPIKGSPYPRCSSSKGCMARKQVERSNTEPDMFVVTYTGDHSH

>Gm3 N

KTSDDGYNWRKYGQKLVKGSEFPRSYYKCTHPNCEVKKLFERSHDGQITEIVYKGTHDH

>Gm3 C

DILDDGYRWRKYGQKVVRGNPNPRSYYKCTNAGCPVRKHVERASHDPKAVITTYEGKHNH

>Gm4 N

KPADDGYNWRKYGQKQVKGSEYPRSYYKCTHLNCVVKKKVERAPDGHITEIIYKGQHNH

>Gm4 C

DLLDDGYRWRKYGQKVVKGNPHPRSYYKCTSAGCNVRKHVERASMDPKAVITTYEGKHNH

>Gm5 N

RRSEDGYNWRKYGEKQVKGSENPRSYYKCTHPSCPTKKKVERSLEGHITEIVYKGSHNH

>Gm5 C

DILDDGYRWRKYGQKVVKGNPNPRSYYKCVAPGCPVRKHVERASHDMKAVITTYEGKHIH

>Gm6

EIMDGGYKWRKYGKKSVKSSPNLRNYYKCSSGGCSVKKRVERDRDDYSYVITTYEGVHNH

>Gm7

NLSSDLWAWRKYGQKPIKGSPYPRNYYRCSSCKGCVARKQVERSTTEPNTFIVTYTGDHKH

>Gm8

GLPSDFWSWRKYGQKPIKGSPYPRGYYKCSTSKGCSAKKQVERCRTDASMLIITYTSTHNH

>Gm9 N

KPADDGYNWRKYGQKQVKGSEFPRSYYKCTHPNCSVKKKVERSLEGHVTAIIYKGEHNH

>Gm9 C

DLLDDGYRWRKYGQKVVKGNPYPRSYYKCTTQGCNVRKHVERASTDPKAVITTYEGKHNH

>Gm10

NLSSDIWAWRKYGQKPIKGSPYPRGYYRCSSSKGCLARKQVERNRSDPTMFIVTYTAEHNH

>Gm11

DIPPDDYSWRKYGQKPIKGSPHPRGYYKCSSVRGCPARKHVERALDDPAMLVVTYEGEHNH

>Gm12

GPLEDGYSWRKYGQKDILSAKYPRSYYRCTFRKTKGCFATKQVQRSEEDHTIFDITYRGSHTC

>Gm13

DIPVDEYSWRKYGQKPIKGSPYPRGYYKCSSVRGCPARKHVERAQDDPNMLIVTYEGEHRH

>Gm14

DIPPDDYSWRKYGQKPIKGSPHPRGYYKCSSVRGCPARKHVERALDDPSMLVVTYEGEHNH

>Gm15

DIPPDDYSWRKYGQKPIKGSPHPRGYYKCSSTRGCPARKHVERCLEEPSMLIVTYEGDHNH

>Gm16

YPPSDSWAWRKYGQKPIKGSPYPRGYYRCSSSKGCPARKQVERSRVDPTKLIVTYAYEHNH

>Gm17

LYVRDGYQWRKYGQKVTRDNPSPRAYFKCSYAPSCPVKKKVQRSVEDPSVLVTTYEGEHNH

>Gm18 N

RVSDDGYNWRKYGQKHVKGSEFPRSYYKCTHPNCEVKKLFERSHDGQITEIIYKGTHDH

>Gm18 C

DILDDGYRWRKYGQKVVRGNPNPRSYYKCTNTGCPVRKHVERASHDPKAVITTYEGKHNH

>Gm19 N

RRSDDGYNWRKYGQKQVKGSENPRSYYKCTYPNCPTKKKVEKSLDGQITEIVYKGTHNH

>Gm19 C

DILDDGYRWRKYGQKVVKGNPNPRSYYKCTFPGCPVRKHVERASQDLRAVITTYEGKHNH

>Gm20

GPHEDGYNWRKYGQKDILGAKYPRSYYRCTFRSTQGCWATKQVQRSDEDPTMFDITYRGNHTC

>Gm21

EVMDDGYKWRKYGKKTVKNNPNPRNYYKCSGEGCNVKKRVERDRDDSNYVLTTYDGVHNH

>Gm22

DIPPDDYSWRKYGQKPIKGSPHPRGYYKCSSMRGCPARKHVERCLEEPTMLIVTYEGEHNH

>Gm23

PMISDGCQWRKYGQKMAKGNPCPRAYYRCTMAVGCPVRKQVQRCADDRTILVTTYEGTHNH

>Gm24 N

TSASDGYNWRKYGQKQVKSPTGSRSYYRCTHSDCCAKKIECCDDSGHVIEIVYKSEHSH

>Gm24 C

GISADGYRWRKYGQKMVKGNPHPRNYYRCTSAGCPVRKHIETAVDNSDAVIITYKGVHDH

>Gm25

DILDDGYRWRKYGEKSVKNNKFPRNYYRCSYRGCNVKKQIQRHSKDEEIVVTTYEGIHIH

>Gm26

DHLDDGYKWRKYGQKAVKNSPYPRSYYRCTSAGCGVKKRVERSSEDPSMVVTTYEGQHTH

>Gm27

LIVKDGYQWRKYGQKVTRDNPSPRAYFKCSFAPSCPVKKKVQRSVDDQSVLVATYEGEHNH

>Gm28

DHLEDGYRWRKYGQKAVKNSPFPRSYYRCTNSKCTVKKRVERSSEDPTIVITTYEGQHCH

>Gm29 N

DHLEDGYRWRKYGQKAVKNSPFPRSYYRCTNSKCTVKKRVERSSEDPTIVITTYEGQHCH

>Gm29 C

EVLGDGFRWRKYGQKVVKGNPYPRSYFRCTNIMCNVRKHVERAIDDPRSFVTTYEGKHNH

>Gm30

MYVMDGYQWRKYGQKVTRDNPSPRAYFRCSFAPSCPVKKKVQRSLEDPTILVTTYEGEHNH

>Gm31

DIPPDEYSWRKYGQKPIKGSPYPRGYYKCSTVRGCPARKHVERAPDDPAMLIVTYEGEHRH

>Gm32

DHLEDGYRWRKYGQKAVKNSPFPRSYYRCTNSKCTVKKRVERSSEDPTIVITTYEGQHCH

>Gm33

DIPADEYSWRKYGQKPIKGSPYPRGYYKCSSVRGCPARKHVERAQDDPNMLIVTYEGEHRH

>Gm34

PMISDGCQWRKYGQKMAKGNPCPRAYYRCTMAVGCPVRKQVQRCAEDRTILTTTYEGTHNH

>Gm35

TPPSDSWAWRKYGQKPIKGSPYPRGYYRCSSSKGCPARKQVERSCVDPTMLVVTYSSDHNH

>Gm36

PMITDGCQWRKYGQKMAKGNPCPRAYYRCTMAAGCPVRKQVQRCAEDRTILITTYEGNHNH

>Gm37

DIPPDEYSWRKYGQKPIKGSPYPRGYYKCSSVRGCPARKHVERAPDDPTMLIVTYEGEHRH

>Gm38

DVLDDGYKWRKYGQKVVKNSLHPRSYYRCTHNNCRVKKRVERLSEDCRMVITTYEGRHNH

>Gm39 N

RRSDDGYNWRKYGQKQVKGSENPRSYYKCTYPNCPTKKKVERSLDGQITEIVYKGTHNH

>Gm39 C

DILDDGYRWRKYGQKVVKGNPNPRSYYKCTHPGCPVRKHVERASHDLRAVITTYEGKHNH

>Gm40

DVLDDGYKWRKYGQKVVKNTQHPRSYYRCTQDNCRVKKRVERLAEDPRMVITTYEGRHVH

>Gm41

GLPSDFWSWRKYGQKPIKGSPYPRGYYKCSTSKGCSAKKQVERCRTDASMLIITYTSTHNH

>Gm42

DIPSDEYSWRKYGQKPIKGSPYPRGYYKCSTVRGCPARKHVERAQDNPKMLIVTYEGEHRH

>Gm43

GSLDDGYSWRKYGQKDILGAKFPRGYYRCTQRNVQGCLATKQVQRSDEDPTTIEVTYRGRHTC

>Gm44 N

ASASDGYNWRKYGQKQVKNPMGSRSYYKCTHSNCCAKKIKFCDHSGHVIEIVYKSQHNH

>Gm44 C

GISGDGYRWRKYGQKLVKGNPHFRNYYRCTSSGCPVRKHIETAVDNSKALIITYKGVHDH

>Gm45

DVLDDGYKWRKYGQKVVKNTQHPRSYYRCTQDNCRVKKRVERLAEDPRMVITTYEGRHVH

>Gm46

GPHEDGYNWRKYGQKDILGAKYPRSYYRCTFRSTQGCWATKQVQRSDEDPTMFDITYRGNHTC

>Gm47

DIPPDEYSWRKYGQKPIKGSPYPRGYYKCSTVRGCPARKHVERAQDDPKMLIVTYEGEHRH

>Gm48

TPPSDSWAWRKYGQKPIKGSPYPRGYYRCSSSKGCPARKQVERSCVDPTMLVVTYSSDHNH

>Gm49 N

RRSDDGYNWRKYGQKQVKGSENPRSYYKCTYPNCPTKKKVERSLDGQITEIVYKGTHNH

>Gm49 C

DILDDGYRWRKYGQKVVKGNPNPRSYYKCTHPGCPVRKHVERASHDLRAVITTYEGKHNH

>Gm50

DIPPDEYSWRKYGQKPIKGSPYPRGYYKCSTVRGCPARKHVERASDDPTMLIVTYEGEHRH

>Gm51

DHLEDGYRWRKYGQKAVKNSPFPRSYYRCTSVSCNVKKRVERSFSDPSIVVTTYEGQHTH

>Gm52

YPPSDSWAWRKYGQKPIKGSPYPRGYYRCSSSKGCPARKQVERSRVDPTXLIVTYAYEHNH

>Gm53

DILDDGYRWRKYGQKAVKNNKFPRSYYRCTHQGCNVKKQVQRLTKDEGVVVTTYEGVHTH

>Gm54

DHLEDGYRWRKYGQKAVKNSPFPRSYYRCTSVSCNVKKRVERSFTDPSVVVTTYEGQHTH

>Gm55

GSLDDGYSWRKYGQKDILGAKFPRGYYRCTYRNVQGCLATKQVQKSDEDPMICEITYKGRHTC

>Gm56

LIVKDGYQWRKYGQKVTRDNPYPRAYFKCSFAPSCPVKKKVQRSVDDHSVLLATYEGEHNH

>Gm57

APIDDGHQWRKYGQKEILSAKFPRNYYRCTHKFDQGCQATKQVQRVQEEPILYKTTYYGLHTC

>Gm58

QTTDDNHAWRKYGQKEILNSQFPRSYFRCTRKFEQGCRATKQVQRIQENPDMYTITYIGFHTC

>Gm59 N

APSEDGYNWRKYGQKQVKGSEYPRSYYKCTHPNCQVKKKVERSHEGHITEIIYKGTHDH

>Gm59 C

DILDDGYRWRKYGQKVVKGNPNPRSYYKCTNAGCTVRKHVERASHDLKSVITTYEGKHNH

>Gm60

GSLDDGYSWRKYGQKDILGAKFPRGYYRCTYRNVQGCLATKQVQKSDEDPMICEITYKGRHTC

>Gm61

EVMDDGYKWRKYGKKTVKSSPNPRNYYKCSGEGCDVKKRVERDRDDSNYVLTTYDGVHNH

>Gm62 N

RRSDDGYNWRKYGQKQVKGSENPRSYYKCTYPNCPTKKKVERSLDGQITEIVYKGTHNH

>Gm62 C

DILDDGYRWRKYGQKVVKGNPNPRSYYKCTFPGCPVRKHVERASQDLRAVITTYEGKHNH

>Gm63

LIVKDGYQWRKYGQKVTKDNASPRAYFRCYMAPICPAKKKVQRCLHDKSILVAIYDGEHSH

>Gm64

DILDDGYRWRKYGQKAVKNNTYPRSYYRCTHHTCNVKKQVQRLSKDTSIVVTTYEGIHNH

>Gm65 N

KVSKDGYNWRKYGQKHVKGNEFIRSYYKCTHPNCLAKKQLQQSNNGHITDSICIGQHNH

>Gm65 C

DLVNDGYRWRKYGQKLVKGNTNPRSYYRCSNPGCPVKKHVERASHDSKVVITTYEGQHDH

>Gm66 N

KPNNDGYNWRKYGQKHVKGSDFSRSYYKCTRPNCPVKKKLERSLEGHVTAIIYKGEHNH

>Gm66 C

NLLDDGYRWRKYGQKVVKGNPYPRSYYKCTTQGCKVRKHVERASMDPKAVITTYEGKHNH

>Gm67

LIVKDGYQWRKYGQKVTRDNPSPRAYFKCSFAPSCPVKKKVQRSVDDQSVLVATYEGEHNH

>Gm68 N

KVSKDGYNWRKYGQKHVKGNEFIRSYYKCTHPNCQAKKQLQQSNNGHITDSICIGQHNH

>Gm68 C

DLVNDGYRWRKYGQKLVKGNTNPRSYYRCSNPGCPVKKHVERASYDSKTVITTYEGQHDH

>Gm69

DHLEDGYRWRKYGQKAVKNSPFPRSYYRCTSVSCNVKKRVERSFTDPSVVVTTYEGQHTH

>Gm70

DVLDDGYKWRKYGQKVVKNSLHPRSYYRCTHNNCRVKKRVERLSEDCRMVITTYEGRHNH

>Gm71

LIVKDGYQWRKYGQKVTRDNPCPRAYFKCSFAPSCPVKKKVQRSVDDQSVLVATYEGEHNH

>Gm72

DIPPDDYSWRKYGQKPIKGSPHPRGYYKCSSMRGCPARKHVERCLEEPTMLIVTYEGEHNH

>Gm73

PMISDGCQWRKYGQKMAKGNPCPRAYYRCTMAVGCPVRKQVQRCADDKAVLITTYEGNHNH

>Gm74

APIDDGHHWRKYGQKEILNAKFPRNYYRCTHKFDQGCQATKQVQRVQEEPILFKTTYYGHHTC

>Gm75

PVMHDGCQWRKYGQKIAKGNPCPRAYYRCTLAPACPVRKQVQRCADDMSILITTYEGTHNH

>Gm76

QTTDDNHAWRKYGQKEILNSQFPRSYFRCTRKFEQGCRATKQVQRIQENPDRYNITYIGFHTC

>Gm77

VVPSDLWAWRKYGQKPIKDSPYPRGYYRCSSSKGCPARKQVERSRTDPNMLVITYTSEHNH

>Gm78 N

RVSDDGYNWRKYGQKHVKGSEFPRSYYKCTHPNCEVKKLFERSHDGQITEIIYKGTHDH

>Gm78 C

DILDDGYRWRKYGQKVVRGNPNPRSYYKCTNTGCPVRKHVERASHDPKAVITTYEGKHNH

>Gm79 N

KPADDGYNWRKYGQKQVKGSEFPRSYYKCTNPNCPVKKKVERSLEGHVTAIIYKGEHNH

>Gm79 C

DLLDDGYRWRKYGQKVVKGNPYPRSYYKCTTQGCNVRKHVERASTDPKAVITTYEGKHNH

>Gm80 N

KPADDGYNWRKYGQKQVKGSEYPRSYYKCTHLNCVVKKKVERAPDGHITEIIYKGQHNH

>Gm80 C

DLLDDGYRWRKYGQKVVKGNPHPRSYYKCTSAGCNVRKHVERASTDPKAVITTYEGKHNH

>Gm81 N

ASASDGYNWRKYGQKQVKSPMGSRSYYRCTHSYCCAKKIKFCDHSGHVIEIVYKSQHSH

>Gm81 C

GISGDGYRWRKYGQKLVKGNPHFRNYYRCTTAGCPVRKHIETAVDNSKALIITYKGMHDH

>Gm82

LIVKDGYQWRKYGQKVTKDNASPRAYFRCSMAPMCPVKKKVQRCLHDKSIVVATYDGEHNH

>Gm83

DIPPDDYSWRKYGQKPIKGSPHPRGYYKCSSVRGCPARKHVERALDDPSMLVVTYEGEHNH

>Gm84

QQISDGCQWRKYGQKIAKGNPCPRAYYRCTMAVGCPVRKQVQRCMEDKTVLITTYEGNHNH

>Gm85

DHLEDGYRWRKYGQKAVKNSPFPRSYYRCTSVSCNVKKRVERSFSDPSIVVTTYEGQHTH

>Gm86

SLMGDGCQWRKYGQKISKGNPCPRAYYRCNMGTACPVRKQVQRCAEDESVVITTYEGNHNH

>Gm87 N

KRAEDGFNWRKYGQKQVKGSENPRSYYKCTHPNCSVKKKVEKTLEGQITEIVYKGQHNH

>Gm87 C

DILDDGFRWRKYGQKVVKGNPNARSYYKCTAPGCSVRKHVERAAHDIKAVITTYEGKHNH

>Gm88

DILDDGYRWRKYGQKAVKNSTYPRSYYRCTHHTCNVKKQVQRLSKDTSIVVTTYEGIHNH

>Gm89

PMIADGCQWRKYGQKMAKGNPCPRAYYRCSMASACPVRKQVQRCAEDRTVLITTYEGNHNH

>Gm90

PMISDGCQWRKYGQKMAKGNPCPRAYYRCTMAVGCPVRKQVQRSADDKTVLITSYEGNHNH

>Gm91

DILDDGYRWRKYGQKAVKNNKFPRSYYRCTHQGCNVKKQVQRLTKDEGVVVTTYEGVHTH

>Gm92

DIPPDDYSWRKYGQKPIKGSPHPRGYYKCSSVRGCPARKHVERALDDPAMLVVTYEGEHNH

>Gm93 N

APSEDGYNWRKYGQKQVKGSEYPRSYYKCTHPNCQVKKKVERSHEGHITEIIYKGTHNH

>Gm93 C

DILDDGYRWRKYGQKVVKGNPNPRSYYKCTNAGCTVRKHVERASHDLKSVITTYEGKHNH

>Gm94

PMISDGCQWRKYGQKMAKGNPCPRAYYRCTMAVGCPVRKQAQRCTDDRTILVTTYEGTHNH

>Gm95

DHLEDGYRWRKYGQKAVKNSPYPRSYYRCTSQKCGVKKRVERSFQDPTIVITTYEGQHNH

>Gm96 N

TSASDGYNWRKYGQKQVKSPTGSRSYYRCTHSDCCAKKIECCDDSGHVIEIVYKSEHSH

>Gm96 C

GISGDGYRWRKYGQKMVKGNPHPRNYYRCTSAGCPVRKHIETAVDNSDAVIITYKGVHDH

>Gm97

DHLEDGYRWRKYGQKAVKNSPYPRSYYRCTSQKCGVKKRVERSFQDPTIVITTYEGQHNH

>Gm98

SMISDGCQWRKYGQKMAKGNPCPRSYYRCSMGTACPVRKQVQRNAEDLSVLITTYEGQHNH

>Gm99

LTMNDGCQWRKYGQKMAKGNPCPRAYYRCTVSPSCPVRKQVQRCAEDMSILITTYEGTHNH

>Gm100 N

RPSYDGYNWRKYGQKQVKGSEYPRSYYKCTHPNCPVKKKVERSFDGNIAEIVYKGEHNH

>Gm100 C

EILGDGFRWRKYGQKVVKGNPYPRSYYRCTNIKCNVRKHVERAIDDPRSFVTTYEGKHNH

>Gm101

DHLDDGYRWRKYGQKAVKNSPHPRSYYRCTTATCGVKKRVERSSEDPTVVVTTYEGQHTH

>Gm102 N

KPNDDGYNWRKYGQKHVKGRDFSRSYYKCTHPNCPVKKKLERSLEGHVTAIIYKGEHNH

>Gm102 C

DLLDDGYRWRKYGQKVVKGNPYPRSYYKCATQGCNVRKHVERASMDPKAVLTTYEGKHNH

>Gm103 N

RTSEDGYNWRKYGQKQVKGSEYPRSYYKCTQPNCQVKKKVERSHDGQITEIIYKGAHNH

>Gm103 C

DILDDGYRWRKYGQKVVKGNPNPRSYYKCTSTGCMVRKHVERASHNLKYVLTTYEGKHNH

>Gm104

PTMNDGCQWRKYGQKIAKGNPCPRAYYRCTVAPTCPVRKQVQRCAEDLSILITTYEGTHNH

>Gm105

IKMNDGCHWRKYGQKMAKGNPCPRAYYRCTASPSCLVQRCAEEMSILITTYEGTHNH

>Gm106 N

KRTEDGFNWIKYGQKQVKGSENPRSYYKCTHPNCSVKKKVEKSLDGHITEIVYKGQHSH

>Gm106 C

DILDDGFRWRKYGQKVVKGNSNARSYYKCTAPGCSVRKHVERAAHDIKAVITTYEGKHNH

>Gm107 N

RTSEDGYNWRKYGQKQVKGSEYPRSYYKCTQPKCQVKKKVERSHDGQITEIIYKGAHNH

>Gm107 C

DILDDGYRWRKYGQKVVKGNPNPRSYYKCTSAGCMVRKHVERASQNLKYVLTTYEGKHNH

>Gm108

PTMNDGCQWRKYGQKIAKRNPCPRAYYRCTVAPTCPVRRQVQRCAEDLSILITTYEGTHNH

>Gm109 C

DILDDGYRWRKYGQKVVKGNPNPRSYYKCVAPGCPVRKHVERAAHDMKAVITTYEGKHIH

>Gm109 N

KRSEDGYNWRKYGEKQVKGSENPRSYYKCTHPSCPTKKKVERSLEGHITEIVYKGSHNH

>Gm110

QEISDGCQWRKYGQKMAKGNPCPRAYYRCTMAVGCPVRKQVQRCAEDRTILTTTYEGTHNH

>Gm111

DHLDDGYRWRKYGQKAVKNSPHPRSYYRCTTATCGVKKRVERSSEDPTVVVTTYEGQHTH

>Gm112

SLMGDGCQWRKYGQKISKGNPCPRAYYRCNMGTACPVRKQVQRCSEDESVVITTYEGNHNH

>Gm113

PTMNDGCQWRKYGQKISKGNPCPRAYYRCTIAPSCPVRKQVQRCADDKSILITTYEGTHNH

>Gm114

LMVKDGYQWKKYGQKKVTKDNPSPRAYFECSLAPSCSNLKKVQRSIQDKSILVATYEGKHNH

>Gm115

EIMDDGYKWRKYGKKSVKSNPNLRNYYKCSSGGCSVKKRVERDRDDSSYVITTYEGVHNH

>Gm116

GMGDDGYKWRKYGQKSIKNSPNPRSYYRCTNPRCSAKKQVERSNEDPDTLIITYEGLHLH

>Gm117

EVLDDGYRWRKYGKKMVKNSPNPRNYYRCSVDGCNVKKRVERDKDDPRYVITTYEGNHTH

>Gm118

EVLDDGYRWRKYGKKMVKKCPNPRNNYRCSVDGCTVKKRVERDKDDPRYVITTYEGNHTH

>Gm119

EILDDGSKWRKYGKKMVKNSPNPRNYYRCSVDGWQVKKRVERDKDDPRFVITTYEGIHTH

>Gm120

EGLDDAWAWRKYGQKPIKGSPYPRSYYRCSSSKGCLARKQVERSHLDPAVFLVTYTAEHSH

>Gm121

EGLDDAWAWRKYGQKPIKGSPYPRSYYRCSSSKGCLARKQVERSHLDPAVFLVTYTAEHSH

>Gm122

DELDDGYKWRKYGQKVVKGTHHPRSYYRCIQDNCRVKKRVERFAEDPRMVITTYEGRHVH

>Gm123

ARMNDGCQWRKYGQKISKGNPCPRAYYRCTVAPSCPVRKQVQRCAQDMSILFTTYEGNHNH

>Gm124

QTMNDGCHWRKYGQKMAKGNPCPRAYYRCTASPSCPVRKQVQRCAEDMSILITTYEGTHNH

>Gm125

QTMNDGCQWRKYGQKMAKGNPCPRAYYRCTASPSCPVRKQVQRCAEDMSILITTYEGTHNH

>Gm126

ARMNDGCQWRKYGQKISKGNPCPRAYYRCTVAPSCPVRKQVQRCAQDMSILMTTYEGNHNH

>Gm127

ATMNDGCQWRKYGQKIAKGNPCPRAYYRCTVAPGCPVRKQVQRCIDDMSILITTYEGTHNH

>Gm128

DHLEDGYRWRKYGQKAVKNSPYPRSYYRCTTQKCTVKKRVERSFQDPTTVITTYEGQHNH

>Gm129

ATMNDGCQWRKYGQKIAKGNPCPRAYYRCTVAPGCPVRKQVQRCIDDMSILITTYEGTHNH

>Gm130

DHLEDGYRWRKYGQKAVKNSPYPRSYYRCTTQKCTVKKRVERSFQDPTTVITTYEGQHNH

>Gm131

PTMNDGCQWRKYGQKISKGNPCPRAYYRCTIAPSCPVRKQVQRCVDDMSILFTTYEGTHNH

>Gm132

DGVSDPWAWRKYGQKPIKGSAYPRSYYRCSSSKGCLARKHVERSQLDPGVLIVTYTAEHSDPHPTC

>Gm133

FQMGDGCQWRKYGQKTAKGNPCPRAYYRCSMGTACPVRKHVQRCFKDETILITTYEGNHNH

>Gm134

PMITDGCQWRKYGQKMAKGNPCPRAYYRCTMAAGCPVRKQVQRCAEDRTVLITTYEGNHNH

>Gm135

EILDDGFKWRKYGKKMVKNSPNPRNYYRCSVDGCQVKKRVERDKDDPRYVITTYEGIHNH

>Gm136

LYVRDGYQWRKYGQKVTRDNPSPRAYFKCSYAPSCPVKKKVQRSVEDPSVLVTTYEGEHNH

>Gm137

LMVKDGYQWKKYGQKKVTKDNPSPRAYFKCSLAPSCPVKKRVQRSIQDKSILVATYEGKHNH

>Gm138

LYVMDGYQWRKYGQKVTRDNPSPRAYFRCSSAPSCPVKKKVQRSLEDPTILVTTYEGEHNH

>Gm139

NLSSDLWAWRKYGQKPIKGSPYPRNYYRCSSCKGCAARKQVERSTSEPNTFIVTYTGDHKH

>Gm140

SMISDGCQWRKYGQKMAKGNPCPRSYYRCSMGTACPVRKQVQRSAEDQSVLITTYEGQHNH

>Gm141

DVLDDGYKWRKYGQKVVKNSLHPRSYYRCTHNNCRVKKRVERLSEDCRMVITTYEGRHNH

>Gm142

DVLDDGYKWRKYGQKVVKNSLHPRSYYRCTHNNCRVKKRVERLSEDCRMVITTYEGRHNH

>Gm143

NLSSDIWAWRKYGQKPIKGSPYPRGYYRCSSSKGCLARKQVERNRSDPAMFIVTYTGEHNH

>Gm144

NLSSDLWAWRKYGQKPIKGSPYPRNYYRCSSCKGCVARKQVERSTTEPNTFIVTYTGDHKH

>Gm145

GPLDDGYSWRKYGQKDILGALYPRGYYRCTHRNVQGCMATKQVQRSDEDPTIFEITYRGKHTC

>Gm146

DVLDDGYQWRKYGKKIVKNNKFPRSYYRCSHQDCNVKKQIQRHSRDEQIVVTTYEGTHTH

>Gm147

SSMFDGCQWRKYGQKTAKGNPCPRAYYRCSMGTACPVRKQVQRCFKDETVLITTYEGNHNH

>Gm148

ILIEDGYVWRKYGQKMTLNAKYLRSYYRCSHKNDQGCQAMKQVQRIQDNPPLYRTTYYGHHTC

>Gm149

GPLDDGYSWRKYGQKDILGALYPRGYYRCTHRNVQGCMATKQVQRSDEDPTIFEITYRGKHTC

>Gm150

DILDDGYRWRKYGQKAVKNNMHPRSYYRCTHHTCNVKKQVQRLSKDTSIVVTTYEGIHNH

>Gm151

DVLDDGYKWRKYGQKVVKNTQHPRSYYRCTQDNCRVKKRVERLAEDPRMVITTYEGRHVH

>Gm152

EIDDDGYRWRKYGQKIMKGCLYPRAYYKCASAGCFVRKHVERDSRNNKNVIITYDGRHNH

>Gm153

TILDDGYNWRKYGQKVIKGHTYPRAYYKCISAGCYVRKHVERDSCNRKIVINTYEGKHNH

>Gm154

DHLDDGYKWRKYGQKAVKNSPYPRSYYRCTSAGCGVKKRVERSSDDPSIVVTTYEGQHRH

>Gm155

VMGDDGYKWRKYGQKSIKNSPNPRSYYRCTNPRCSAKKQVERSNEDPDTLIITYEGLHLH

>Gm156

VVPSDLWAWRKYGQKPIKGSPYPRGYYRCSSSKGCSARKQVERSRNDPNMLVITYTSEHNH

>Gm157

VVPSDLWAWRKYGQKPIKGSPYPRGYYRCSSSKGCSARKQVERSRNDPNMLVITYTSEHNH

>Gm158

DILDDGYCWRKYGQKVVRGNPNPRSYYKCTNAGCPVRKHVERASHDPKAVITTYEGKHNH

>Gm159

NLSADLWAWRKYGQKPIKGSPYPRNYYRCSSSKGCMARKQVERSNTEPDMFIVTYSGDHSH

>Gm160

VPPEDGYTWRKYGQKEILGSKYPRSYYRCTHQKLYECQAKKQVQRLDHNPNIFEVTYRGNHTC

>Gm161

DGVSDPWAWRKYGQKPIKGSPYPRSYYRCSSSKGCLARKHVERSHLDPGVFIVTYTAEHSDPHPTC

>Gm162

GSLDDGYSWRKYGQKDILRAKFPRGYYRCTHRNVQGCLATKQVQRSDEDPTTIEVTYRGRHTC

>Gm163

TPKLDGHQWRKYGQKEILNAKYSRNYYRCTHKYDQNCQAIKQVQRIQEDPPLYKTTYLGHHTC

>Gm164

SMIADGCQWRKYGQKMAKGNPWPRAYYRCTMSTGCPVRKQVQRCAEDRSVLITTYEGQHNH

>Gm165

MPPEDGFTWRKYGQKEILGSKFPRSYYRCTHQKLYECQAKKQVQRLDQNPNIFEVTYRGDHTC

>Gm166

ILIEDGYTWRKYGQKMTSQSKYLRSYYRCTHKNDQGCQAIKQVQRIQDNPPLYRTTYYSHHTC

>Gm167

ILLEDGYAWRKYGQKITLNAKYLRSYYRCTHKYDQGCPATKQVQRTQEDPPLYRTTYYGHHNC

>Gm168

ILMEDGYAWRKYGQKMTMNAKYLRNYYRCTHKYDQGCLATKQVQRIQEDPPLYHTTYYGHHNC

>Gm169

RMVGDGYNWRKYEDKVVKGSANQLSYYKCTQPTCYVKKKVERTIEGEIVDIHYQGTHTH

>Gm170

NLSSDIWAWRKYGQKPIKGSPYPRGYYRCSSSKGCLARKQVERNRSDPTMFIVTYTAEHNH

>Gm171

CTTDDNHAWRKYGQKRILNSEFPRSYFRCSHKYDQGCRAIKQVQVDQENPNMLQTTYIGIHTC

>Gm172

CTIDDNHVWRKYGQKRVLNTEFPRSYFRCGYKYDQGCKANKQVQRDQENPNMYRITYIGIHTC

>Gm173

CTTDDNHVWRKYGQKGVLNSEFPRSYFRCGHKYDQGCLAKKQVQRDQENSNMYRTTYIGIHTC

>Gm174

TPKVDGHQWRKYGQKEILKAKYSRSYYRCTHKYDQNCQATKQVQRIQEDPPLYKTTYLSHHTC

>Gm175

DIPPDEYSWRKYGQKPIKGSPYPRGYYKCSTIRGCPARKHVERAPDDPAMLIVTYEGEHRH

>Gm177

VVPSDLWAWRKYGQKPIKGSPYPRGYYRCSSSKGCPARKQVERSRTDPNMLVITYTSEHNH

>Osj01

PIIADGCQWRKYGQKMAKGNPCPRAYYRCTMATGCPVRKQVQRCAEDRSILITTYEGTHNH

>Osj02

VVPSDLWAWRKYGQKPIKGSPYPRGYYRCSSSKGCSARKQVERSRADPTMLVVTYTSDHNH

>Osj03

DHLEDGYRWRKYGQKAVKNSPFPRSYYRCTNSKCTVKKRVERSSDDPSVVITTYEGQHCH

>Osj04 N

QPAKDGYNWRKYGQKQLKDAESPRSYYKCTRDGCPVKKIVERSSDGCIKEITYKGRHSH

>Osj04 C

DLLDDGYRWRKYGQKVVKGNPRPRSYYKCTADGCNVRKQIERASADPKCVLTTYTGRHNH

>Osj05

PMISDGCQWRKYGQKMAKGNPCPRAYYRCTMASQCPVRKQVQRCAEDKSILITTYEGTHNH

>Osj06

DIPSDNYSWRKYGQKPIKGSPHPRGYYRCSSKKDCPARKHVERCRSDPAMLLVTYENEHNH

>Osj07

EILDDGYKWRKYGKKSVKNSPNPRNYYRCSTEGCNVKKRVERDKDDPSYVVTTYEGTHNH

>Osj08

DHLEDGYRWRKYGQKAVKNSSYPRSYYRCTAPRCGVKKRVERSEQDPSMVITTYEGQHTH

>Osj09

PMISDGCQWRKYGQKMAKGNPCPRAYYRCTMAIGCPVRKQVQRCAEDKTVLITTYEGNHNH

>Osj10

EVLDDGFKWRKYGKKAVKSSPNPRNYYRCSAAGCGVKKRVERDGDDPRYVVTTYDGVHNH

>Osj11

DHLEDGYRWRKYGQKAVKNSPYPRSYYRCTTPKCGVKKRVERSYQDPSTVITTYEGQHTH

>Osj12

GVSSDLWAWRKYGQKPIKGSPYPRGYYKCSSMKGCMARKMVERSPAKPGMLVVTYMAEHCH

>Osj13

PPPSDSWAWRKYGQKPIKGSPYPRGYYRCSSSKGCPARKQVERSRADPTVLLVTYSFEHNH

>Osj14

NTPTDSWAWRKYGQKPIKGSPFPRAYYRCSSSKGCPARKQVERSRNDPDTVIVTYSFEHNH

>Osj15

GPLDDGFSWRKYGQKDILGAKYPRAYFRCTHRHTQGCHASKQVQRADGDPLLFDVVYHGDHTC

>Osj16

DHLEDGYRWRKYGQKAVKNSPYPRSYYRCTTQKCPVKKRVERSYQDPAVVITTYEGKHTH

>Osj17

GLADDGYKWRKYGQKSIKNSPNPRSYYRCTNPRCNAKKQVERAVDEPDTLIVTYEGLHLH

>Osj18

TPHFDGHLWRKYGQKNIKDSAFPRLYYRCSYREDRQCLASKLVQQENDDDPPLYRVTYTYEHTC

>Osj19

ASLDDGLSWRKYGQKDILGAKYPRAYFRCTHRHTQGCNATKQVQRADGDPLLFDVVYLGDHTC

>Osj20

SPYKDGYQWRKYGQKNIQDSNYLRLYFKCTFSRERSCAAKKQVQQRDAGEPPMFLVTYLNEHTC

>Osj21

GTTTDGFIWRKYGQKEINGCKHPRLYYRCAFRGQGCLATRRVQQSQSQDDPAAAFVIAYYGEHTC

>Osj22

APYHDGYQWRKYGQKMIRGNSFPRCYYRCTYHQDHGCPASKHVEQHNSEDPPLFRVIYTNEHTC

>Osj23

DILDDGYRWRKYGQKAVKNSKHPRSYYRCTHHTCNVKKQVQRLAKDTSIVVTTYEGVHNH

>Osj24 N

RSSDDGYNWRKYGQKQVKGSENPRSYYKCTFPNCPTKKKVERSLDGQITEIVYKGTHNH

>Osj24 C

DILDDGYRWRKYGQKVVKGNPNPRSYYKCTTAGCPVRKHVERASHDLRAVITTYEGKHNH

>Osj25

DIPADEYSWRKYGQKPIKGSPYPRGYYRCSTVKGCPARKHVERAADDPATLVVTYEGDHRH

>Osj26

EILDDGYKWRKYGKKSVKNSPNPRNYYRCSTEGCNVKKRVERDKNDPRYVVTMYEGIHNH

>Osj27

PTVKDGCQWRKYGQKTAKGNPWPRGYYRCTGAPGCPVKKQVQRCNHDTSVLVTTYDGVHNH

>Osj28

LVVKDGYQWRKYGQKVTKDNPCPRAYFRCSFAPACPVKKKVQRSADDNTVLVATYEGEHNH

>Osj29

DHLEDGYRWRKYGQKAVKNSPFPRSYYRCTNSKCTVKKRVERSSDDPSVVITTYEGQHSH

>Osj30 N

TPAEDGYNWRKYGQKQVKNSEHPRSYYKCTFTNCAVKKVERSQDGQITEIVYKGSHNH

>Osj30 C

DILDDGYRWRKYGQKVVKGNPNPRSYYKCTHPGCSVRKHVERSSHDLKSVITTYEGKHNH

>Osj31

GPAPDLWAWRKYGQKPIKGSPYPRGYYRCSSNKNCAARKQVERCRFDPSFLLLTYTGAHSGH

>Osj32

PTMNDGCQWRKYGQKVAKGNPCPRAYYRCTVAPGCPVRKQVQRCLEDMSILVTTYEGTHNH

>Osj34

DVLDDGYKWRKYGQKVVKNSLHPRSYFRCTHSNCRVKKRVERLSTDCRMVITTYEGRHTH

>Osj35 N

AVAEDGYSWRKYGQKQVKHSEYPRSYYKCTHASCAVKKKVERSHEGHVTEIIYKGTHNH

>Osj35 C

DILDDGYRWRKYGQKVVKGNPNPRSYYKCTHPGCLVRKHVERASHDLKSVITTYEGKHNH

>Osj36

DVLDDGYKWRKYGQKVVKNSLHPRSYYRCTHNNCRVKKRVERLSEDCRMVITTYEGRHTH

>Osj37

VVPSDLWAWRKYGQKPIKGSPYPRGYYRCSSSKGCSARKQVERSRTDPNMLVITYTSEHNH

>Osj39

GVSADVWAWRKYGQKPIKGSPYPRGYYRCSSSKGCPARKQVERSRSDPNTFILTYTGEHNH

>Osj40

VPHYDGHQWRKYGQKHINNSKHPRSYYRCTYRQEEKCKATKTVQQREDLHHANSYNGDHPIMYTVVYYGQHTC

>Osj41 N

nteddglswskyeqkeilgakfprayfrcthwntkkgcmatkevqrddgdplmfdivyhgehtc

>Osj41 C

vpaddgyswrkygqknvlgfsylrgyyrcatkgcqaskqvqrhddgllfdvtyfgehtc

>Osj42

DIPADDYSWRKYGQKPIKGSPYPRGYYKCSTVRGCPARKHVERDPGEPAMLIVTYDGDHRH

>Osj43

PMISDGCQWRKYGQKMAKGNPCPRAYYRCTMAAGCPVRKQVQRCAEDRTVLITTYEGNHNH

>Osj44

ATLEDGHVWRKYGQKDIQNSPYPRSYYRCTHKLDQGCGARRQTQRCEADPSNYDITYYGEHTC

>Osj45

KNLDDGQAWRKYGQKEIQNSKHPKAYFRCTHKYDQLCTAQRQVQRCDDDPASYRVTYIGEHTC

>Osj46a

APYDDGHQWRKYGEKKLSNSNFPRFYYRCTYKNDMKCPATKQVQQKDTNDPPLFSVTYFNHHTC

>Osj46b

APYDDGHQWRKYGEKKLSNSNFPRFYYRCTYKNDMKCPATKQVQQKDTNDPPLFSVTYFNHHTC

>Osj47

KELEDGRQWRKYGQKHIQDSPNNPRSYYRCTHRPDQGCMATKQVQTSESNSSEFVISYYGEHTC

>Osj48

RTSEDGFLWRKYGQKEIKNSKHPRLYYRCSYKDDHGCTATKQVQQSEEDPSLYVITYFGDHTC

>Osj49

DHLEDGYRWRKYGQKAVKNSPFPRSYYRCTTQKCPVKKRVERSYQDAAVVITTYEGKHTH

>Osj50

VPDFDGYQWRKYGQKQIEGALYPRSYYRCTNSTNQGCLAKKTVQRNGGGGAAGYTVAYISEHTC

>Osj51

DIPADDFSWRKYGQKPIKGSPFPRGYYKCSTLRGCPARKHVERDPTDPSMLIVTYEGEHRH

>Osj52

VPHEDGFQWRKYGEKKIQGTHFTRSYFRCTYRDDRGCQATKQIQQKDKNDPPMFQVTYSNEHTC

>Osj53 N

NKLEDGYNWRKYGQKQVKGSENPRSYYKCTYNGCSMKKKVERSLADGRITQIVYKGAHNH

>Osj53 C

DILDDGFRWRKYGQKVVKGNPNPRSYYKCTTVGCPVRKHVERASHDTRAVITTYEGKHNH

>Osj54

ATMDDKFLWRKYGQKEIKNSKHPRFYYRCSYKDDHGCTATKQVQQSETADDDTASPVYIITYFGEHTC

>Osj55

APHNDGHQWRKYGEKKINNCNFPRYYYRCTYKDNMNCPATKQIQQKDYSDPPLYSVTYYNEHTC

>Osj56

APHNDGHQWRKYGQKWISRAKHSRSYYRCANSKVQGCPATKTVQQMDSSGNGTSKLFNVDYYGQHTC

>Osj57

SLSYDGYSWRKYGQKQVKGSEFPRSYYKCTHPTCPVKRKVEMTPDGRIAEIVYNGEHNH

>Osj58

DILDDGYRWRKYGQKAVKNSDFPRSYYRCTHHTCNVKKQVQRLAKDRGIVVTTYEGVHNH

>Osj60

VMPEDGYEWKKYGQKFIKNIQKNRSYFRCRDQRCGAKKKVEWHPHDPGLNLRVVYDGAHHH

>Osj61

GPPDDGYSWKRYGQKNIFGANYPRCYYRCIHKTTTGCTATKNAQATDGDPLLFDVVYHGEHTC

>Osj62

ETVKDGYQWRKYGQKVTRDNPYPRAYFRCAFAPSCPVKKKLQRCAEDRSMLVATYEGEHNH

>Osj64

VPHYDGHQWRKYGQKHINNSKHPRSYYRCTYRQEEKCKATKTVQQREDLHHANSYNGDHPVMYTVVYYGHTC

>Osj65

VPDFDGYQWRKYGQKQIEGAMYPRSYYRCTNSTNQGCLAKKTVQRNGGGGAAGYTVAYISEHTC

>Osj66

VVPSDLWAWRKYGQKPIKGSPYPRGYYRCSSSKGCSARKQVERSRTDPNMLVITYTSEHNH

>Osj67

EILDDGFKWRKYGKKAVKNSPNPRNYYRCSTEGCNVKKRVERDREDHRYVITTYDGVHNH

>Osj68

DIPPDEYSWRKYGQKPIKGSPYPRGYYKCSTVRGCPARKHVERATDDPAMLVVTYEGEHRH

>Osj69

GPADDGHSWRKYGQKDILGAKHPRGYYRCTHRNTQGCTATKQVQRTDDDASLFDVVYHGEHTC

>Osj70 N

RSSDDGYNWRKYGQKQMKGSENPRSYYKCTFPGCPTKKKVEQSPDGQVTEIVYKGAHSH

>Osj70 C

DILDDGYRWRKYGQKVVKGNPNPRSYYKCTTAGCPVRKHVERASNDLRAVITTYEGKHNH

>Osj71

LVVKDGYQWRKYGQKVTKDNPCPRAYFRCSFAPACPVKKKVQRSAEDNTILVATYEGEHNH

>Osj72

DILDDGYRWRKYGQKAVKNNKFPRSYYRCTHQGCNVKKQVQRLSRDETVVVTTYEGTHTH

>Osj73

DILDDGYRWRKYGQKAVKNNKFPRSYYRCTHQGCNVKKQVQRLSRDETVVVTTYEGTHTH

>Osj74

PPVDDGHSWRKYGQKEILGAKHPRGYYRCTHRHSQGCMATKQVQRTDEDATVFDVIYHGEHTC

>Osj75

QPPDDGYTWRKYGQKDILGSRYPRSYYRCTHKNYYGCEAKKKVQRLDDDPFTYEVTYCGNHTC

>Osj76

LVVKDGYQWRKYGQKVTRDNPSPRAYFRCAFAPSCPVKKKVQRSAEDSSLLVATYEGEHNH

>Osj77

DVLDDGYRWRKYGKKMVKNSPNPRNYYRCSSEGCRVKKRVERARDDARFVVTTYDGVHNH

>Osj78 N

KSAEDGYNWRKYGQKHVKGSENPRSYYKCTHPNCDVKKLLERSLDGQITEVVYKGRHNH

>Osj78 C

DILDDGYRWRKYGQKVVKGNPNPRSYYKCTNTGCPVRKHVERASHDPKSVITTYEGKHNH

>Osj79

DVLDDGYKWRKYGQKVVKNTQHPRSYYRCTQDNCRVKKRVERLAEDPRMVITTYEGRHVH

>Osj80

DILDDGYRWRKYGQKVVKGNPNPRSYYKCTHQGCSVRKHVERASHDLKSVITTYEGKHNH

>Osj81

VPHEDGYQWRKYGEKKIQGTHFTRSYFRCTYRDDRGCQATKQIQQEDKNDPPMFQVTYSNEHTC

>Osj82 N

NMVGDGFNWRKYGQKQVKSSENSRSYYRCTNSNCLAKKKVEHCPDGRVVEIIYRGTHNH

>Osj82 C

GKTSDGYRWRKYGQKIVKGNPNPRSYYRCTHDGCPVRKHVEKAPDDDNNIVVTYEGKHNH

>Osj83

DIPPDEYSWRKYGQKPIKGSPHPRGYYKCSSVRGCPARKHVERCVDDPAMLIVTYEGEHNH

>Osj84 N

KPADDGYNWRKYGQKAVKGGEYPRSYYKCTHLSCPVKKKVERSSDGQITQILYRGQHNH

>Osj84 C

DLLDDGYRWRKYGQKVVKGNPHPRSYYKCTYQGCDVKKHIERSSQDPKAVITTYEGKHSH

>Osj85 N

KPADDGYNWRKYGQKVVKGSDCPRSYYKCTHPNCPVKKKVEHAEDGQISEIIYKGKHNH

>Osj85 C

DLLDDGYRWRKYGQKVVKGNPHPRSYYKCTYAGCNVRKHIERASSDPKAVITTYEGKHNH

>Osj86

SPYDDGYQWRKYGQKKINNTNFPRSYYRCSYHRERRCPAQKHVQQRDGDDVPALHVVVYTHEH

>Osj87

DIPADEFSWRKYGQKPIKGSPHPRGYYKCSSVRGCPARKHVERCVDDPSMLIVTYEGDHNH

>Osj88

VIPSDLWAWRKYGQKPIKGSPYPRGYYRCSSSKGCMARKQVERSRSDPNMLVITYAAEHNH

>Osj89

GPPADSWSWRKYGQKPIKGSPHPRGYYKCSSYRGCPARKQVDKCRNDASLLIITYTSDHNH

>Osj90

VPHYDGHQWRKYGQKNINNSNHQRSYYRCSYKHEQNCKATKTVQQLDSAGETIMYTVVYYGQHTC

>Osj91

TPENDGFHWRKYGEKNILNSEFRKLYYRCGYSDERKCQAKKYVQQENNKHPPEFRVTLTNEHTC

>Osj92

GVSTDLWAWRKYGQKPIKGSPYPRGYYKCSSLKACMARKMVERSPEKPGVLVITYIAEHCH

>Osj93

ATPDDGFSWRKYGQKDILGAKFPRGYYRCTYRNAQGCPATKQVQRSDADLAVFDVTYQGAHTC

>Osj95

GDRDDSYPWRKYGQKDILGARFARSYYRCAQMLGCTARKQVQQSDDDPSRLEITYIGLHTC

>Osj96 N

RPTDDGYNWRKYGQKAVKGGEYPKSYYKCTHLNCLVRKNVEHSADGRIVQIIYRGQHTH

>Osj96 C

DLLDDGYRWRKYGQKVVKGNPYPRSYYKCTYLGCDVKKQVERSVEEPNAVITTYEGKHIH

>Osj97

PTMNDGCQWRKYGQKISKGNPCPRAYYRCTVAPNCPVRKQVQRCADDMSILITTYEGTHSH

>Osj98

TPDYDGYEWRKYGQKSISKTKHSRSYYRCTNQKGQGCMATKTVQQIENDNSSNSVVKLYNVDYFGKHTC

>Osj99

IPHTDGHLWRKYGEKKIKNSSFPRRWCKTVAIYRLYYRCSYRDDRNCMATKVVQQENDADPPLYRVTYIHPHTC

>Osj100

APHNDGHQWRKYGQKWISRAKHSRSYYRCANSKVQGCPATKTVQQMDSSGNGTSKLFNVDYYGQHTC

>Osj101

KMPADGYKWRKYGQKSIKNNPHPRSYYKCTSSRCSAKKHVEKSTDDPEMLIVTYEGSHHH

>Osj102

SLSYDGYSWRKYGQKQVKGSEFPRSYYKCTHPTCPVKRKVEMTPDGRIAEIVYNGEHNH

>kfl00096_0150 N
KPSEDGYNWRKYGQKQVKGSQYPRSYYKCTHPGCDVKKKVERSKDGKVAENVYKGTHTH

>kfl00096_0150 C
DILDDGFRWRKYGQKQVKDNSHPRSYYKCTSANCPVRKHVERSATDIRAVITTYEGKHNH

>kfl00189_0180
ITIFDGCNWRKYGQKIAKGNPCPRAYYRCTQAAGCPVRKQVQRSADDPSVLVVTYEHMHNH
