# Supplementary material for: The evolution of WRKY transcription factors
Source: BMC Plant Biol. 2015 Feb 27;15:66. doi: 10.1186/s12870-015-0456-y (PMC4350883; doi:10.1186/s12870-015-0456-y)
Supplement: Additional file 3: Table S2. — All R protein-WRKYs used for phylogenetic analysis in fasta format. [file 12870_2015_456_MOESM3_ESM.docx]

>SORBIDRAFT_02g008880

mllsdeadns sdqivtsaci vgesgmgkte lvhqiynnrm ifdtfdlriw lnmcdkkrll

gkivelttca scsdasisvl eeivieelts krlllvldds eiksqyfwgy irkllnvcak

gsavivttks kevanqigam qtfylsplsk eecfmifkeh vledlamnny cqlesigwkf

vekcggnpmc ikvlsgllch seiglseidm ivdgilpalr lcydllpahl qqcfkfcslf

pkdyifvkhh iirlwiaegf vfceegtkpe dtalhyfdql fcrsffqrsp fhsdhkdsfv

mhelfhdlah svsknecfrc eepfcsfaen vshlslvlsd fktaalsnev rnlqsflvvr

rclpvvrift lddifvkhrf lralnlsytd ilelpisign mkhlrllaln ntkikslpie

igqvnslqtl elkdcchlid lpgstsslak lrhldvqkew gnvnvgmphg igyltdlqtl

ttfnigndll hcsiselknl nglsghvhvt gleniktand areanmmgkh llealtlews

yqeegmdddm gkeianeilq hlqpnsnime lviqnyagnl fpvwmqdnyl cklisvtldn

chgcselpyl gdlpslkslf iqringverf gietsslate ekhptgfpsl evlnicemyd

lqfwvsmreg dfprlfrlsi srcpkltnlp rlislvhvsf yygvelptfs elpsleslki

egfqkirsin fphhlttlkk leiidckell svyahslsvs dlkvvrclkl dlvgsstedh

igqkvvngrn sltrrpmvlk tltytnvldd dskwekfgek nifgyifars yyrciwrnlt

gcsatkilqp dnndantlfv myisehnhgf snephlelqs qlgtgrkrke ydvssdents

rkqlhdssvs aassvprpat geatpssaee lkakkmgrpr kpnprvfgpe wsia

> Os07g0273700

mepgsssreg eqqepanerl tatggggggg ggspvsdqel sdgeevsdge yqagddfsgy

avrgrgfvek ehifdkvvtp sdvgnlgrlv ipwqhaecyf prdvpanere gvvlrfedda

gnswrflyrg ssltlgwshf frknrldagd mvsfyrgase atrdrlfihs krrmhilptl

gysdpqvhin rlfqllvrvr tmvssfgksg spsppnsplp tvqnftykal kdclqdlhyy

sllsmmgrnh ksqstsakir dtiptldali qlrslsdpvf ripaaatshc illcrgvlgi

mgflcsdenm kedhrmlqvp afddlnysaq dkitrmkeqt mpssladpiy llptairnll

yldlsncsdi vqlppslgss lhmlsalnls ccyslralpd slvclydlqi lllsfchnlq

nlpvsfgdls nlrlldlsgc rslrlfpssf vnlgslenln lsdcirlmgi pqnfedlqkl

eylnfagcyr vdlpvycltn lvnlkcltls nhtdikdfpy sftdlkrhly lsrwwkynwv

htqcnlksyr chqqriinsl lsdgsdegdi tseqsltsic ifgergtgkt ellheiyndq

kilegfhlri winmcdkkrl lekiieftac aycydapssi leetvreeln gkrfllvlnd

adienqcfwt dvwkvsnvga agsalivttr skevaslfga mkpyymnpls keecfmvfqe

hadcgfdinn dheltkvgwk ivekcggnll cmkalsgllw hsktalseid slvggivpal

rlcydllpsh lkqcfkfcsl fpkdyvfvkh hiiqlwisqg fvypeedsqp edtglqyfne

flcrsffqhc pfsndhedkf vmhelfhdla rsvskdesfs seepffslpe nichlslvis

dsntvvltke hrhlqslmvv rrsateysss fvpllkilgl ndllmkcgfl ralnlsctti

vdlpgsigrm khlrflamnn tkikslptei gqlntlqtle lkdcccliel pestknlmkl

rhldvqkepg nihvgmpsgl gqltdlqtlt vfnigddlsh csirdlknls glrghvhitg

lqnitagdda keanlvgkqf lqaltlewcc sseemedesd keianqvlqn lqpntsiqel

aiqnypgnsf pnwikdsglc mlvsitidns qdcneipylg dlpclkflfi qkmyavenfg

qrsnslttdg khapgfpsle ilnlwemysl qfwngtrygd fpqlrglsis rcpklsnlpp

lisllylsfh cgdqlpalse fpslkslkie gfqklksvsf cpempllqkl eisdckedkc

tikyllvntm kddgwnwiky gqkdiigsky qrsylrcnqm hstgckarki vepsnddlni

wlvtyiyehn hqqragptde plpsgqhvql attrkrkefd asndemtpkk qfrnmcvsss

pgsskdvkqr

> contig22731/9-CL19939Contig1

MEVEPGSGGSSRCRAAEGELQDEQGDASVEVQLDLFPGPAPRPLRVKESSSSDSGITFSV

GSQSSKKGGGGSRSRGDRAPPATAVGKQHLLDKVLTPADVGERLVIPEEDAVCWPFLPGI

VFTFEDPNGKQWSFRCSYSEELRGCYMTKEWSRFVREKGLHAGDTVSFYRGLGAAGHGRF

FVDWKLHADGAHRRPKQPPMATPNLELADPCSASSIRRLSQQLVHVRTTISPSTSSGTQV

SFAWLTNVRSLAADAEDCLQDLHCRMMQAVLVVGSHEKDPNVSGNSSKATSSSFFQSASP

LQLTVKSIGDKLEGLQHGRPELSVAAPHNSSKLISVNHLVVRCILLCRGILGIMSAAGAD

EVKATREIPVFASDDLTYAGRANVSMMDSPKMPANVTDPIYLLPTFIRSLLYLDLSNCSS

LAQVHPSLCTLHHLAALNLSRCYSLRTLPVSLGMLQKLQILVLSNCQKLQNLPVSLCDLS

KLRLLDLSGCSRLETLPYSFVTLRQLEILNLSYCKGLKELPQPFGILQGLKYLNLSGCHG

LDLDVECKLANLMCATLSPHSNIQGFADSFRDLKNHLDMSRWRKKSRVHPQCNPKAVPFH

TYKCHEQSIIERLLSVNFDESVVTGDHVVISICVVGESGMGKTDLIHRIYNDKLILDTFD

LRIWLPACDKKMLLGKIVEFTTFSYCCDSPMSVLEEILTEELTGKKFLLVLDDCDNESPQ

FWNELLKLFNVCSKGSAVITTTKNKEVANHMGATQTYYLSSLPKEDCFMIFHRHALGSLD

MKSYPPLESVGWKVVEKCGEIGLSQMDMLVDGILPALRLCYDLLPSHLQQCFKFCSLFPK

DYNFSKHHIVRLWIAQGFVFPEEGCQPEETGLHYFDELFCRSFFQHSPFHNDNEDKFVMH

ELFHDLAQSVSRNECFRSEEPFCSFPENISHLSLVPSDFKTVALIKEIKNLQSLLVVRRF

FPVVRVLHGDLYVKYRFLRALNLSSTDILQLPSSIRNMKYLRFLALNNTKIKALPFEIGQ

VDTLQTLELKDCCRLTELPGSTSNLTKLRHLDVRQEPGNIRVSMPHGIGQLTDLQTLTVF

NIGNNLLHSSIVELKNLSGLLGHVHITGLENIKTADDAREANIVGKHFLEALTLEWSYSE

GDMDDEMGEVIANDILQNLQPNRNIMELVVRNYAGNQFPAWMQDSYLCNLVSVTLDNCHE

CSELPYLGDLPCLKSLFIQRMNSVESFGIESNSLAIEENHSLRFPSLEVLTLREMYDLQF

WVGITEGDFPRICHLSISRCPKLTELPPLLSLVHLSIHCGGQVPSFSELPSLESLKIEGF

NKIRSIIFPHQLTTLKKLEISDCKELSSMNSYSLSVSNLRVVRCPKLDLVGSSLEDHHRQ

QVDSGRNIPTRWSMVLKTATYTDLQVDSWKWEKHGEKNIFGSNLARSYYRCLHRNSTGCC

ATKILQPNDTDPNMLSAMYIYEHNHEFPNEPHLELSAEPATTRKRKEPDVPSDEHTSKRQ

LNS

>SLH1 [Arabidopsis thaliana]

mtncekdeef vciscveevr ysfvshlsea lrrkginnvv vdvdiddllf kesqakieka

gvsvmvlpgn cdpsevwldk fakvlecqrn nkdqavvsvl ygdsllrdqw lseldfrgls

rihqsrkecs dsilveeivr dvyethfyvg rigiysklle ienmvnkqpi gircvgiwgm

pgigkttlak avfdqmssaf dascfiedyd ksihekglyc lleeqllpgn datimklssl

rdrlnskrvl vvlddvcnal vaesflegfd wlgpgsliii tsrdkqvfrl cginqiyevq

glnekearql fllsasimed mgeqnlhels vrvisyangn plaisvygre lkgkkklsem

etaflklkrr ppfkivdafk ssydtlsdne knifldiacf fqgenvnyvi qllegcgffp

hveidvlvdk clvtisenrv wlhkltqdig reiingetvq ierrrrlwep wsikylleyn

ehkangepkt tfkraqgsee ieglfldtsn lrfdlqpsaf knmlnlrllk iycsnpevhp

vinfptgslh slpnelrllh wenyplkslp qnfdprhlve inmpysqlqk lwggtknlem

lrtirlchsq hlvdiddllk aenlevidlq gctrlqnfpa agrllrlrvv nlsgcikiks

vleippniek lhlqgtgila lpvstvkpnh relvnfltei pglseaskle rltsllesns

scqdlgklic lelkdcsclq slpnmanldl nvldlsgcss lnsiqgfprf lkqlylggta

irevpqlpqs leilnahgsc lrslpnmanl eflkvldlsg cseletiqgf prnlkelyfa

gttlrevpql plslevlnah gsdseklpmh ykfnnffdls qqvvndfflk altyvkhipr

gytqelinka ptfsfsapsh tnqnatfdlq pgssvmtrln hswrntlvgf gmlvevafpe

dycdatdvgi scvcrwsnke grscriernf hcwapgkvvp kvrkdhtfvf sdvnmrpstg

egndpdiwag lvvfeffpin qqtkclndrf tvtrcgvrvi nvatgntsle nislvlsldp

vevsgyevlr vsyddlqemd kvlflyiasl fndedvdfva pliagidldv ssglkvladv

slisvssnge ivmhslqrqm gkeilhgqsm llsdcessmt enlsdvpkke kkhreskvkk

vvsipaideg dlwtwrkygq kdilgsrfpr gyyrcaykft hgckatkqvq rsetdsnmla

itylsehnhp rptkrkalad strstsssic saittsassr vfqnkdepnq phlpssstpp

rnaavlfkmt dmeefqdnme vdndvvdtrt lalfpefqhq peeedpwstf fddynfyf

>CARUB_v10025742mg [Capsella rubella]

mtncendagf vciscvdevr csfvshlsea lrrkginyav vdvssdelfs kesrekveka

rvsvivlpgn cepsrvcldn famvlecqrr mvvpvlygds plreewlsel dlkglspvhk

srkecsdstl veeivgdvye klfysgrigi ysklleiekl vgnqpfgirc vgiwgmpgig

kttlakavfd qmsgafdasc fiedyeksih ekglycllee hllkespgnd atimnlsslr

drlnskrvlv vlddvrnglv aesflegfdw lepgsliiit srdkqvfrlc qinqiyevqg

lnerealqlf llcaslkdmg eqnlrefslk vinyangnpl ainvygrelk gkkklsemet

vflkvkrrpp fkivdafkss ydtlsdnekn ifldiacffh genvnyviql legcgffphv

gidvlveksl vtvsenrvrl hnltqavgqe iingetvqie rrkrlwepws ikylleynep

kaneepkttf kraqgseeie gmfldasnlk fdvqpsafkn mlnlrllkiy csnpevhpvi

nfpkdffhsl pdelrllhwe nyplqslpqs fdprhlvein mpysqlqklw ggtknlealr

tvrlchsqhl vdiddlvkaq nlevidlqgc trlqnfpaag qllhlrvvnl sgcieiksfl

eippnietlh lqgtgilalp lstvkpnhre llnflteips lsealklerl tsllecrtsc

qdlgklicle lkdcsclqsl pnlanldlln vldlsgcsrl nsiqgfprfl kelyltgtai

revpqlpqsl ellnahgsiv qslpdmanle flkvldlsgc seletvqgfp rnlkelylag

ttlrevpqlp lslellnahg svslksirpn yyklpmhytf snlfdlspqv vndflvkalt

nvkhisrkym qnlnkaptfs fsapshanqn aplglqpgss vmtrlnpywr nmlvgfgmmv

evafsedycd atgfgiscvc rwsnkegrsy kiernfhcwp pgkvvpkvlk nhtfvfcdin

mspstdggnd pgiwadlvvf effpinqqtk slidrftvtr cgirvidvtt gytslknisl

vlslnpmevs gyevveevlr vsyddlqemd kvlflyiacl fndedvdvva pliagidlav

ssglkvladv slisvssnge ivmhsllrkm akeilhgqai vlsdcestma dnlsdipkkr

rkrnikkvvc atanedlwsw rkygkkeilg slfprsyfrc thkfaqgcka tkqvqrsdtd

pnmftityls ehnhpsstew malagpsrpt rstsssnysa vttsassrvs qnkvksnklh

lpssstppgn agvqlkekdm eefqdnmeld ndvedictle lfpefqhqpe enpssstfdn

kdwsdwfsmf sipkfqdqpe egpfspdwlw e

>XP_002865255 Arabidopsis lyrata

mtncekaeel vciscvdevr ysfvshlsea lrrkginnvv ievegddlls keseakieka

rvsvmilsri ceptrachkf ekvrecqrnk nqvvvpvlyg espslldwis vldlkdlsai

hhsrmecsds klvqeivrdv yeklfykgri giysklleie nmvnkqpigi rcvgiwgmpg

igkttlakav fdqmssafda scfiedydka fhekglycll eeqlfkenpg ndatimklss

lrdrlnskrv lvvlddvrna lvaesflegf dwlgpgslii itsrdkqvfr lcginqiyev

qglnekealq lfllcasmge qnlhelsmkv vnyangnpla isvygrelkg kkklsemeta

flklkrrppf kifdafkssy dslcdnekni fldiacffqg envnyviqll egcgffphve

idvlvekclv tisenrvwlh nltqdvgrei ingetvqier rrrlwepwsi kylleynehk

acgepkttfk rtqgsdeieg mfldtsnlrf dvqpsafknm lnlkllkiyc snpevhpvin

fpkgslhslp nelrllhwen yplqslpqsf dpwhlveinm pysqlqklwg gtknlemlrt

irlchsqhlv diddlfkaqn levidlqgct rlqnfpaagq llrlrvvnls gcieiksvle

mppnietlhl qgtgilafpv stvkpnrrel vnflteipgl sealklerlt sllesssscq

dlgkliclel kdcsclqslp nmanldllnl ldlsgcsrln siqgfprflk klylggtaik

evpqlpqsle llnargsclr slpnmanlef lkvldlsgcs eletiqgfpr nlkelyfagt

tlrevpelpl slellnahgs dseklpmhyt fsnffdlspq vvndflvkal tyvkhiprey

tqelnnaptf sfsapshknq nttfglqpgs svitrlntsw rntlvgfgml vevassedyc

datgfgiscv crwsnkegrs criernfhcw apakvvskff pinqqtkcld drfivtrcgv

rvinvatgnt slenislvls ldpvevsgye avkevlrvsy ddlqemdkvl flyisslfnd

edvdlvapli agidldvssg lkvladvsli sissngeivm hclvrqmgke ilheqsmlls

dcessmtenl sdlpkkkksk akkvvcipat deadlwtwrk ygqkyilgsn fprsyyrcty

rftqgclatk qvqrsdtnsn mfaityiseh nhprptkrkv ldgstrstss snysanfcli

>RPS1 A. thaliana

mtncekdeef vciscveevr ysfvshlsea lrrkginnvv vdvdiddllf kesqakieka

gvsvmvlpgn cdpsevwldk fakvlecqrn nkdqavvsvl ygdsllrdqw lseldfrgls

rihqsrkecs dsilveeivr dvyethfyvg rigiysklle ienmvnkqpi gircvgiwgm

pgigkttlak avfdqmssaf dascfiedyd ksihekglyc lleeqllpgn datimklssl

rdrlnskrvl vvlddvrnal vgesflegfd wlgpgsliii tsrdkqvfcl cginqiyevq

glnekearql fllsasiked mgeqnlqels vrvinyangn plaisvygre lkgkkklsem

etaflklkrr ppfkivdafk stydtlsdne knifldiacf fqgenvnyvi qllegcgffp

hveidvlvdk clvtisenrv wlhkltqdig reiingetvq ierrrrlwep wsikylleyn

ehkangepkt tfkraqgsee ieglfldtsn lrfdlqpsaf knmlnlrllk iycsnpevhp

vinfptgslh slpnelrllh wenyplkslp qnfdprhlve inmpysqlqk lwggtknlem

lrtirlchsh hlvdiddllk aenlevidlq gctrlqnfpa agrllrlrvv nlsgcikiks

vleippniek lhlqgtgila lpvstvkpnh relvnfltei pglseelerl tsllesnssc

qdlgklicle lkdcsclqsl pnmanldlnv ldlsgcssln siqgfprflk qlylggtair

evpqlpqsle ilnahgsclr slpnmanlef lkvldlsgcs eletiqgfpr nlkelyfagt

tlrevpqlpl slevlnahgs dseklpmhyk fnnffdlsqq vvndfllktl tyvkhiprgy

tqelinkapt fsfsapshtn qnatfdlqsg ssvmtrlnhs wrntlvgfgm lvevafpedy

cdatdvgisc vcrwsnkegr scrierkfhc wapwqvvpkv rkdhtfvfsd vnmrpstgeg

ndpdiwaglv vfeffpinqq tkclndrftv rrcgvrvinv atgntsleni alvlsldpve

vsgyevlrvs yddlqemdkv lflyiaslfn dedvdfvapl iagidldvss glkvladvsl

isvssngeiv mhslqrqmgk eilhgqsmll sdcessmten lsdvpkkkkk hsesrvkkvv

sipaidegdl wtwrkygqkd ilgsrfprgy yrcaykfthg ckatkqvqrs etdsnmlait

ylsehnhprp tkrkaladst rstsssic

>WRKY41 Oryza sativa Indica

mfnlprrlee llchhgsmlp kgadeeipli kqdleeiisi lhghiltsts mppglridli

fttirefslr iqdalqrham ynnlggvagt asttrgdvcs atpwhptktq frehvdnvrs

vsidvdgmea alndlnklkn llagiptasl vqfrehadkv rgihtdieai lnklenippg

itttttttrg dvsstssrqp tlfmestchv gidaamnkle nlldlcgeek lkvvsivgvg

gvgkttlank lyrklrwqfe crafvrtsqk tdmrrllini llqirshqsp dnwkvhslis

sirtylqdkr gcvccllsli salatirphl akdippikgf lividdlwat stwdiikcal

pegnkssril ttteiedlal qscsydlkfi fkmipfgedd srkllfsivf gshskcppev

setlydivrk cgglplaivt vasllasqld kleqwdyink slgyslmanp tlegmkqlln

lcynnlpqhl kacmlylsmy qgdhiiwkdd lvnqwiaegf icateehdke eisrgyfdel

vgrkiiqpvh iddsgevlsc vvhhivlnfv tyksieenfi iaidhsqati rfadkvrrls

ihfnnvedap pptnmrlfqv rtiaffgvlk ympfimefrl ikvlflhflg dedstgivdl

tkiselvrlr ylkvtsnatv klptrlqglp yletlkidgk isevptdiyl fascnsaenl

wslgelsnlr dlqltyseih sdnlkdnmky lgsilgklrn ltsitlsppg sscpdtlhid

rdtkrrinvd gwssvssppa llqrfellpc vcvfsnlpnw igqlgnlcil kigirevtsn

nidvlgvlph ksvdgretlr rirvkvsstv edgfswvkyg qkdilgtmyp rsyfrcihrh

tkgclatkqv qptdddhqil dviyygehtc dqsarsddrq lkssrpaass nlqepqqpgl

eqsrpaakrr rktvrwktqv rvssvqdvgp lddgyswrry glkdilgaky prsyfrcthr

ntqgcvatkq iqrrdgdpll fdvvyhgdht cseraslneq vtwprssass teqsstityt

aaagsvedde egvtsatnfl smddmldlgg gdvidmdfps fdfdaidafl lg

>RPP13-like protein 2 [Aegilops tauschii]

madmrnfrev vqyltggvpa sdsivqqlvg qgsasqtqdf qiveaveglp relllspsaa

vspaarlasi ersvrpvpaq qpevidltdd rfddgglvav vdddwlslrc asppsppppe

stsgqfspqg qksakqrsqt vsdishsqvk qarqtkkriq qlqapvcama damfrlpekl

dgllsshgqm lprgveeeip likqdlekmv ailqqhddsg aedcamtakc lakevrelsy

dmedsvdqye qaattsrwia prlkkhkfar rrvtrlpekl rwrlwmankm refslrsqea

lqryslfnhh gdngiraaai ggtgsstppr hdtcfgswyp tpyeelvgig ehvnnleawl

grdgeqrlkv vtvvgsggig kstlvkelyr rirgqfecra fvrtsrkpdi rrllismlsq

vrphqtshtw klhsliadir thlqdkryli viddvwatqt wdivsralpd gnlcsgvlit

teiddvalkc ggydsktafg pqyecppels dvanniirkc agfplavvtv agllvnqmgk

peqwdfvnks lgyglrknpa pegmkqvlnl synnlrlhlk aclmylsiye edyiiqkndl

vkqwiaegfi hateekdmve isricfdeli ssrmiepvhi ndtgdvlsct vhhmvldfit

hksleenfvt aidhcqttar ladkvrrlsl hfgnaeatpp tnmrlsqvrt layfgvikcl

psivefgllq ililhlwgdd dsisfdltgi selfrlrylh vtcnatlevp qtqirglryl

etlkidarvs avpsdivhlp gllhlslpve inlpngigrm tslctlecfd isvnsvenvh

slgeltnlhd lrltcstvhs cyltskmdsm csiltklsnl rsltlepssi ldvgpssmsi

scdglssvss ppaclqtfew lprictfssl pkwigrlskl cilkigvrkl anndfdilrg

lpaltvlslh irtkpakril fnkigfsvlk yfkfrcrapw lefevdampn llklklrfda

hgvdqhgtip vgivhltglk emsakiggag andpdrraae salidaikmh pacptlsihc

ldamfsgedd dikkedsieh mtlqkqydvk kedsiehmtl qkqygikked siehttlqeq

ydiknedlie hmtlqiqndi kkedsykqhg flqkdyryda nkhavirktl pkwstqvrvs

slqdieghdd gfswrkygqk dilgsrnprk adpssrrgyy rcthhntrgc qalkqlqatd

gdpllfnaiy vgnhtctqga nsqpqpgyeq ssisvgdkae gsiqrlekmp prrskrsiqv

rvrsmqddyp addgyswsky gqkdilgskh prgyyrcvhr pekgceatkq vqrsdsdtql

fdvvyhgeht caenvhsrge sarslphhvs vsagvippat sesqvtyeav ssgstagihf

mspatsagsq vtyesgsrst ttgrfispgm sesqvayaef wtwpdnvdfm lnspinerld

lnadfvdetg psdfd

>RPM1-like isoform X1 [Setaria italica]

mlfdltdaft fkllglqeis smedqtrpld gtiwklpgkl srllchgcil pkgveneipl

ikhdleeila ilsnldddha mmvrcwrkev relsydiedf inqyehakag swtgsiprrk

iiqrhnsrtt lyrlreklrh rlwiankire fsvrsqevlq rhsmynlnsi agsssrrcag

ahftsshptp cwdgntpigi satmdkleer lmkmydeghq klkvvsivgf ggigkttlan

elyrklgrqf ecraflrtsq kpdmrrilis mlsqvrpqqs pdnwtvhsli stirthlqdk

ryfvivedlw atstwdilkc alpddsccsr ilitteiedl alqscgydpn yifklnplge

ddsrklffss vfgpqqecpp ehrevycdii rkcgglplai vsiasifagq lnvkeqmdyl

nkslgcslit natlegikqv idlsynnlpq hlkacilytg lyeediiiwr ddlvnewiae

gficatggqd kqeisrayfd elvgrkmiqp vhtndngevl scvvhymvln livtyksmee

nfitvvhhsq anstladkvr rlslhfgnae dvippsnmrl sqvrtlvyfg vcrcmpyilq

fhllqvlilh fwgdkdnisl dltriselfr lrylmvssnv tldlrtqmhg lqyletlkid

arvravpsdi vhlsgllnls lptqtnlpsr mghmtslrtl eyfdlstntm envqslitls

nlrelqltcs tvqpenlnnk mqfllnsigr lsnltslnlv prtsscansl ddagaasitv

sdsissmptp tllqslevsp riciflclpk wigqlcklct lkvgvrklar ndidvlrgmp

llavlslyvh tkpasrivig ktgfpviryf kfkccdpllk feedamphlr klklvfnahs

adlqstipvg idflselkef svkiggagpd kshrraaela freaimvhgr cqrvnvrcvq

hiiggkegqs citthddhds yehdeimpdn sgqevlcavk eagtiplpgk kegnndadnr

krrktlvkdi ltsssdgddg hswrkygqke ipgskypray frcthrnsig cnatkqvqrt

dgdpnlfavr yygehtcdyr settvrveme qssitelgvn svnpstssac stssvssrrs

mirgnvktsk pdddgyfwke yvredilgak yprsyyhcvc rhtegcgatk qvqrtdadpl

vfdviyngeh tckqtahftd enkslsvpld pptpglseml saedtaeisf dkfrglfnnd

cpikgkidvd tpwgnmklpi skeggttrik eeddgndn

>XP_002865288 Arabidopsis lyrata

madseqlvci scvsevrysf vshlsealrr kginsviidv dsddllskes qakieissvs

vmvlsricep trvchnfvkv lecqrdknhv vvpvlygesp llgewlsvld lrdlspvhqs

rkecsdsqlv keivrdvyek pfykgrigiy sklleiekmv ckqplgircv giwgmpgigk

ttlakavfdq msgefdascf iedyskaiqe kgvyclleeq flkenaggag gtvtklsllr

dklnnkrvlv vlddvrsplv vesflggfdw fgpksliiit srdksvfrlc rvnqiyevhg

lnekealqlf smcasiddma eqnlhevsmk vikyanghpl alslygrelk gkkrppemet

aflqlkerpp nifvdaiksc ydtlndrekd ifldiacffq genvdyvmqv legcgffphv

gidvlvekyv grhiinretr qtkrrdrlwe pwsikylled ngekengehk ttleraqgpe

eiegmfldts nfsfdikpaa fdnmlnlrll kiyssnpevh hvknflkgfl nslpnelrll

hwenyplqfl pqnfdpihlv einmpysqlk klwggtknle mlktirlchs qqlvdiddvl

kaqnlevidl qgctrlqsfp atgqllhlri vnlsgcteik sfpeippnie tlnlqgtgii

elplsiikpn ytellnllae ipglsgvsnl eqsdlkplts lmkmstsnqn lgkliclelk

dcarlrslpn mnnlellkvl dlsgcselet iqgfpqnlke lylagtavrq vpqlpqslel

fnahgcvslk sirvdfeklp vhytlsncfd lcpkvvsnfl vqalanakri prehqqelnk

tlafsfcaps hanqnskldl qlgssvmtrl npswrntlvg famlvevafs edyydatgfg

iscickwknk eghshriern lhcwalgkav qkdhmfvfcd dnlrpstdeg idpdiwadlv

vfeffpvnnq trllgdsctv trcgvrvitp pncntsleis ssvlsldpme vsgnegeevl

rvsydglqei dkalflylag lfndedidlv aplianiidm dvsyglkvla drslirvssn

geivmynlqr emgkeilhte skktdrlvdn iqssmidske ieithsknrr kksrakkvlw

sieevadqwv wrkygqkpik gspyprnyyk ctsskgcsar kqversrtdp nmlvityise

hnhpsptqrn alagsthsps sskcsavtts dscrvsqhkd epdkshlpss pasppyaamv

ikeedmerrd nmefdddved tlipelfped ffadlnkles nsqtmfqshs ssggnmenqg

rnsssddmga nlpnktl

>GmWRKY176

MASSALTQSSSFSYDVFLSFHATDTRSNFTDFLFQALIRKGIVAFKDESRAPDQAIEDSRLFIVVLSKNYAFSTQCLHEL

SQIFHCVEFSPRRVLPIFYDVDPSDVRKQTGWYEKAFSKYEERFLVNKKGMETVQTWRKALTQVANLSGWYIRNKPQYTE

IEEFVQYTISILSSKFSTLQNKAEGTLSDKDEFSYNELSGEAEGTLSDKDEFSYNELSGEAEGTLSDKDEFSYNELSGLM

RIRDFISLVLTYPVKLVEEDILRSWISAFPEWKAQLLSTGDDTVMDKPWCVLTKKALGSMKGFMPMEIISKVERGRRSIF

SRNDVEAIPEAILHQTVDLAVNQLIQTLFCGDYCSRYIRLLTRNLAEKRYVVDKIVAAVEDKHNMFGIDKDFLRALWINA

STYETDAEFRVQEEINTMMEVDDMWPMEVDNMLGTLFIEEKMGSNGQLVIVVDADSNKKLDLQKLRFPTGIVVLITTEPS

TQAEKDGDFRIACTMDLNIWTQDHLLPWKVFCSYVGSCISSSMVSSSMAIQKIAVEIVKECHGHLLAIVLLSKYLMYVQD

FKQWELALDKLSSLNPSYDYQDSDRIGISRVMVNAFVNIIWEDIDDAQKLCLELSLPVHNIKNGVRDDILVSDWAKIILG

YTQELGEYRRQLQYHMKELLDRFVLLKYESGDVYLPIETYDIIKSLHTSKPSILRHGALGLTEPPYIGRWHSLIRIELMD

NKICELPQSPDCPKLKVLLLQGNVDLMDIPDSFFNHMPLLQHLDLSYTSIRDLPPSVSKLIQLQKFYLRGCDLFMELPHQ

IGQLKNLKELDLDGTLITHLPIEIRELINLQILSLCFDGTSRSTIIPPGLVSNLTQLNYLSINVDPEDEQWNENVISVLG

EIFFGLWNLQRLSIYIPKADLLEFIPAEKSLNFRLVVGQHMRRLISRVPPELETKFKHCDYSIKFVNGVNIPNGVKMNLG

RFKALYLDRHMTIKSLSDFDLRNLWGLEVCILAECNEMETIVSGSNSPDGPASLMLKFLSVFYMKNLRSVCEGSSPFFLF

LKSIALHTCPMLTTIFTLDSLKKLSFLEEIIVEDCPKLTTLISHATPEQKPVFFLPKLRIISLLYLPELVNIFNGMHVGP

FIEEMIFYYCPRLQSLSKSELSSKSLKIIKGESIWWEALKWNEAEWGDAGRPNYFERIFSPINEEADMMNQLAALQETQL

NEYHNTTYQTPAKPQGATKPCTHFFADISHSFQITQHKLNGKKFQEWCQSVLVIKGKGKMCYLTGDISTPKLYCEDMGIS

SSKQSIAKILAPVSSVGRGSSNSEAQKRKVVAEPKIFQEVTKKQLKLLIAVTKKRKEKENITLEMCQSNEGMMHGHISRE

LYACNLKLFVLYFDYASIIPQKIEKGYKRREVWTAKIRVNAEGCYDDGYIWRKYGKRQILGAKYPRYYRCAHKFVHWCPA

RKRVQRLDENPATIEIIYEHRHTCAREQNLIIKSGDEASQSCKL

>WRKY16 AT5G45050.1 MTESEQIVYISCIEEVRYSFVSHLSKALQRKGVNDVFIDSDDSLSNESQSMVERARVSVMILPGNRTVSLDKLVKVLDCQ

KNKDQVVVPVLYGVRSSETEWLSALDSKGFSSVHHSRKECSDSQLVKETVRDVYEKLFYMERIGIYSKLLEIEKMINKQP

LDIRCVGIWGMPGIGKTTLAKAVFDQMSGEFDAHCFIEDYTKAIQEKGVYCLLEEQFLKENAGASGTVTKLSLLRDRLNN

KRVLVVLDDVRSPLVVESFLGGFDWFGPKSLIIITSKDKSVFRLCRVNQIYEVQGLNEKEALQLFSLCASIDDMAEQNLH

EVSMKVIKYANGHPLALNLYGRELMGKKRPPEMEIAFLKLKECPPAIFVDAIKSSYDTLNDREKNIFLDIACFFQGENVD

YVMQLLEGCGFFPHVGIDVLVEKSLVTISENRVRMHNLIQDVGRQIINRETRQTKRRSRLWEPCSIKYLLEDKEQNENEE

QKTTFERAQVPEEIEGMFLDTSNLSFDIKHVAFDNMLNLRLFKIYSSNPEVHHVNNFLKGSLSSLPNVLRLLHWENYPLQ

FLPQNFDPIHLVEINMPYSQLKKLWGGTKDLEMLKTIRLCHSQQLVDIDDLLKAQNLEVVDLQGCTRLQSFPATGQLLHL

RVVNLSGCTEIKSFPEIPPNIETLNLQGTGIIELPLSIVKPNYRELLNLLAEIPGLSGVSNLEQSDLKPLTSLMKISTSY

QNPGKLSCLELNDCSRLRSLPNMVNLELLKALDLSGCSELETIQGFPRNLKELYLVGTAVRQVPQLPQSLEFFNAHGCVS

LKSIRLDFKKLPVHYTFSNCFDLSPQVVNDFLVQAMANVIAKHIPRERHVTGFSQKTVQRSSRDSQQELNKTLAFSFCAP

SHANQNSKLDLQPGSSSMTRLDPSWRNTLVGFAMLVQVAFSEGYCDDTDFGISCVCKWKNKEGHSHRREINLHCWALGKA

VERDHTFVFFDVNMRPDTDEGNDPDIWADLVVFEFFPVNKQRKPLNDSCTVTRCGVRLITAVNCNTSIENISPVLSLDPM

EVSGNEDEEVLRVRYAGLQEIYKALFLYIAGLFNDEDVGLVAPLIANIIDMDVSYGLKVLAYRSLIRVSSNGEIVMHYLL

RQMGKEILHTESKKTDKLVDNIQSSMIATKEIEITRSKSRRKNNKEKRVVCVVDRGSRSSDLWVWRKYGQKPIKSSPYPR

SYYRCASSKGCFARKQVERSRTDPNVSVITYISEHNHPFPTLRNTLAGSTRSSSSKCSDVTTSASSTVSQDKEGPDKSHL

PSSPASPPYAAMVVKEEDMEQWDNMEFDVDVEEDTFIPELFPEDTFADMDKLEENSQTMFLSRRSSGGNMEAQGKNSSDD

REVNLPSKILNR

>WRKY19 AT4G12020.1 MSEKEELPLTLTSIGAATATSDYHQRVGSSGEGISSSSSDVDPRFMQNSPTGLMISQSSSMCTVPPGMAATPPISSGSGL

SQQLNNSSSSKLCQVEGCQKGARDASGRCISHGGGRRCQKPDCQKGAEGKTVYCKAHGGGRRCEYLGCTKGAEGSTDFCI

AHGGGRRCNHEDCTRSAWGRTEFCVKHGGGARCKTYGCGKSASGPLPFCRAHGGGKKCSHEDCTGFARGRSGLCLMHGGG

KRCQRENCTKSAEGLSGLCISHGGGRRCQSIGCTKGAKGSKMFCKACITKRPLTIDGGGNMGGVTTGDALNYLKAVKDKF

EDSEKYDTFLEVLNDCKHQGVDTSGVIARLKDLFKGHDDLLLGFNTYLSKEYQITILPEDDFPIDFLDKVEGPYEMTYQQ

AQTVQANANMQPQTEYPSSSAVQSFSSGQPQIPTSAPDSSLLAKSNTSGITIIEHMSQQPLNVDKQVNDGYNWQKYGQKK

VKGSKFPLSYYKCTYLGCPSKRKVERSLDGQVAEIVYKDRHNHEPPNQGKDGSTTYLSGSSTHINCMSSELTASQFSSNK

TKIEQQEAASLATTIEYMSEASDNEEDSNGETSEGEKDEDEPEPKRRITEVQVSELADASDRTVREPRVIFQTTSEVDNL

DDGYRWRKYGQKVVKGNPYPRFSSSKDYDVVIRYGRADISNEDFISHLRASLCRRGISVYEKFNEVDALPKCRVLIIVLT

STYVPSNLLNILEHQHTEDRVVYPIFYRLSPYDFVCNSKNYERFYLQDEPKKWQAALKEITQMPGYTLTDKSESELIDEI

VRDALKVLCSADKVNMIGMDMQVEEILSLLCIESLDVRSIGIWGTVGIGKTTIAEEIFRKISVQYETCVVLKDLHKEVEV

KGHDAVRENFLSEVLEVEPHVIRISDIKTSFLRSRLQRKRILVILDDVNDYRDVDTFLGTLNYFGPGSRIIMTSRNRRVF

VLCKIDHVYEVKPLDIPKSLLLLDRGTCQIVLSPEVYKTLSLELVKFSNGNPQVLQFLSSIDREWNKLSQEVKTTSPIYI

PGIFEKSCCGLDDNERGIFLDIACFFNRIDKDNVAMLLDGCGFSAHVGFRGLVDKSLLTISQHNLVDMLSFIQATGREIV

RQESADRPGDRSRLWNADYIRHVFINDTGTSAIEGIFLDMLNLKFDANPNVFEKMCNLRLLKLYCSKAEEKHGVSFPQGL

EYLPSKLRLLHWEYYPLSSLPKSFNPENLVELNLPSSCAKKLWKGKKARFCTTNSSLEKLKKMRLSYSDQLTKIPRLSSA

TNLEHIDLEGCNSLLSLSQSISYLKKLVFLNLKGCSKLENIPSMVDLESLEVLNLSGCSKLGNFPEISPNVKELYMGGTM

IQEIPSSIKNLVLLEKLDLENSRHLKNLPTSIYKLKHLETLNLSGCISLERFPDSSRRMKCLRFLDLSRTDIKELPSSIS

YLTALDELLFVDSRRNSPVVTNPNANSTELMPSESSKLEILGTPADNEVVVGGTVEKTRGIERTPTILVKSREYLIPDDV

VAVGGDIKGLRPPVLQLQPAMKLSHIPRGSTWDFVTHFAPPETVAPPSSSSEAREEEVETEETGAMFIPLGDKETCSFTV

NKGDSSRTISNTSPIYASEGSFITCWQKGQLLGRGSLGSVYEGISADGDFFAFKEVSLLDQGSQAHEWIQQVEGGIALLS

QLQHQNIVRYRGTTKDESNLYIFLELVTQGSLRKLYQRNQLGDSVVSLYTRQILDGLKYLHDKGFIHRNIKCANVLVDAN

GTVKLADFGLAKVMSLWRTPYWNWMAPEVIVLKSFPLF
